# Selective Synthesis and Characterization of the Highly Energetic Materials 1‐Hydroxy‐5*H*‐tetrazole (CHN_4_O), its Anion 1‐Oxido‐5*H*‐tetrazolate (CN_4_O^−^) and Bis(1‐hydroxytetrazol‐5‐yl)triazene

**DOI:** 10.1002/asia.202100714

**Published:** 2021-09-02

**Authors:** Thomas M. Klapötke, Moritz Kofen, Laszlo Schmidt, Jörg Stierstorfer, Maximilian H. H. Wurzenberger

**Affiliations:** ^1^ Department of Chemistry Ludwig-Maximilian University of Munich D-81377 Munich Germany

**Keywords:** tetrazoles, explosives, triazenes, diazotation, dediazonization

## Abstract

For the first time, an adequate selective synthesis, circumventing the formation of 2‐hydroxy‐5*H*‐tetrazole, of 1‐hydroxy‐5*H*‐tetrazole (HTO), as well as the synthesis of bis(1‐hydroxytetrazol‐5‐yl)triazene (H_3_T) are reported. Several salts thereof were synthesized and characterized which resulted in the formation of new primary and secondary explosives containing the 1‐oxidotetrazolate unit. Molecular structures are characterized by single‐crystal X‐ray diffraction, ^1^H and ^13^C NMR, IR, and elemental analysis. Calculation of the detonation performance using the explo5 code confirmed the energetic properties of 1‐hydroxy‐5*H*‐tetrazole. The detonation properties can be adjusted to the requirements for those of a secondary explosive by forming the hydroxylammonium (**6**) or hydrazinium (**7**) salts, or to meet the requirements of a primary explosive by forming the silver salt **4**, which shows a fast DDT on contact with a flame. The sensitivities of all compounds towards external stimuli such as impact, friction, and electrostatic discharge were measured.

## Graphical abstract

**The reaction of 5‐amino‐1‐hydroxytetrazole (5‐ATO) with nitrous acid** is strongly dependent on the equivalents of HNO_2_. By applying one equivalent and copper(0)/ethanol it is possible to perform a dediazonization, resulting in 1‐hydroxytetrazole – the highlight of this work. By applying half an equivalent of HNO_2_ it is possible to obtain the azo‐coupled product bis(1‐hydroxytetrazol‐5‐yl)triazene. Both compounds as well as salts thereof are fully characterized in this work.

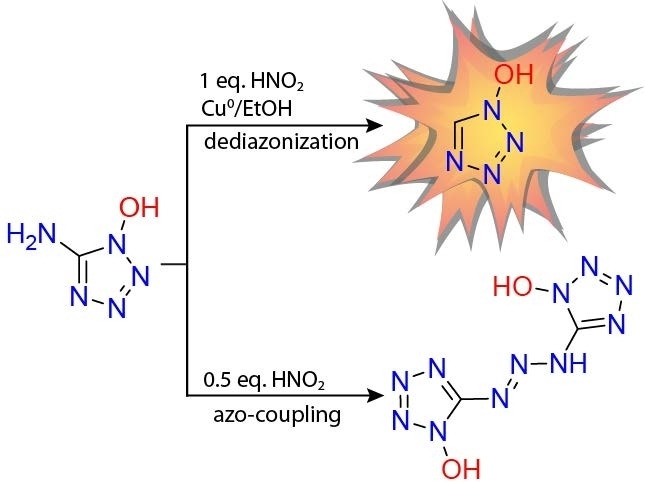

## Introduction

Tetrazoles are a class of heterocycles that exhibit a wide variety of possible applications. A prominent representative is *Losartan*, which is included in the WHO's List of Essential Medicines^[1]^ as a treatment for hypertension.^[2]^ In addition to pharmaceutical uses, tetrazoles show great potential for application as energetic materials with high nitrogen contents like in copper(I) nitrotetrazolate (DBX‐1).[^3^, ^4^] The high endothermic heat of formation of tetrazoles (e. g. +236 kJ mol^−1^ for 1,5*H*‐tetrazole) is advantageous for possible applications as energetic materials, since a large amount of energy is released upon detonation.^[5]^ General protocols to further increase the enthalpy of formation of tetrazoles include *N*‐oxidation by Oxone®,^[6]^ HOF,^[7]^ or H_2_O_2_,^[8]^ which also has the advantage of increasing the oxygen balance and crystal density.^[9]^ In 2012, *Klapötke et al*.^[10]^ obtained dihydroxylammonium‐5,5’‐bistetrazolyl‐1,1’‐diolat (TKX‐50) through the *N*‐oxidation of 5,5’‐bistetrazole followed by formation of the hydroxylammonium salt. This compound possesses excellent detonation properties while being thermally stable (221 °C) and moderately sensitive towards impact (20 J) and friction (120 N), thus being stable enough to be safely used as a high‐performing secondary explosive. TKX‐50 surpasses properties of 1,3,5‐Trinitro‐1,3,5‐triazinan (RDX) and 1,3,5,7‐Tetranitro‐1,3,5,7‐tetrazocan (HMX), which have long been used as the main charge in detonation devices. Even though the 5,5’‐bistetrazole has been known since 1913,^[11]^ it took nearly 100 years until its potential for use as an explosive was discovered in the compound TKX‐50. It is therefore surprising that 1,5*H*‐tetrazole has been known since 1892,^[12]^ but complete characterization of the corresponding *N*‐oxide, namely 1‐hydroxy‐5*H*‐tetrazole, is missing.

**Figure 1:**
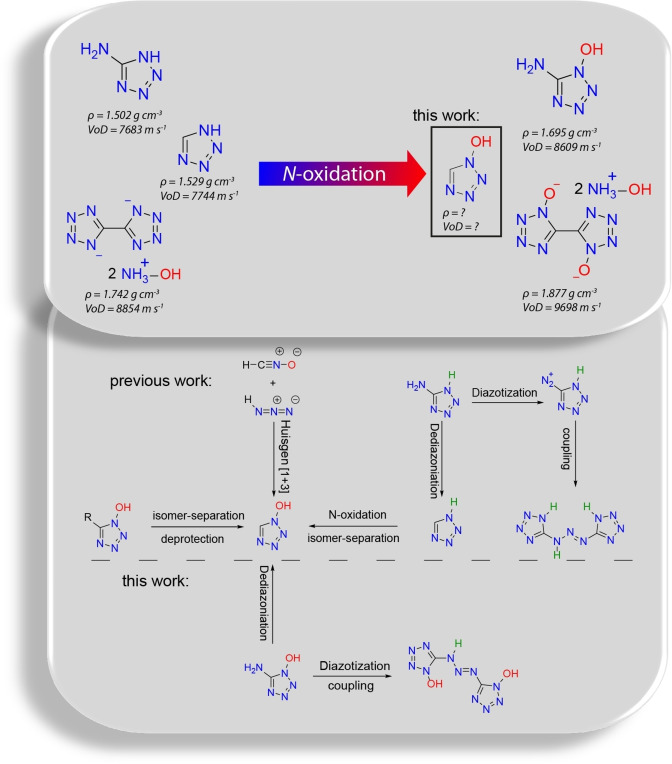
Top: Increasing the density and enhancing the performance of energetic compounds can be achieved by *N*‐oxidation. Bottom: Previous work concerned with the synthesis of 1‐hydroxy‐5*H*‐tetrazole and bis(tetrazol‐5‐yl)triazene.

The first synthesis was reported by *Pallazzo* in 1910 (Figure 1),^[13]^ however, the addition of hydrazoic acid to sodium fulminate is not suitable nor desired for a synthesis on gram‐scale due to the high risks involved when working with fulminates. In 1956, *Bettinetti et al*. successfully synthesized 1‐hydroxy‐5*H*‐tetrazole by the addition of hydrazoic acid to nitrolic acids, which too is not desired for the work on gram‐scale.^[14]^ *Bettinetti et al*. also described the silver salt of 1‐hydroxytetrazole, yet an extensive characterization is missing. In 1995, *Begtrup et al*.^[15]^ used Oxone® to oxidize 1,5*H*‐tetrazole. This idea was then followed by *Giles et al*.^[16]^ in 1999 for oxidizing ethyl tetrazole‐5‐carboxylate. Both procedures result in an isomeric mixture of the 1‐hydroxy‐ and 2‐hydroxy‐tetrazole derivatives, requiring further protection (for the oxidized 1*H*‐tetrazole), as well as separation of the isomers and subsequent secession of the protecting group. In a previous paper in 2013, we showed that the addition of hydroxylamine to a solution of cyanogen azide results in a cyclization forming 5‐amino‐1‐hydroxytetrazole (5‐ATO).^[17]^ In 1954, *Henry et al*. showed that 1,5*H*‐tetrazole can be obtained by the elimination of the amino group of 5‐amino‐1*H*‐tetrazole through diazotization followed by subsequent reduction of the diazonium cation. These so‐called *hydro‐dediazonizations* are performed by boiling a diazonium cation in acidic ethanol. However, this procedure usually results in *ethoxy‐dediazonization*.^[18]^ *Kornblum et al*. later replaced the step in which reduction with acidic ethanol occurs by using hypophosphorous acid as the reducing agent instead.^[19]^

Independently from the applied reduction procedure, the *dediazonization* always starts with a diazotization of the amine, which, for several azoles, can instantly explode and therefore should never be isolated. It can also couple with other compounds and this well‐known behavior is exploited to produce azo dyes, such as Methyl red (2‐(*N*,*N*‐dimethyl‐4‐aminophenyl)^[20]^ or Tartrazine (trisodium 1‐(4‐sulfonatophenyl)‐4‐(4‐sulfonatophenylazo)‐5‐pyrazolone‐3‐carboxylate).^[21]^ Lesser known are triazenes, which result from the coupling of a diazonium cation with an amine. In 1910 *Hofmann et al*. published the first synthesis of sodium bis(tetrazol‐5‐yl)triazene by diazotization of amino guanidinium nitrate with sodium nitrite, intermediately producing 5‐aminotetrazole which then further reacts to the triazene species.^[22]^

When applying the systems analogously to 5‐ATO, the *dediazonization* selectively produces 1‐hydroxy‐5*H*‐tetrazole, thus representing the first adequate selective synthesis for this long over‐due compound, whereas following *Hofmann et al*. results in the formation of bis(1‐hydroxytetrazol‐5‐yl)triazene, the twice *N*‐oxidized bis(tetrazol‐5‐yl)triazene. Therefore, we report the first adequate procedure to obtain 1‐hydroxy‐5*H*‐tetrazole without the need of protection groups and elaborate workups. Additionally, the first synthesis of bis(1‐hydroxytetrazol‐5‐yl)triazene is reported. Both acids as well as salts thereof are prepared and are characterized by single crystal X‐ray diffraction experiments, complemented by NMR and IR spectrometry as well as thermal and physical analysis.

## Results and Discussion

### Synthesis

*Warning! The synthetic work described in this section involves the handling of very sensitive intermediates (diazotetrazole‐1 N‐oxide) and products (e. g., silver salt **4**). Proper protective measurements and equipment must be used*!

**Scheme 1:**
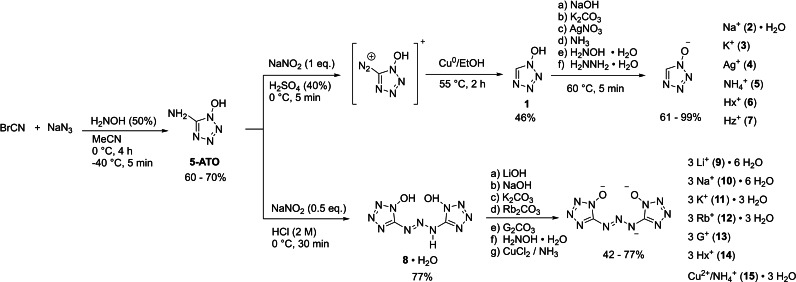
Synthesis of 1‐hydroxy‐5*H*‐tetrazole (**1**) and its salts thereof (**2**–**7**), as well as the synthesis of bis(1‐hydroxytetrazol‐5‐yl)‐triazene (**8**) and its salts thereof (**9**–**15**). Hx^+^: hydroxylammonium; Hz^+^: hydrazinium; G^+^: guanidinium.

The starting material 5‐ATO is readily available from the reaction of cyanogen azide and hydroxylamine.^[17]^ 1‐Hydroxy‐5*H*‐tetrazole (**1**) can be obtained by dissolving 5‐ATO in semi‐concentrated sulfuric acid (40%) and diazotizing with sodium nitrite, while keeping the temperature below 5 °C (Scheme 1). The diazotization solution is then added to a mixture of ethanol and elemental copper and stirred at 55 °C for 2 hours. Due to the intermediate formation of 5‐diazonium‐1‐hydroxytetrazole, the ratio of 5‐ATO to sulfuric acid (40%) should be quite low. A ratio of 1 : 10 was used for the synthesis of all herein investigated compounds, as a higher concentration of 5‐diazonium‐1‐hydroxytetrazole leads to micro‐detonations within the reaction solution. An attempt to reduce the amount of acid to a ratio of 1 : 2 resulted in a violent detonation of the whole reaction solution, destroying the round‐bottom flask. Compound **1** was extracted into DCM, and the ammonium salt (**5**) was subsequently precipitated by passing gaseous ammonia through the organic phase. The sodium (**2**) and potassium (**3**) salts were obtained by refluxing a solution of the corresponding carbonate and **5** in water, followed by evaporating to complete dryness, extracting the residue with ethanol, and precipitating the salts by adding diethyl ether. The silver salt (**4**) can be precipitated by adding silver nitrate to a solution of **1** in water and filtering off the solid. The hydroxylammonium (**6**) and hydrazinium (**7**) salts were obtained by adding the corresponding base to a solution of **1** in ethanol and precipitating with diethyl ether. Due to coextraction of sulfuric acid after *dediazonization* pure **1** is obtained by dissolving **3** in 2 m hydrochloric acid, extracting with ethyl acetate, and removing the solvent *in vacuo*. Bis‐(1‐hydroxytetrazol‐5‐yl)triazene **8** was obtained as a monohydrate by dissolving 5‐ATO in concentrated hydrochloric acid followed by diazotization with half an equivalent of sodium nitrite, adjusting to pH>10 with sodium hydroxide, and extraction into ethyl acetate. Compound **8** crystallized by slow evaporation of the solvent. Salts **9**–**14** were precipitated by adding the corresponding hydroxide (**9**, **10**), carbonate (**11**–**13**), or free base (**14**) dissolved in the minimal volume methanol to a solution of **8** in ethyl acetate. The copper ammonium salt **15** was obtained by precipitating the copper salt of **8** using copper sulfate followed by recrystallization from concentrated ammonia.

When preparing compound **8**, it is important to make sure any trace amounts of the diazonium cation are removed by stirring under basic conditions (pH>10), since it is possible to co‐extract it into the organic solvent. When removing the solvent *in vacuo*, a detonation occurred on slightly touching the solid with a plastic spatula, leading to the person performing the experiment being injured.

### Crystal structures

**Figure 2:**
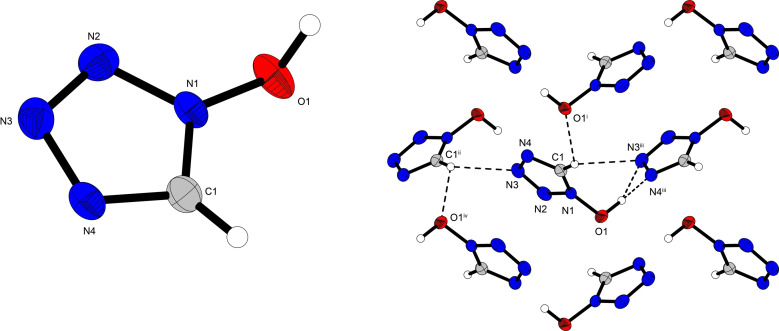
Crystal structure of compound **1** (**A**) as well as crystal packing (**B**) due to hydrogen bonds between four symmetrically distinct 1‐hydroxy‐5*H*‐tetrazole molecules; ellipsoids in all structures are shown with a probability of 50%; Selected bond lengths [Å]: O1−N1 1.354(2), N1−N2 1.337(2), N2−N3 1.299(2), N3−N4 1.353(2), N4−C1 1.316(2), C1−N1 1.324(2); Angles [°]: O1−N1−N2 121.17(11), O1−N1−C1 127.85(12), N1−N2−N3 105.07(11), N2−N3−N4 110.51(12), N3−N4−C1 106.81(12), N4−C1−N1 106.91(12). Selected hydrogen bond lengths [Å] and angles [°] (D−H⋅⋅⋅A, d(D−H), d(H⋅⋅⋅A), d(D⋅⋅⋅A), <(D−H⋅⋅⋅A)): O1−H2⋅⋅⋅N3^iii^: 1.02(2), 1.57(2), 2.5878(16), 178(2); O1−H2⋅⋅⋅N4^iii^: 1.02(2), 2.53(2), 3.4431(18), 150.1(17); C1−H1⋅⋅⋅O1^i^: 0.913(16), 2.600(15), 3.2504(19), 128.8(12); C1^ii^−H1^ii^⋅⋅⋅N3: 0.913(16), 2.543(16), 3.325(2), 144.1(13). Symmetry codes: i) 5/2−x, 1/2 +y, 1/2 −z; ii) 1/2+x, 1/2 −y, 1/2 +z; iii) 1/2 +x, 1/2 −y, −1/2+z.

The solid‐state crystal structure of all of the compounds synthesized in this work, except compound **14**, were determined using low‐temperature single‐crystal X‐ray diffraction. All of the data and parameters of the measurements, as well as of the refinements are given in the Supporting Information Table S1. All crystal densities are recalculated to their respective room temperature crystal density. Crystal datasets were deposited in the CSD database and can be obtained free of charge with the following codes: CCDC 2088975 (**1**), 2088974 (**2 a**), 2088980 (**2 b**), 2088977 (**3**), 2088979 (**4**), 2088985 (**5**), 2088982 (**6**), 2088986 (**7**), 2088988 (**8**), 2088983 (**9**), 2088978 (**10**), 2088976 (**11**), 2088987 (**12**), 2088981 (**13**) and 2088984 (**15**). 1‐Hydroxy‐5*H*‐tetrazole (**1**) crystallizes in the monoclinic space group *P*2_1_/*n*. The density of **(1)** (1.63 g cm^−3^) is comparable to that of the parent molecule 5‐amino‐1‐hydroxytetrazole (1.66 g cm^−3^)^[17]^ and is significantly higher than that of 1‐aminotetrazole (1‐AT, 1.48 g cm^−3^).^[23]^ Figure 2 shows the crystal structure of **1**, which forms wave‐like layers in an ABA layering (along the b and c‐axis) due to the two H atoms present per molecule participating in a total of three hydrogen bonds to two different 1‐hydroxy‐5*H*‐tetrazole molecules – one of which lies within the same wave‐like layer (A), whereas the other is from a different layer (B) (Figure 2B).

Interestingly, the respective C−N and N−N bond lengths within the tetrazole ring are nearly equivalent for compound **1** compared to 5‐ATO and 1‐AT. The bond length between the hydroxy group and the tetrazole ring in **1** is the same as that reported for 5‐ATO, but is slightly shorter than the corresponding bond of the amine in 1‐AT, thus explaining the higher density. All of the bond lengths are in the typical range for N−N, C−N, and N−O single bonds, as well as N−N and C−N double bonds. The tetrazole ring in **1** is planar, with the oxygen atom slightly protruding from the plane (O1−N1−N2−N3=5°). In all of the salts of **1** investigated in this work, a planar arrangement of the oxygen atom with respect to the tetrazole‐plane was observed for the 1‐oxido‐5*H*‐tetrazolate anions.

**Figure 3:**
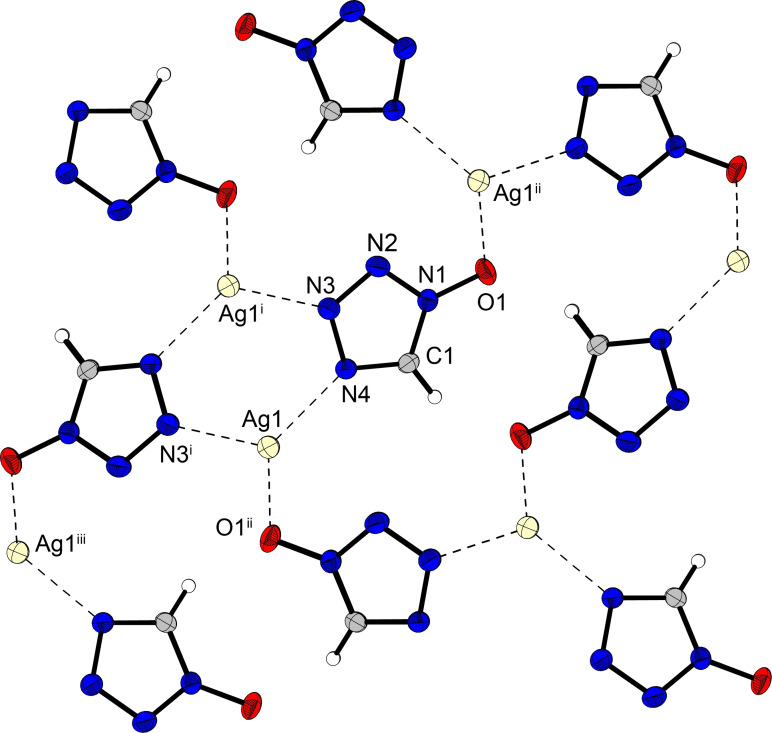
Crystal structure of silver 1‐oxido‐5*H*‐tetrazolate (**4**) showing the coordination environment of the silver cations. Selected bond distances [Å]: N4−Ag1^i^: 2.235(3), N3−Ag1^ii^: 2.275(3), O1−Ag1^iii^: 2.628(8), O1^ii^−Ag1^iv^: 2.406(7); angles [°]: O1^ii^−Ag1−N4 124.97(12), N3^i^−Ag1−N4 122.14(11), O1^ii^−Ag1−N3^i^ 81.73(12); Symmetry codes: i) −x, 1−y, 1/2 +z; ii) 1/2 −x, 1/2 +y, 1/2 +z; iii) −1/2+x, 1/2 −y, z.

Sodium 1‐oxido‐5*H*‐tetrazolate (**2**) crystallizes as a monohydrate in two polymorphs, one in the triclinic space group *P*1 (**2 a**) with a density of 1.81 g cm^−3^ and one in the orthorhombic space group *P*2_1_2_1_2_1_ (**2 b**) with a slightly lower density of 1.78 g cm^−3^. The triclinic form is obtained by crystallization from water, whereas the orthorhombic polymorph from ethanol. Deprotonation of **1** results in marginal changes in the bond lengths of the tetrazole ring, with the N1−O1 bond length being generally shortened by 0.03–0.04 Å for all investigated salts in comparison with the neutral compound **1**. Potassium (**3**) and silver 1‐oxido‐5*H*‐tetrazolate (**4**) both crystallize in the orthorhombic space group *Pna*2_1_ with densities of 1.88 g cm^−3^ (**3**) and 3.48 g cm^−3^ (**4**), respectively. Figure 3 shows the crystal structure of **4**, where all silver cations are threefold coordinated by individual anions bridging between the cations.

**Figure 4:**
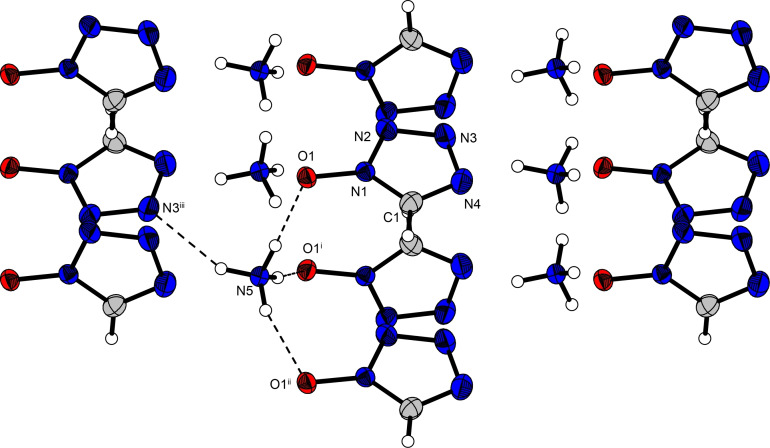
Packing of ammonium 1‐oxido‐5*H*‐tetrazolate (**5**) showing layering due to the presence of hydrogen bonds between the hydrogen atoms of an ammonium cation with four distinct tetrazolate anions. Selected hydrogen bond lengths [Å] and angles [°] (D−H⋅⋅⋅A, d(D−H), d(H⋅⋅⋅A), d(D⋅⋅⋅A), <(D−H⋅⋅⋅A)): N5−H3⋅⋅⋅O1: 0.93(2), 1.86(2), 2.7762(18), 172(2); N5−H2⋅⋅⋅O1^i^: 0.91(2), 1.90(2), 2.8036(19), 171(2); N5−H5⋅⋅⋅O1^ii^: 0.90(3), 2.04(3), 2.9063(18), 162(2); N5−H4⋅⋅⋅N3^iii^: 0.88(3), 2.18(2), 2.976(2), 151(2). Symmetry codes: i) 1/2+x,1/2−y, z; ii) x, −1+y, z; iii) 1−x, 1−y, −1/2+z.

Ammonium 1‐oxido‐5*H*‐tetrazolate (**5**) also crystallizes in the orthorhombic space group *Pna*2_1_ with four molecular units in the unit cell and a density of 1.40 g cm^−3^. The crystal packing shows the presence of strong hydrogen bonds involving all of the protons of the ammonium cations (Figure 4), with bonds towards three oxygen atoms and one nitrogen atom of four different 1‐oxidotetrazolate anions. The observed density is the lowest of all of the herein investigated compounds, and remarkably, it is even lower than that of comparable compounds such as ammonium 1‐oxido‐5‐aminotetrazolate (1.50 g cm^−3^)^[17]^ and ammonium 5‐azidotetrazolate (1.61 g cm^−3^).^[24]^

**Figure 5:**
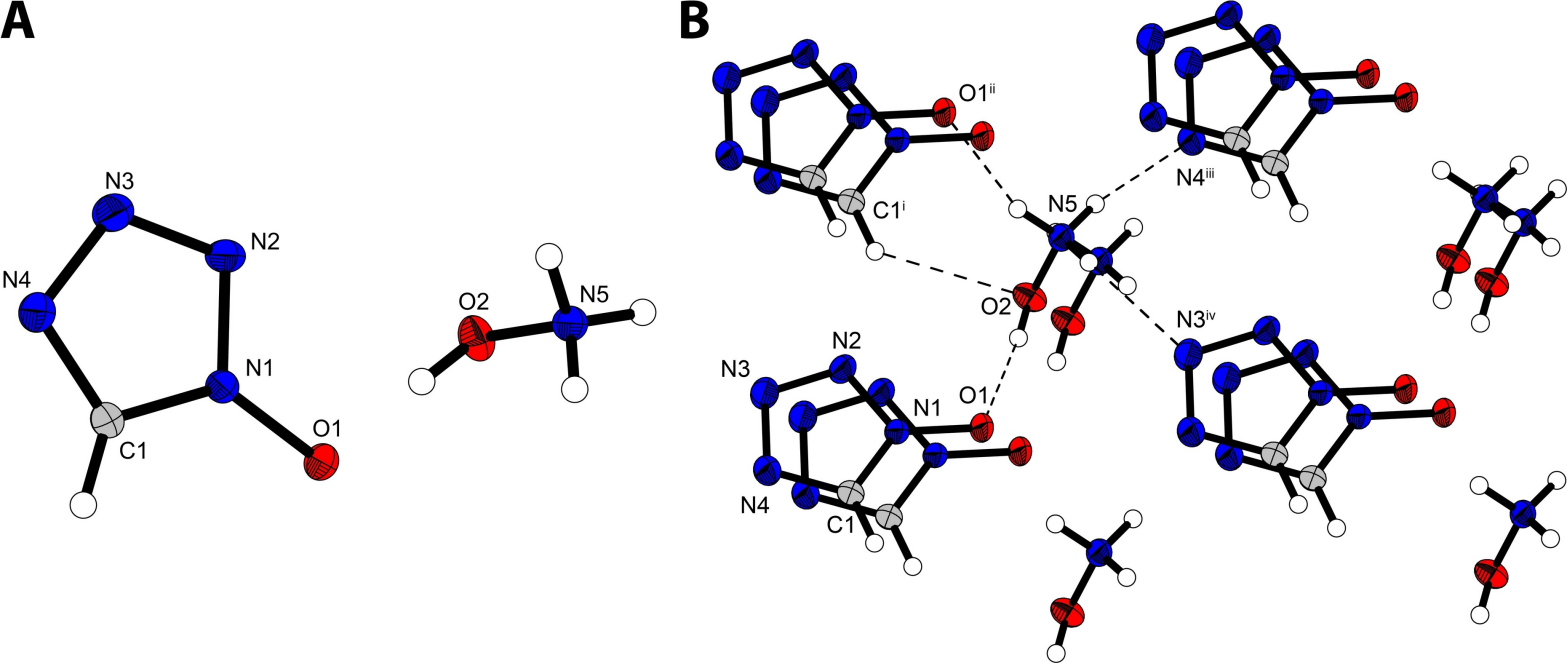
A) Crystal structure of hydroxylammonium 1‐oxidotetrazolate (**6**). B) Packing due to the presence of strong hydrogen bonds involving of all the protons. Selected bond lengths [Å]: O1−N1 1.319(2), N1−N2 1.343(3), N2−N3 1.304(3), N3−N4 1.350(3), N4−C1 1.328(3), C1−N1 1.332(3), N5−O2 1.411(2); Angles [°] O1−N1−N2 122.13(2), N1−N2−N3 106.19(18), N2−N3−N4 110.74(18), N3−N4−C1 105.86(18), N4−C1−N1 108.36(18), C1−N1−O1 129.02(18); Selected hydrogen bond lengths [Å] and angles [°] (D−H⋅⋅⋅A, d(D−H), d(H⋅⋅⋅A), d(D⋅⋅⋅A), <(D−H⋅⋅⋅A)): O2−H2⋅⋅⋅O1: 0.82(4), 1.76(4), 2.576(2), 172(4); C1^i^−H1^i^⋅⋅⋅O2: 0.92(3), 2.57(3), 3.274(2), 134(2); N5−H5 A⋅⋅⋅N3^iv^: 0.81(3), 2.18(3), 2.913(3), 151(3); N5−H5B⋅⋅⋅N4^iii^: 0.84(3), 2.05(3), 2.883(3), 170(3); N5−5 C⋅⋅⋅O1^ii^: 0.88(3), 1.93(3), 2.766(2), 160(3). Symmetry codes: i)−1+x, 1+y, z ii) x,1+y, z iii) −1+x, 1+y, −1+z iv) x, y, −1+z.

Hydroxylammonium 1‐oxido‐5*H*‐tetrazolate (**6**) crystallizes in the triclinic space group *P*1 with a density of 1.67 g cm^−3^ (298 K) with one molecular unit in the unit cell as shown in Figure 5. Although all of the protons participate in hydrogen bonds, the density of **6** is significantly lower than that of comparable molecules such as dihydroxylammonium dinitro‐bis‐1,2,4‐triazole‐1,1’‐diol (MAD‐X1) (1.90 g cm^−3^ at 298 K)^[6]^ or TKX‐50 (1.92 g cm^−3^ at 298 K).^[10]^

**Figure 6:**
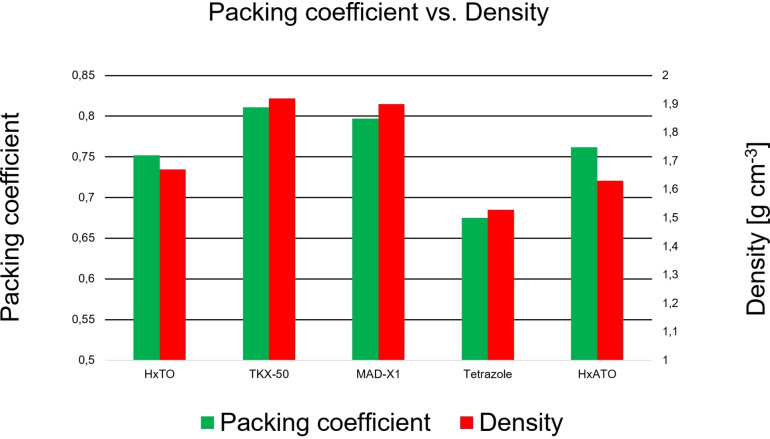
Packing coefficient and crystal densities of compound **6** (HxTO) and comparable compounds such as MAD‐X1, TKX‐50, tetrazole and hydroxylammonium 5‐amino‐1‐oxidotetrazolate (HxATO).

The lower density is also indicated by a lower packing coefficient^[25]^ (Figure 6) of **6** (0.752) compared to TKX‐50 (0.811) and MAD‐X1 (0.797). Interestingly, the packing coefficient of **6** is comparable to the hydroxylammonium salt of 5‐ATO (0.762) but is significantly higher than that for 1,5*H*‐tetrazole (0.675). This confirms the positive effect of *N*‐oxidation on the density as stated by Fischer *et al*.,^[17]^ whereas amination at the carbon position does not lead to significant changes in density. Generally, it can be assumed that the introduction of a bicycle (TKX‐50, MAD‐X1) is beneficial as it increases the packing coefficient, and thus increases the density.

**Figure 7:**
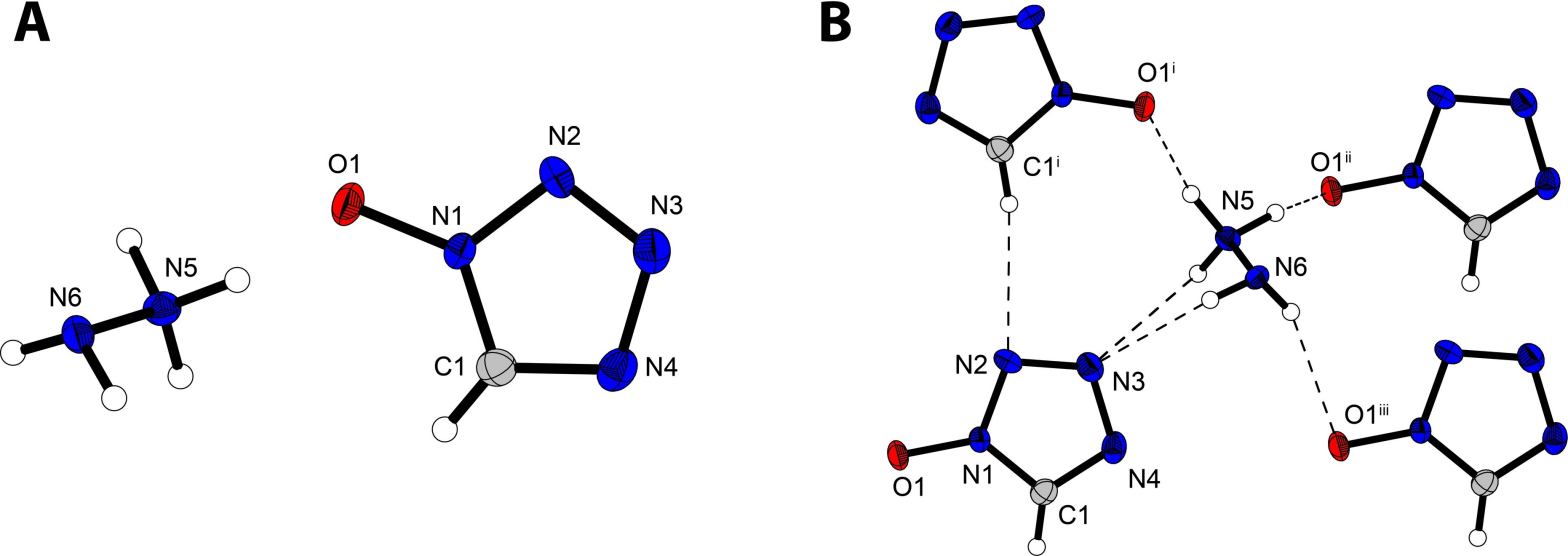
A) Crystal structure of hydrazinium 1‐oxido‐5*H*‐tetrazolate (**7**). B) Packing due to strong hydrogen bonds of all protons, linking four distinct anions by one hydrazinium cation. Selected bond lengths [Å]: O1−N1 1.329(3), N1−N2 1.3336(3), N2−N3 1.316(4), N3−N4 1.344(4), N4−C1 1.329(4), C1−N1 1.329(4), N5−N6 1.443(4); Angles [°] O1−N1−N2 120.8(2), N1−N2−N3 105.7(2), N2−N3−N4 111.0(3), N3−N4−C1 105.6(3), N4−C1−N1 108.6(3), C1−N1−O1 130.1(3); Selected hydrogen bond lengths [Å] and angles [°] (D−H⋅⋅⋅A, d(D−H), d(H⋅⋅⋅A), d(D⋅⋅⋅A), <(D−H⋅⋅⋅A)): C1^i^−H1^i^⋅⋅⋅N2: 0.88(4), 2.59(4), 3.453(4), 165(3); N5−H5 A⋅⋅⋅O1^i^: 0.97(4), 1.80(5), 2.742(4), 165(4); N5−H5B⋅⋅⋅O1^ii^: 0.99(4), 1.94(4), 2.826(4), 148(4), N5−H5 C⋅⋅⋅N3: 0.89(4), 2.45(4), 3.047(4), 125(3); N6−H6 A⋅⋅⋅N3: 0.91(3), 2.50(3), 3.147(4), 128(3), N6−H6B⋅⋅⋅O1^iii^: 0.81(4), 2.36(4), 3.112(4), 156(3). Symmetry codes: i)−1+x, 1−y, −1/2+z ii) −1+x,−1+y, z iii) x, −1+y, z.

Hydrazinium 1‐oxido‐5*H*‐tetrazolate (**7**) crystallizes in the monoclinic space group *Pc* with two molecular units in the unit cell and a density of 1.6 g cm^−3^, following the trend observed of hydrazinium salts having lower densities compared to the corresponding hydroxylammonium salts. The crystal structure of **7** is shown in Figure 7A, in which all hydrogen atoms are participating in hydrogen bonds resulting in one N_2_H_5_^+^ cation being linked to four anions (Figure 7B).

**Figure 8:**
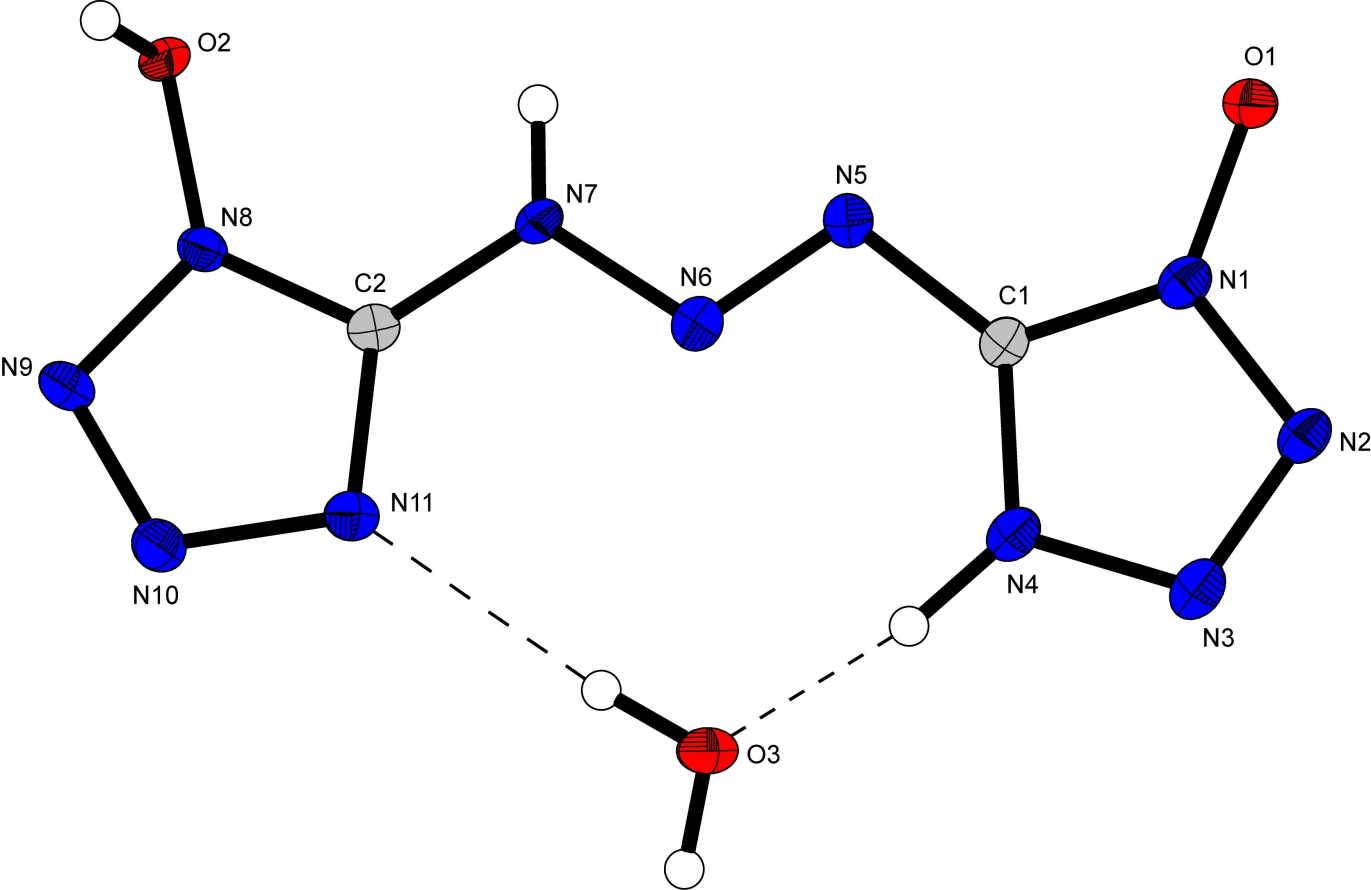
Crystal structure of bis(1‐hydroxytetrazol‐5‐yl)triazene monohydrate (**8**), showing the zwitterionic form. Selected bond lengths [Å]: O1−N1 1.321(2), N8−O2 1.349(2), N3−N4 1.338(2), N4−C1 1.337(2), C1−N5 1.382(2), N5−N6 1.269(2), N6−N7 1.317(2), N7−C2 1.368(2), N11−N10 1.361(2), N11−C2 1.318(2); Angles [°] O1−N1−N2 121.8(1), O1−N1−C1 128.3(1), O2−N8−N9 123.5(1), O2−N8−C2 126.6(1), N3−N4−C1 109.4(2), N10−N11−C2 104.7(1), C1−N5−N6 107.9(1), C2−N7−N6 114.8(1).

**Figure 9:**
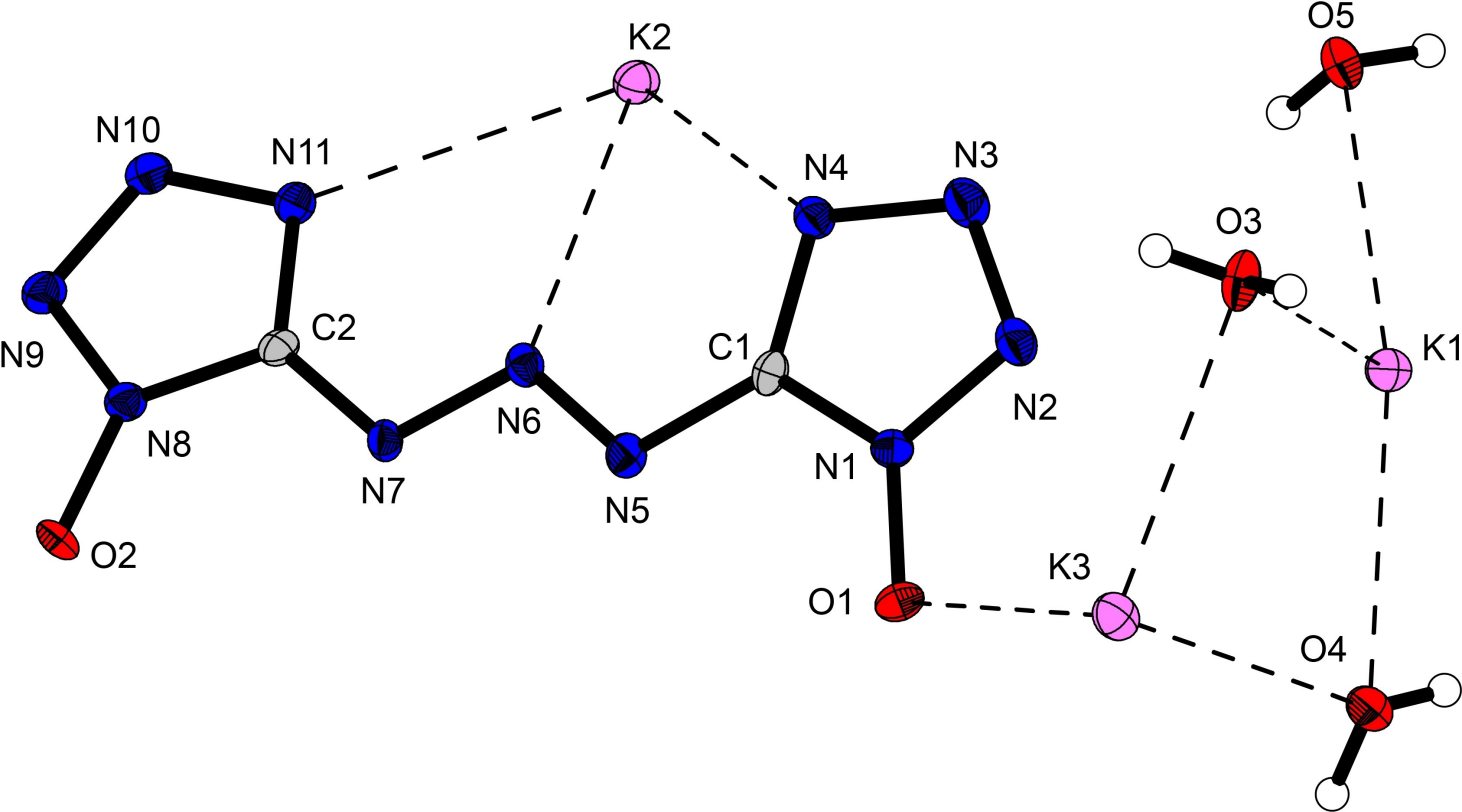
Crystal structure of tripotassium bis(1‐oxidotetrazol‐5‐yl)triazenide trihydrate (**11**). Selected bond lengths of the anion [Å]: O1−N1 1.330(3), N1−N2 1.352(4), N2−N3 1.311(3), N3−N4 1.359(3), N4−C1 1.340(4), C1−N1 1.339(4), C1−N5 1.384(4), N5−N6 1.309(3), N6−N7 1.309(3), N7−C2 1.375(4), C2−N8 1.346(4), N8−O2 1.330(3), N8−N9 1.346(2), N9−N10 1.316(4), N10−N11 1.359(3), N11−C2 1.340(4); Angles [°]: O1−N1−N2 122.0(2), O1−N1−C1 129.1(3), O2−N8−N9 122.8(2), O2−N8−C2 127.7(2), C1−N4−N3 105.5(2), C2−N11−N10 105.9(3), N6−N5−C1 111.4(3), N6−N7−C2 111.1(2).

Bis‐(1‐hydroxytetrazol‐5‐yl)triazene (**8**) crystallizes in the monoclinic space group *P*2_1_/*n* as a monohydrate with a density of 1.80 g cm^−3^ and four molecular units in the unit cell (Figure 8). Due to the high acidity of the hydroxy group next to the azo‐moiety, **8** crystallizes in a zwitterionic form where one tetrazole ring (N4) is protonated by its own hydroxy group (O1). While the self‐protonation only leads to small differences in bond lengths between the two 1‐hydroxytetrazol units, changes in bond angles between the two moieties of up to 4.7° (N3−N4−C1=109.4(2), N10−N11−C2=104.7(1)) are observed. The largest difference in bond lengths and angles is observed for the triazene bridge. Within the triazene bridge, the N5−N6 bond (1.269(2) Å) is significantly shorter than the N6−N7 bond (1.327(2) Å), suggesting a higher bond order and therefore more double bond character for the N5−N6 bond. Additionally, the zwitterionic ring is more strongly bent towards the triazene bridge (N6−N5−C1=107.9(1)°) than its counterpart (N6−N7−C2=114.8(1)°). Compounds **9** and **10** both crystallize as hexahydrates in the triclinic space group *P*‐1 with a density of 1.67 g cm^−3^ and 1.71 g cm^−3^, respectively. Tripotassium bis(1‐oxidotetrazol‐5‐yl)triazenide (**11**) crystallizes as a trihydrate in the orthorhombic space group *P*2_1_2_1_2_1_ with a density of 1.98 g cm^−3^ (Figure 9). While complete deprotonation of **8** doesn't appear to lead to significant changes in bond lengths within the tetrazole rings, the double bond character of the N5‐N6 bond in the triazene‐bridge has now been more delocalized, which is indicated by the equality of the N5−N6 and N6−N7 bond lengths which are both 1.309(3) Å in **11**. The most drastic change in the structure of the anion resulting from complete deprotonation of **8** is the arrangement of the 1‐oxidotetrazolate moieties with respect to the triazenide bridge. While the two moieties are not equally bent towards the triazene bridge in **8**, after complete deprotonation, the anion in **11** now shows two 1‐oxidotetrazolate units which are oriented in the same way towards the triazenide bridge.

Compounds **12** and **13** both crystallize as trihydrates. It was not possible to obtain crystals of compound **14** suitable for single crystal X‐ray diffraction experiments. The crystal structures of compounds **9, 10**, **12** and **13** are shown in the Supporting Information (Figures S1‐S4).

**Figure 10:**
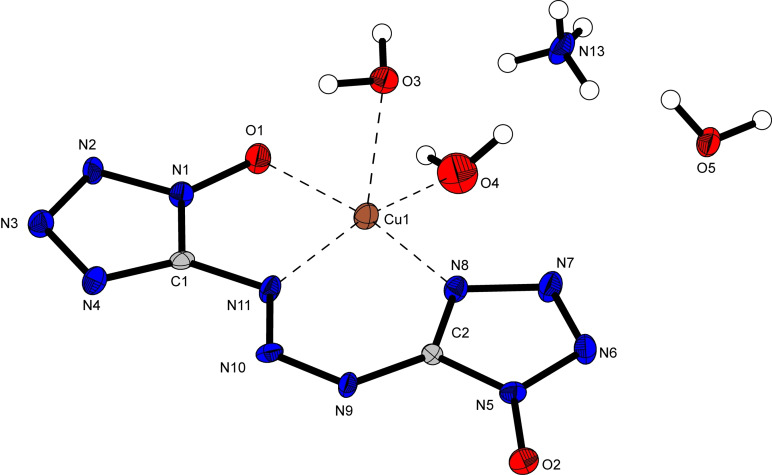
Crystal structure of ammonium copper bis(1‐oxidotetrazol‐5‐yl)triazenide trihydrate (**15**). Selected bond lengths [Å]: O1−N1 1.347(6), N1−N2 1.341(6), N2−N3 1.321(6), N3−N4 1.374(6), N4−C1 1.323(7), C1−N1 1.338(7), C1−N5 1.379(6), N5−N6 1.343(6), N6−N7 1.284(6), N7−C2 1.386(6), C2−N8 1.354(7), N8−O2 1.305, N8−N9 1.352(6), N9−N10 1.304(6), N10−N11 1.351(6), N11−C2 1.336(7), Cu1−O1 1.982(4), Cu1−N11 1.993(4), Cu1−N8 1.938(4), Cu1−O3 2.332(4), Cu1−O4 1.977(5); Angles [°]: O1−N1−C1 125.1(4), O2−N8−C2 128.4(4), C1−N5−N6 113.8(4), N6−N7−C2 121.3(4), O1−Cu1−O3 89.19(14), O1−Cu1−O4 93.85(17), O1−Cu1−N8 169.87(16), O1−Cu1−N11 85.17(15), O3−Cu1−N8 95.84(16), O3−Cu1−N11 104.42(16), O3−Cu1−O4 91.82(17).

Ammonium copper bis(1‐oxidotetrazol‐5‐yl)triazenide (**15**) crystallizes in the triclinic space group *P*‐1 with two molecular units in the unit cell and a density of 1.96 g cm^−3^ (Figure 10). As was observed for compound **11**, the bond lengths and angles of the anion are not significantly influenced by deprotonation. The drastic conformational change observed in **11** is a result of the unequal coordination of the copper(II)‐cation with the two 1‐oxidotetrazolate moieties. Whereas the most favored conformation for compounds **8**–**14** is apparently a triazene bridge with a double (*E*‐) arrangement along N5−N6 and N6−N7, threefold coordination of the copper(II) cation by the anion in **15** results in rotation along the N6−N7 bond, resulting in a *E/Z‐* conformation along N5−N6 and N6−N7, respectively. Interestingly, the copper(II) cation shows a nearly perfectly square pyramidal coordination by the anion and two aqua ligands. The angles involving O3 and atoms forming the equatorial plane (O1, N8, N11, O4) range from 89.13° (O1−Cu1−O3) to 104.42° (O3−Cu1−N11).

### NMR spectroscopy

**Figure 11:**
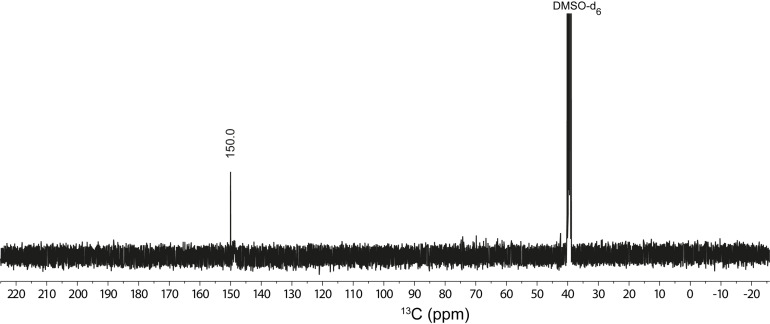
^13^C NMR spectrum of compound **8** in DMSO‐d_6_, showing only one carbon atom.

NMR spectroscopy was performed in DMSO‐d_6_, D_2_O or acetone‐d_6_ and spectra are depicted in the Supporting Information. The ^1^H NMR spectrum of compound **1** in DMSO‐d_6_ shows one signal at δ=9.48 ppm for the proton attached to the carbon atom of the tetrazole ring. The ^13^C NMR of compound **1** in DMSO‐d_6_ shows one signal at δ=137.7 ppm, which is shifted upfield compared to the starting material 5‐ATO (δ=150.5 ppm). Deprotonation of **1** leads to a shift in the signals observed in the ^1^H as well as ^13^C NMR spectra. For example, the ^1^H NMR of potassium 1‐oxido‐5*H*‐tetrazolate (**3**) in D_2_O shows one signal at δ=8.54 ppm, which is shifted upfield compared to the free acid **1**. Additionally, the signal in the ^13^C NMR of **3** in D_2_O is shifted downfield to δ=164.5 ppm. The ^13^C NMR of compound **8** in DMSO‐d_6_ shows one signal at δ=150.0 attributing both carbon atoms (Figure 11). Compared to the parent molecule, 5‐ATO (δ=150.5 ppm), the carbon atoms of **8** are, contrary to **1**, not drastically shifted. Deprotonation of **8** results in a downfield shift of the signal. The ^13^C NMR of the tripotassium salt (**11**) in D_2_O shows one signal at δ=153.2 ppm. The influence of deprotonation of **8** on the ^13^C NMR signal is not as prominent as that observed for compound **1**.

### Physicochemical properties

**Figure 12:**
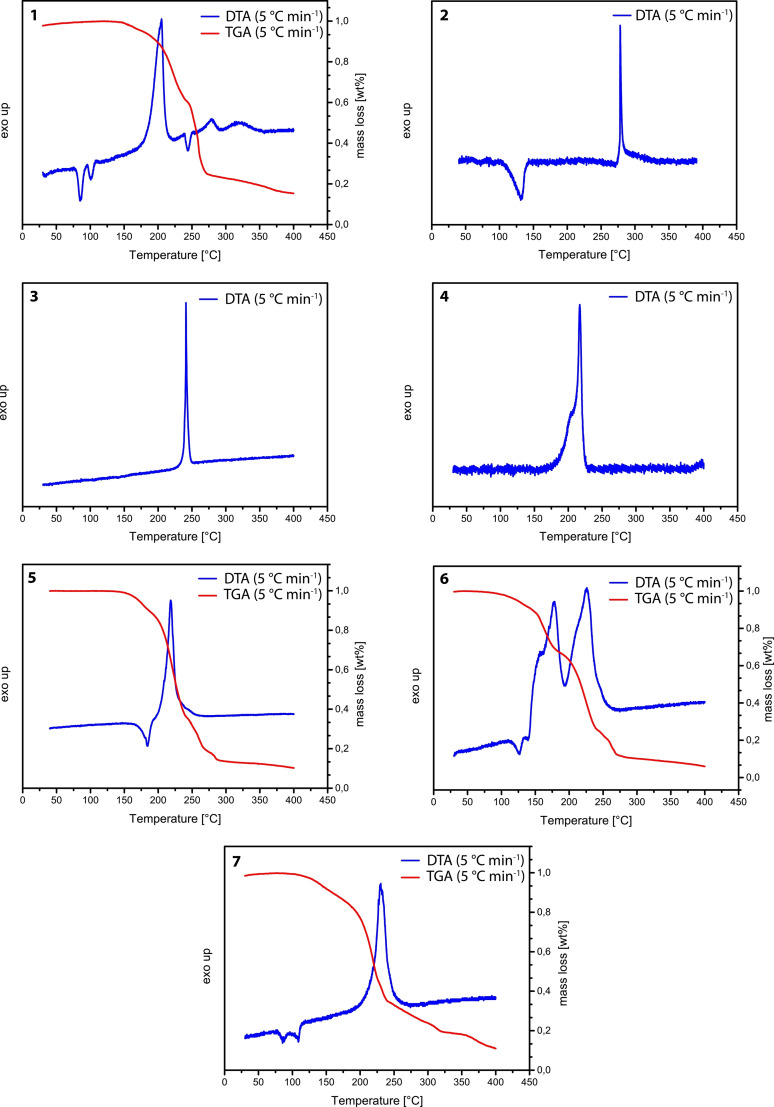
Thermal analytic measurements (DTA) of HTO (**1**), NaTO ⋅ H_2_O (**2**), KTO (**3**), AgTO (**4**), NH_4_TO (**5**), HxTO (**6**), HzTO (**7**). For compounds **1**, **5**, **6**, and **7**, additional TGA measurements were performed to analyze the endothermic events observed in the DTA.

*Thermal behavior*. The thermal behaviors of compounds **1**–**7** are shown in Figure 12. Compound **1** shows a decomposition temperature of 186 °C (Figure 12), and two endothermic events at 80 °C and 96 °C, which TGA indicates as corresponding to a phase transition and/or melting point. Compound **1**, 1‐hydroxy‐5*H*‐tetrazole, exhibits a higher thermal stability compared to the similar compound 5‐amino‐1‐hydroxytetrazole (105 °C),^[17]^ and is almost identical with that of 1‐aminotetrazole (182 °C).^[23]^ Compound **2** shows loss of water at 110 °C and a decomposition temperature of 273 °C, which is the highest of all of the 1‐hydroxy‐5*H*‐tetrazole salts reported in this work (Figure 12). The potassium (**3**) and the silver (**4**) salts, which are both free of water, show decomposition temperatures of 236 °C and 211 °C, respectively. Both of these salts detonate violently on reaching their critical temperatures. The silver salt (**4**) also immediately detonates on contact with a flame. Compound **5** shows an endothermic event at 180 °C, which corresponds to the loss of ammonia due to evaporation, as indicated by the onset of mass loss in the TG. The endothermic event seamlessly evolves into an exothermic decomposition at 188 °C, at which temperature a significant mass loss of 85.5 wt.% occurs as shown by the TG (Figure 12). The same behavior is observed for the hydroxylammonium salt **6**. Figure 12 shows an endothermic event at 115 °C, which evolves into the first of two exothermic decomposition events at 159 °C. TGA measurements revealed the first decomposition to correspond to the decomposition of hydroxylamine accompanied by a weight loss of 24.9 wt.%. The second exothermic event with onset at 203 °C is attributed to the decomposition of the residual 1‐hydroxy‐5*H*‐tetrazole.

**Figure 13:**
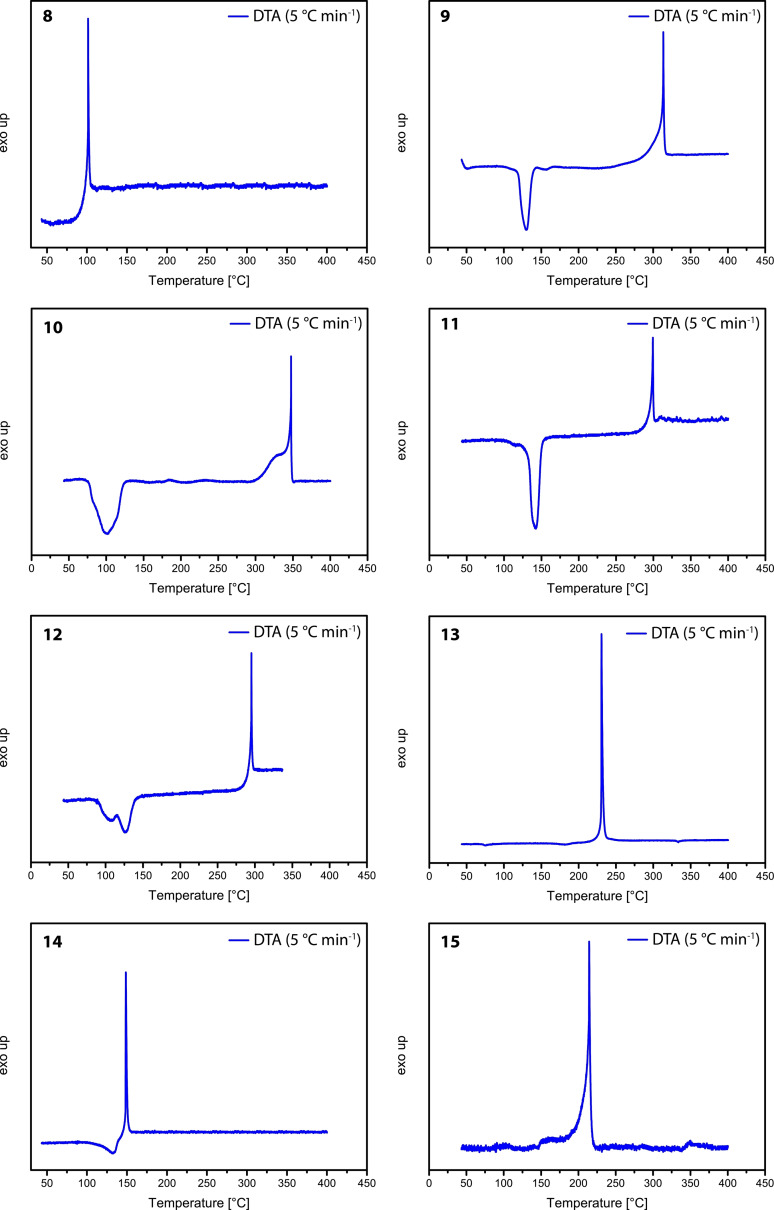
DTA measurements of bis‐(1‐hydroxytetrazol‐5‐yl)triazene monohydrate (**8**), Li_3_T ⋅ 6 H_2_O (**9**), Na_3_T ⋅ 6 H_2_O (**10**), K_3_T ⋅ 3 H_2_O (**11**), Rb_3_T ⋅ 3 H_2_O (**12**), Gua_3_T (**13**), Hx_3_T (**14**), Cu(NH_4_)T (**15**).

Compound **7** shows an exothermic decomposition temperature of 213 °C, with two endothermic events in addition observed at 80 °C and 103 °C. The TG measurement of **7** shows a mass loss of 13 wt.% with onset at 103 °C, which corresponds to the evaporation of hydrazine. Therefore, it can be assumed that the exothermic event actually corresponds to thermal decomposition of residual neutral compound **1**, rather than the hydrazinium salt itself. Figure 13 shows that bis(1‐hydroxytetrazol‐5‐yl)triazene monohydrate (**8**) is thermally stable up to 95 °C. Despite being a monohydrate, no endothermic event which could be attributed to a loss of water was observed in the DTA spectrum of **8**. Due to stabilization by crystal water, **8** decomposes immediately on reaching a temperature (95 °C) high enough to remove the crystal water molecule. The alkaline salts **9**–**12** all show the presence of endothermic events between 85–135 °C corresponding to the loss of water in each compound (Figure 13). In general, these compounds are very thermally stable, with decomposition temperatures of between 292 °C (**12**) and 335 °C (**10**). Interestingly, the triguanidinium salt **13** shows no endothermic event in the DTA, which confirms loss of water already occurring at room temperature. This agrees with the measured elemental analysis, which fits perfectly with the values calculated for the anhydrous salt, which shows a thermal stability of up to 222 °C. The hydroxylammonium salt **14** shows an endothermic event at 104 °C which evolves into an exothermic decomposition, a behavior similar to that of compound **5** (Figure 12). This can be explained by the evaporation of hydroxylamine occurring, which results in the formation of an unstable residue that immediately decomposes at 148 °C. Interestingly, compound **15** shows no loss of water until the onset of decomposition at 198 °C, indicating strong coordinative bonds of the aqua ligands to the copper(II) cation as well as a high stability of the crystal water.

**Figure 14:**
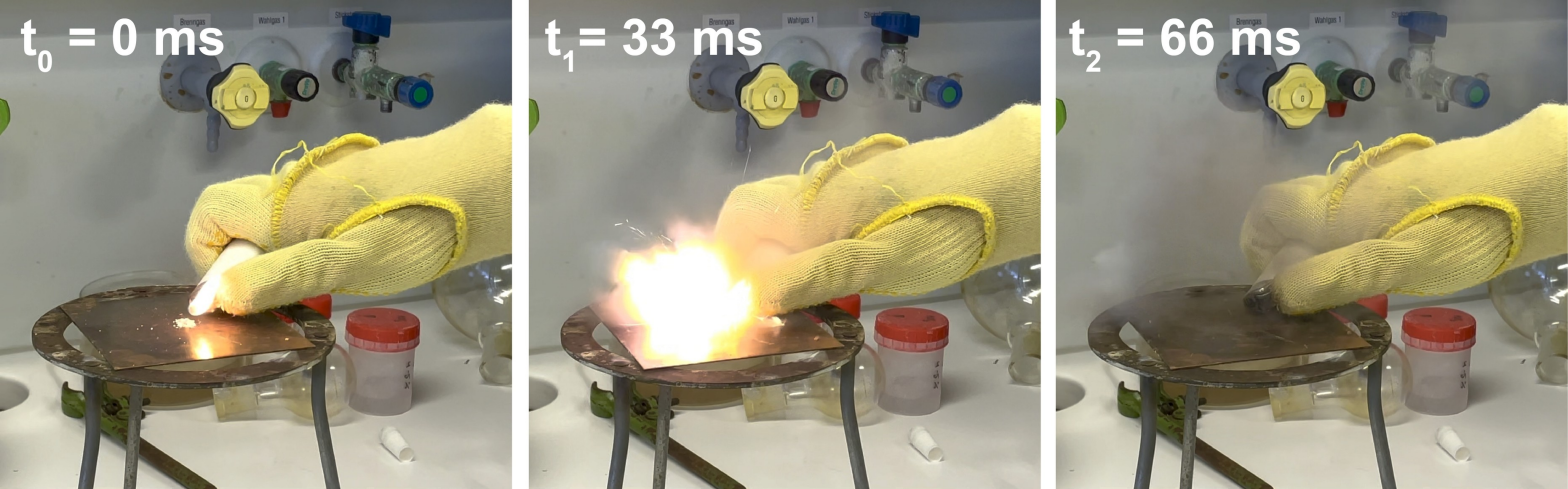
Initiation of silver 1‐oxido‐5*H*‐tetrazolate (**4**) by a lighter. t_0_) right before the flame is in contact with the compound; t_1_) immediate detonation of **4** on contact with the flame; t_2_) smoke residue after detonation.

*Heats of formation, sensitivity, and detonation parameters*. The calculated and measured explosive properties of all compounds are listed in Tables 1 and 2. Heat of formations were calculated by applying the atomization method using room temperature CBS‐4 M enthalpies.[^24^, ^25^, ^26^, ^27^, ^28^] A detailed explanation of the calculation can be found in the Supporting Information. Compound **1** shows a comparable heat of formation (252 kJ mol^−1^) to its parent molecule 5‐amino‐1‐hydroxytetrazole (256 kJ mol^−1^). However, **1** possesses a slightly lower density than its parent compound, which results in a minor reduction of the detonation velocity (8405 m s^−1^) and detonation pressure (269 kbar) compared to 5‐ATO (8609 m s^−1^, 298 kbar). While the impact sensitivity of **1** (10 J) is comparable to that of 5‐ATO (10 J), the friction sensitivity is higher for **1** (28 N) than for 5‐ATO (108 N), meaning **1** is more sensitive towards external stimuli than 5‐ATO. In contrast, the hydroxylammonium (**6**) salt of 1‐hydroxy‐5*H*‐tetrazole shows a higher heat of formation (259 kJ mol^−1^ (**6**)) compared to the corresponding salt of 5‐ATO (Hx^+^: 227 kJ mol^−1^). Compound **6** shows a very high detonation velocity (9284 m s^−1^) compared to compound **5** (8013 m s^−1^), which is due to the higher density of **6** 1.67 g cm^−3^ (**5**: 1.40 g cm^−3^). Although compound **7** shows a slightly lower density (1.60 g cm^−3^) than compound **6**, the significantly higher enthalpy of formation of **7** (346 kJ mol^−1^) results in its even higher detonation velocity of 9437 m s^−1^. Compounds **6** and **7** both outperform even HMX in terms of detonation velocity. While compound **5** is completely insensitive (IS>40 J, FS>360 N), compounds **6** (IS=6 J, FS 240 N) and **7** (IS=26 J, FS>360 N) show higher impact sensitivities, but crucially, are comparable to (**6**) or less sensitive (**7**) than HMX. The monohydrate sodium salt (**2**) is completely insensitive towards external stimuli, whereas the potassium (**3**) salt is more sensitive (IS=4 J, FS=54 N). The silver salt (**4**) is the most sensitive out of all of the compounds, and has to be classified as extremely sensitive, with an impact sensitivity below 1 J and a friction sensitivity of 1 N. The silver salt shows the characteristics of a primary explosive, immediately detonating on contact with a flame (Figure 14).

**Table 1:** Energetic properties of 1‐hydroxy‐5*H*‐tetrazole (**1**) and its salts **2**–**7**.

	**1**	**2 a**	**2 b**	**3**	**4**	**5**	**6**	**7**	**HMX^[q]^**
Formula	CH_2_N_4_O	CH_3_N_4_O_2_Na	CH_3_N_4_O_2_Na	CHN_4_OK	CHN_4_OAg	CH_5_N_5_O	CH_5_N_5_O_2_	CH_6_N_6_O	C_4_H_8_N_8_O_8_
*M* [g mol^−1^]	86.05	126.05	126.05	124.14	192.91	103.09	119.08	118.10	296.16
*IS* [J]^[a]^	10	>40	>40	4	<1	>40	6	26	7^[31]^
*FS* [N]^[b]^	28	>360	>360	54	1	>360	240	>360	112^[31]^
*ESD* [mJ]^[c]^	960	1080	1080	63	<0.28	>1500	>1500	740	200^[31]^
*ρ* [g cm^−3^]^[d]^	1.63	1.81	1.78	1.88	3.47	1.40	1.67	1.60	1.91
*N* [%]^[e]^	65.11	44.45	44.45	45.13	29.04	67.94	58.81	71.16	37.84
*Ω_CO_* [%]^[f]^	−18.59	−12.36	−12.36	−32.22	−8.29	−38.80	−20.15	−40.64	0.0
*Ω_CO2_* [%]^[g]^	−37.19	−25.39	−25.39	−45.11	−16.59	−54.33	−33.59	−54.19	−21.61
*T_endo_* [°C]^[h]^	80, 96	110	110	–	–	180	115	80, 103	–
*T_exo_* [°C]^[i]^	186	273	273	236	211	188	159, 203	213	275
*Δ_f_H* ^*0*^ [kJ mol^−1^]^[j]^	252	−366	−364	47	–	207	259	346	75
*Δ_f_H* ^*0*^ [kJ kg^−1^]^[k]^	3042	−2803	−2780	441	–	2170	2325	3100	369
explo5 v6.05.04									
−*Δ_Ex_U* ^*0*^ [kJ kg^−1^]^[l]^	5333	2792	2817	3265	–	4663	6137	5409	5699
*T_det_* [K]^[m]^	3744	2070	2088	2669	–	2978	3666	3114	3622
*V_0_* [L kg^−1^]^[n]^	469	451	456	426	–	549	461	485	401
*P_CJ_* [kbar]^[o]^	269	189	182	187	–	213	331	319	378
*V_det_* [m s^−1^]^[p]^	8405	7555	7428	7056	–	8013	9284	9437	9192

[a] Impact sensitivity (BAM drophammer (1 of 6)). [b] Friction sensitivity (BAM friction tester (1 of 6)). [c] Electrostatic discharge device (OZM research). [d] From X‐ray diffraction analysis recalculated to 298 K. [e] Nitrogen content. [f] Oxygen balance towards CO formation [g] Oxygen balance towards CO_2_ formation [h] Temperature of endothermic event (DTA; β=5 °C min^−1^). [i] Temperature of exothermic event (DTA; β=5 °C min^−1^). [j] Calculated enthalpy of formation. [k] Calculated mass related enthalpy of formation. [l] Energy of explosion. [m] Detonation temperature. [n] Volume of detonation products (assuming only gaseous products). [o] Detonation pressure at Chapman‐Jouguet point. [p] Detonation velocity. [q] Values based on the explo5 Database.

**Table 2:** Energetic properties of bis‐(1‐hydroxytetrazol‐5‐yl)triazene monohydrate (**8**) and its salts **9**–**15**.

	**8**	**9**	**10**	**11**	**12**	**13**	**14**	**15**
Formula	C_2_H_5_N_11_O_3_	C_2_H_12_N_11_O_8_Li_3_	C_2_H_12_N_11_O_8_Na_3_	C_2_H_6_N_11_O_5_K_3_	C_2_H_6_N_11_O_5_Rb_3_	C_5_H_18_N_20_O_2_	C_2_H_12_N_14_O_5_	C_2_H_10_N_12_O_5_Cu
*M* [g mol^−1^]	234.14	339.01	387.16	381.45	520.55	390.34	312.22	345.73
IS [J]^[a]^	<1	>40	>40	>40	>40	>40	4	11
FS [N]^[b]^	4	>360	>360	>360	288	>360	128	288
*ρ* [g cm^−3^]^[c]^	1.80	1.67	1.71	1.97	2.55	1.59^[p]^	1.79^[p]^	1.96
*N* [%]^[d]^	66.66	45.45	39.80	40.39	29.60	71.77	62.81	48.62
*Ω_CO_* [%]^[e]^	−10.38	−7.08	−6.20	−25.17	−9.22	−49.19	−15.37	−13.88
*Ω_CO2_* [%]^[f]^	−24.23	−16.52	−14.46	−33.56	−15.37	−69.69	−25.62	−23.14
*T_endo_* [°C]^[g]^	–	122	85	135	99, 121	–	123	–
*T_exo_* [°C]^[h]^	100	311	339	294	292	222	146	205
*Δ_f_H°* [kJ mol^−1^]^[i]^	734	−1505	−1480	−1475	–	158	464	−53
*Δ_f_H°* [kJ kg^−1^]^[j]^	3248	−4303	−3705	−3786	–	559	1633	−38
explo5 v6.05.04								
−*Δ_Ex_U* ^*0*^ [kJ kg^−1^]^[k]^	5896	3568	3003	1239	–	2106	5330	3173
*T_det_* [K]^[l]^	3940	2208	2150	1316	–	1736	3299	2654
*V_0_* [L kg^−1^]^[m]^	429	476	474	420	–	501	437	402
*P_CJ_ [kbar]* ^[n]^	362	213	173	143	–	201	364	249
*V_det_[m s* ^−1^ *]* ^[o]^	9433	7941	7352	6546	–	7870	9663	7725

[a] Impact sensitivity (BAM drophammer (1 of 6)). [b] Friction sensitivity (BAM friction tester (1 of 6)). [c] From X‐ray diffraction analysis recalculated to 298 K. [d] Nitrogen content. [e] Oxygen balance towards CO formation [f] Oxygen balance towards CO_2_ formation [g] Temperature of endothermic event (DTA; β=5 °C min^−1^). [h] Temperature of exothermic event (DTA; β=5 °C min^−1^). [i] Calculated enthalpy of formation. [j] Calculated mass related enthalpy of formation. [k] Energy of explosion. [l] Detonation temperature. [m] Volume of detonation products (assuming only gaseous products). [n] Detonation pressure at Chapman‐Jouguet point. [o] Detonation velocity. [p] Density measured pycnometrically.

**Figure 15:**
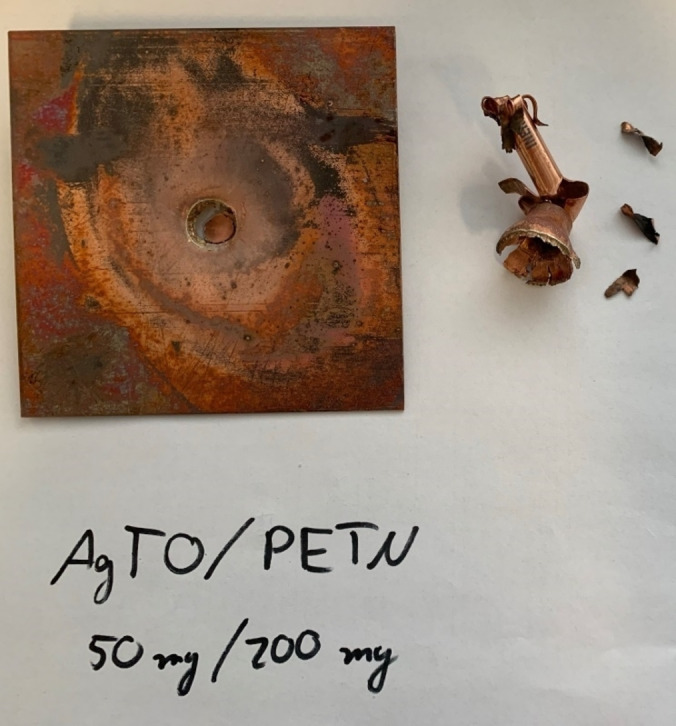
Positive initiation of PETN (200 mg) by **4** (50 mg) indicated by the perforated witness plate.

Due to this behavior, the initiation capability of **4** towards Pentaerythrityltetranitrat (PETN) was tested, whereby it was found that 50 mg of **4** were able to initiate 200 mg of pressed PETN (Figure 15). Compound **2 a** shows a low detonation velocity of 7555 m s^−1^, which is attributed to its low heat of formation as well as being a monohydrate. The orthorhombic form **2 b** exhibits a slightly lower detonation velocity (7428 m s^−1^) than triclinic **2 a**, due to the lower density of **2 b**. Interestingly, the potassium salt **3** exhibits an even lower detonation velocity than **2 a/b**, even though it has a higher density (1.88 g cm^−3^). Due to the high heat of formation of compound **8** (734 kJ mol^−1^), it shows an outstanding detonation velocity of 9433 m s^−1^, as well as a very high detonation temperature (3940 K), which even outperforms HMX. However, in addition to its low thermal stability (decomposition temperature of only 100 °C), compound **8** is also very sensitive towards impact (<1 J) and friction (4 N), preventing application of **8** as a high‐performance secondary explosive.

Complete deprotonation of **8** forming the alkali salts **9** (Li^+^), **10** (Na^+^), and **11** (K^+^) results in a drastic decrease in energetic performance. This is due to the presence of a large amount of crystal water which results in highly exothermic enthalpy of formations (−1475–−1505 kJ mol^−1^). Additionally, the absence of protons, not included as crystal water, leads to a decrease in detonation velocity within the series of increasing cation weight. Compound **13**, crystallizes as a trihydrate, but completely loses its crystal water by drying in air at room temperature, as confirmed by elemental analysis as well as IR spectroscopy. Therefore, the heat of formation and detonation properties were calculated for the anhydrous compound, showing an endothermic heat of formation (158 kJ mol^−1^). The rather low density for **13** of 1.59 g cm^−3^, detonation velocity of 7870 m s^−1^ and detonation pressure of 201 kbar means it lies in the range of the other alkali salts. Compound **14** was obtained as an amorphous solid which is thermally stable up to 146 °C. It has an endothermic heat of formation of 464 kJ mol^−1^ and density of 1.79 g cm^−3^, with a calculated detonation velocity of 9663 m s^−1^, which surpasses that of HMX. Compound **14** has a calculated detonation pressure of 364 kbar as well as moderate sensitivities to external sources (IS=4 J, FS=128 N), which are comparable to those of HMX.

## Conclusion

For the first time, an adequate selective synthesis, circumventing the formation of 2‐hydroxy‐5*H*‐tetrazole, of 1‐hydroxy‐5*H*‐tetrazole is reported by the *dediazonization* of the readily available precursor, 5‐amino‐1‐hydroxytetrazole. Diazotization was followed by reduction of the diazonium cation using either hypophosphorous acid or a mixture of ethanol/copper(0). While ethanol/copper(0) was inferior to hypophosphorous acid in terms of yields, this procedure was chosen for the synthesis of 1‐hydroxy‐5*H*‐tetrazole (**1**), as it is far easier to workup. Diazotization of 5‐ATO with half an equivalent of sodium nitrite resulted in the formation of bis(1‐hydroxytetrazol‐5‐yl)triazene monohydrate (**8**). Compound **1** was converted to the sodium (**2**), potassium (**3**), silver (**4**), ammonium (**5**), hydroxylammonium (**6**) and hydrazinium (**7**) salts. Compound **8** was converted to the lithium (**9**), sodium (**10**), potassium (**11**), rubidium (**12**), guanidinium (**13**), hydroxylammonium (**14**), and ammonium copper(II) (**15**) salts. All compounds, except **14**, were analyzed using single crystal X‐ray diffraction, as well as NMR, IR, and EA analysis. The thermal stabilities were investigated, sensitivities towards external stimuli were determined, and the detonation performances were calculated using the EXPLO5 code. Compound **1** shows a good detonation performance (8405 m s^−1^) which can compete with that of RDX. The detonation performance, thermal stability and sensitivities of **1** could be adjusted to meet the requirements of a primary explosive by formation of the silver salt (**4**), or to those of a highly energetic secondary explosive by formation of **6** (VoD=9284 m s^−1^) or **7** (VoD=9437 m s^−1^), which surpass the detonation velocity of even HMX (9192 m s^−1^). Bis(1‐hydroxytetrazol‐5‐yl)triazene monohydrate (**8**) is a highly sensitive (IS<1 J, FS=4 N) material, with the performance of a high energetic secondary explosive (VoD=9433 m s^−1^). Salts of **8** are completely insensitive due to the incorporation of up to six equivalents of crystal water (for the lithium salt), which is accompanied by a drastic loss of performance. The hydroxylammonium salt (**14**) is an anhydrous compound (as proven by elemental analysis), which shows a very high detonation velocity of 9663 m s^−1^ but with impact and friction sensitivities comparable to those of PETN (IS=4 J, FS=80 N).

## Experimental Section

**1‐Hydroxy**‐**5*H***‐**tetrazole** (**1**): 5‐Amino‐1‐hydroxytetrazole (1.00 g, 10 mmol) was dissolved in sulfuric acid (16 mL, 40%) and cooled below 5 °C with an ice‐bath. Sodium nitrite (684 mg, 10 mmol) dissolved in water (20 mL) was slowly added keeping the temperature below 5 °C. After complete addition the reaction solution was stirred for 5 minutes, and was then added to a flask containing a mixture of elemental copper (628 mg, 10 mmol) and ethanol (20 mL) which was warmed to 55 °C. After stirring for 2 hours, the reaction mixture was extracted by DCM/Et_2_O (1 : 1, 200 mL), the organic phase was dried over magnesium sulfate and the solvent was removed *in vacuo*. The yellow oil which was obtained contained 1‐hydroxy‐5*H*‐tetrazole and residual sulfuric acid as a by‐product. Pure 1‐hydroxy‐5*H*‐tetrazole was obtained by dissolving the potassium salt (**3**) in hydrochloric acid (2 M), followed by extraction into ethyl acetate. After drying the organic phase over MgSO_4_, the solvent was removed by a stream of nitrogen gas, and 1‐hydroxy‐5*H*‐tetrazole (**1**) was obtained as a colorless crystalline solid in moderate yield (400 mg, 4.65 mmol, 46%).

^**1**^ **H NMR** (acetone‐*d*_6_, 25 °C): δ=9.23 ppm (s, 1H, C*H*); ^**13**^ **C NMR** (acetone‐*d*_6_, 25 °C): δ=137.5 ppm (N4‐*C*); **IR** (ATR, cm^−1^): $\tilde{\nu }$ =3001 (w), 2952 (w), 2924 (w), 1652 (vs), 1575 (s), 1416 (s), 1351 (s), 1284 (vs), 1244 (s), 1214 (vs), 1106 (w), 1059 (s), 1004 (m), 976 (s), 952 (s), 902 (s), 841 (vs), 750 (vs), 737 (s), 695 (vs), 667 (s), 635 (s), 588 (s); **DTA** (5 °C min^−1^): 186 °C (T_exo_); **elemental analysis** calcd. [%] for CH_2_N_4_O (86.05): C 13.96, H 2.34, N 65.11; found: C 14.15, H 2.31, N 64.96; **BAM drophammer**: 10 J, **BAM friction tester**: 28 N, **ESD**: 960 mJ.

**Sodium 1‐oxido‐5*H*‐tetrazolate monohydrate** (**2**): Compound **1** (500 mg, 5.81 mmol) was dissolved in methanol (10 mL) and sodium hydroxide (232 mg, 5.81 mmol) in methanol was added. After stirring for 5 minutes, the precipitate was filtered off and recrystallized from ethanol. Sodium 1‐oxido‐5*H*‐tetrazolate monohydrate (**2**) was obtained as yellow crystals in quantitative yield (625 mg, 5.78 mmol, 99%).

^**1**^ **H NMR** (DMSO‐*d*_6_, 25 °C): δ=8.07 ppm (s, 1H, C*H*); ^**13**^ **C NMR** (DMSO‐*d*_6_, 25 °C): δ=133.1 ppm (N4‐*C*); **IR** (ATR, cm^−1^): $\tilde{\nu }$ =3465 (m), 3427 (m), 3281 (w), 3150 (m), 1652 (m), 1472 (m), 1433 (vw), 1407 (m), 1384 (w), 1350 (vw), 1309 (w), 1247 (s), 1192 (m), 1158 (w), 1114 (w), 1065 (w), 982 (w), 824 (m), 738 (m), 715 (w), 670 (m), 529 (s), 481 (vs); **DTA** (5 °C min^−1^): 273 °C (T_exo_); **elemental analysis** calcd. [%] for CH_3_N_4_NaO_2_ (126.05): C 9.53, H 2.40, N 44.45; found: C 9.71, H 2.45, N 43.54; **BAM drophammer**: >40 J, **BAM friction tester**: >360 N, **ESD**: 1080 mJ.

**Potassium 1‐oxido‐5*H*‐tetrazolate** (**3**): Compound **5** (500 mg, 4.85 mmol) was dissolved in water (10 mL) and potassium carbonate (670 mg, 4.85 mmol) was added. The solvent was completely dried off in air, and the solid obtained was extracted with hot ethanol and filtered. After adding diethyl ether, pure potassium 1‐oxido‐5*H*‐tetrazolate (**3**) was filtered off and obtained as a yellow powder in good yield (518 mg, 4.17 mmol, 86%). Crystals suitable for single crystal X‐Ray measurements were obtained by recrystallization from water.

^**1**^ **H NMR** (DMSO‐*d*_6_, 25 °C): δ=8.45 ppm (s, 1H, C*H*); ^**13**^ **C NMR** (DMSO‐*d*_6_, 25 °C): δ=135.1 ppm (N4‐*C*); **IR** (ATR, cm^−1^): $\tilde{\nu }$ =1632 (m), 1622 (m), 1475 (m), 1435 (w), 1402 (m), 1386 (m), 1369 (m), 1352 (w), 1290 (s), 1234 (vs), 1187 (s), 1137 (m), 1124 (w), 1098 (m), 1067 (m), 1012 (m), 968 (m), 923 (w), 874 (w), 836 (m), 817 (s), 781 (w), 731 (m), 718 (m), 690 (s), 675 (m), 645 (m), 625 (w), 585 (w), 477 (m); **DTA** (5 °C min^−1^): 236 °C (T_exo_); **elemental analysis** calcd. [%] for CHKN_4_O (124.14): C 9.68, H 0.81, N 45.13; found: C 9.93, H 1.03, N 44.96; **BAM drophammer**: 4 J, **BAM friction tester**: 54 N, **ESD**: 63 mJ.

**Silver 1‐oxido‐5*H*‐tetrazolate** (**4**): Compound **1** (150 mg, 1.74 mmol) was dissolved in ethanol (10 mL) and silver nitrate (296 mg, 1.74 mmol) was added. After stirring for several minutes, the precipitate was filtered off and washed with ethanol (10 mL) and diethyl ether (10 mL). Silver 1‐oxido‐5*H*‐tetrazolate (**4**) was obtained as a white powder in quantitative yield (332 mg, 1.72 mmol, 99%). Crystals suitable for single crystal X‐Ray measurements were obtained by a three‐layer diffusion crystallization over several days.

**IR** (ATR, cm^−1^): $\tilde{\nu }$ =3100 (m), 1454 (s), 1414 (vs), 1404 (s), 1371 (w), 1340 (w), 1301 (w), 1246 (vs), 1206 (s), 1159 (m), 1108 (s), 1058 (w), 995 (w), 859 (s), 810 (vw), 732 (s), 701 (m), 667 (m), 495 (s); **DTA** (5 °C min^−1^): 211 °C (T_exo_); **elemental analysis** calcd [%] for CHAgN_4_O (192.91): C 6.23, H 0.52, N 29.04; found: C 6.11, H 0.63, N 28.38; **BAM drophammer**: <1 J, **BAM friction tester**: 1 N, **ESD**: <0.28 mJ.

**Ammonium 1‐oxido‐5*H*‐tetrazolate** (**5**): Compound **5** was obtained by passing gaseous ammonia through a solution of **1** in DCM/Et_2_O. The precipitate in the DCM/Et_2_O solution was filtered off, extracted with methanol (40 mL), prior to diethyl ether being added. The precipitate was filtered off and dried in air. Ammonium 1‐oxidotetrazolate (**5**) was obtained as an off‐white powder in moderate yield (629 mg, 6.11 mmol, 61%). Crystals suitable for single crystal X‐Ray measurements were obtained by recrystallization from water.

^**1**^ **H NMR** (DMSO‐*d*_6_, 25 °C): δ=8.44 ppm (s, 1H, C*H*); ^**13**^ **C NMR** (DMSO‐*d*_6_, 25 °C): δ=135.1 ppm (N4‐*C*); **IR** (ATR, cm^−1^): $\tilde{\nu }$ =3465 (m), 3427 (m), 3281 (w), 3150 (m), 1652 (m), 1472 (m), 1433 (vw), 1407 (m), 1384 (w), 1350 (vw), 1309 (w), 1247 (s), 1192 (m), 1158 (w), 1114 (w), 1065 (w), 982 (w), 824 (m), 738 (m), 715 (w), 670 (m), 529 (s), 481 (vs); **DTA** (5 °C min^−1^): 188 °C (T_exo_); **elemental analysis** calcd [%] for CH_5_N_5_O (103.09): C 11.65, H 4.89, N 67.94; found: C 11.26, H 4.89, N 68.58; **BAM drophammer**: >40 J, **BAM friction tester**: >360 N, **ESD**: >1500 mJ.

**Hydroxylammonium 1‐oxido‐5*H*‐tetrazolate** (**6**): Compound **1** (500 mg, 5.81 mmol) was dissolved in ethanol (10 mL) and aqueous hydroxylamine solution (50%, 479 mg, 7.20 mmol) was added. After stirring for 5 minutes at 50 °C, the precipitate was filtered off, washed with diethyl ether and dried in air. Hydroxylammonium 1‐oxido‐5*H*‐tetrazolate (**6**) was obtained as a yellowish powder in good yield (546 mg, 4.59 mmol, 79%). Crystals suitable for single crystal X‐Ray measurements were obtained by recrystallization from water.

^**1**^ **H NMR** (DMSO‐*d*_6_, 25 °C): δ=8.51 ppm (s, 1H, C*H*); ^**13**^ **C NMR** (DMSO‐*d*_6_, 25 °C): δ=135.0 ppm (N4‐*C*); **IR** (ATR, cm^−1^): $\tilde{\nu }$ =3160 (m), 2889 (s), 2791 (s), 1864 (w), 1687 (w), 1643 (w), 1597 (w), 1468 (s), 1433 (s), 1399 (vs), 1379 (s), 1333 (m), 1233 (vs), 1182 (s), 1147 (m), 1098 (s), 1046 (w), 967 (s), 824 (s), 717 (vs), 666 (s), 485 (m), 421 (w); **DTA** (5 °C min^−1^): 159 (T_exo_), 203 (T_exo_); **elemental analysis** calcd [%] for CH_5_N_5_O_2_ (119.08): C 10.09, H 4.23, N 58.81; found: C 10.26, H 3.86, N 58.01; **BAM drophammer**: 6 J, **BAM friction tester**: 240 N, **ESD**: >1500 mJ.

**Hydrazinium 1‐oxido‐5*H*‐tetrazolate** (**7**): Compound **1** (500 mg, 5.81 mmol) was dissolved in ethanol (10 mL) and hydrazinium hydrate (100%, 291 mg, 5.81 mmol) was added. After stirring for 5 minutes at 50 °C, the precipitate was filtered off, washed with diethyl ether and dried in air. Hydrazinium 1‐oxido‐5*H*‐tetrazolate (**7**) was obtained as a white powder in quantitative yield (685 mg, 5.80 mmol, 99%). Crystals suitable for single crystal X‐Ray measurements were obtained by recrystallization from water.

^**1**^ **H NMR** (DMSO‐*d*_6_, 25 °C): δ=8.52 ppm (s, 1H, C*H*); ^**13**^ **C NMR** (DMSO‐*d*_6_, 25 °C): δ=135.1 ppm (N4‐*C*); **IR** (ATR, cm^−1^): $\tilde{\nu }$ =3297 (m), 3139 (s), 2754 (s), 2628 (s), 1587 (s), 1532 (s), 1455 (m), 1400 (s), 1379 (s), 1336 (w), 1286 (w), 1267 (w), 1238 (s), 1217 (s), 1185 (m), 1134 (s), 1106 (s), 1077 (s), 1042 (m), 957 (vs), 832 (m), 779 (w), 760 (vw), 732 (s), 717 (s), 667 (m), 483 (m), 417 (m), 404 (m); **DTA** (5 °C min^−1^): 213 °C (T_exo_); **elemental analysis** calcd [%] for CH_6_N_6_O (118.10): C 10.17, H 5.12, N 71.16; found: C 10.11, H 5.25, N 71.33; **BAM drophammer**: 26 J, **BAM friction tester**: >360 N, **ESD**: 740 mJ.

**Bis(1‐hydroxytetrazol‐5‐yl)triazene monohydrate** (**8**): Aqueous sodium nitrite (85.3 mg, 1.24 mmol, 5 mL) was added to a solution of 5‐amino‐1‐hydroxytetrazole (250 mg, 2.47 mmol) in hydrochloric acid (2 m, 5 mL) and cooled to 0–5 °C. After stirring for 30 minutes, the pH of the solution was adjusted to pH≥10 by adding sodium hydroxide solution (32%) and stirred at 35 °C for another 30 minutes. The pH of the solution was then adjusted to pH≤2 using hydrochloric acid (37%) and extracted into ethyl acetate (4 x 50 mL). After slowly evaporating the solvent in air, bis(1‐hydroxytetrazol‐5‐yl)triazene monohydrate (**8**) was obtained as a brown crystalline solid. Due to the immediate onset of slow decomposition of **8**, elemental analysis and IR spectroscopic analysis were not performed. Sensitivity measurements were performed immediately after synthesis was complete without determining the purity of the sample.

^**13**^ **C NMR** (DMSO‐d_6_, 25 °C): δ=150.0 ppm; **DTA** (5 °C min^−1^): 100 °C (T_exo_); **BAM drophammer**: <1 J, **BAM friction tester**: 4 N.

### General procedure for the synthesis of bis(1‐oxidotetrazol‐5‐yl)triazenide salts

Stoichiometric equivalents of hydroxides, carbonates or free bases (**14**), dissolved in methanol/water were added to a solution of **8** in ethyl acetate. The resulting solution was stirred for 5 minutes or until precipitation was complete. The precipitate was filtered off and recrystallized from water.

**Trilithium bis(1‐oxidotetrazol‐5‐yl)triazenide hexahydrate** (**9**): Compound **9** was obtained as brown needles in good yield (324 mg, 0.96 mmol, 77%).

^**13**^ **C NMR** (D_2_O, 25 °C): δ=153.0 ppm; **IR** (ATR, cm^−1^): $\tilde{\nu }$ =3338 (s), 3085 (s), 1650 (m), 1579 (m), 1544 (s), 1518 (s), 1455 (m), 1435 (m), 1354 (s), 1325 (s), 1251 (s), 1243 (s), 1227 (s), 1176 (s), 1152 (s), 1125 (m), 1035 (m), 1011 (m), 963 (m), 826 (s), 753 (s), 732 (s), 649 (vs), 544 (vs), 489 (vs), 481 (vs), 465 (s), 457 (vs), 419(vs), 410 (vs); **DTA** (5 °C min^−1^): 311 °C (T_exo_); **elemental analysis** calcd [%] for C_2_H_12_Li_3_N_11_O_8_ (339.01): C 7.09, H 3.57, N 45.45; found: C 7.38, H 3.59, N 45.24; **BAM drophammer**: >40 J; **BAM friction tester**: >360 N; **ESD**:≥1500 mJ.

**Trisodium bis(1‐oxidotetrazol‐5‐yl)triazenide hexahydrate** (**10**): Compound **10** was obtained as yellow‐brown needles in moderate yield (283 mg, 0.73 mmol, 59%).

^**13**^ **C NMR** (D_2_O, 25 °C): δ=153.1 ppm; **IR** (ATR, cm^−1^): $\tilde{\nu }$ =3334 (m), 1664 (m), 1639 (m), 1518 (m), 1497 (m), 1444 (m), 1426 (m), 1357 (s), 1302 (s), 1256 (s), 1242 (s), 1210 (s), 1168 (m), 1147 (m), 1114 (m), 955 (m), 817 (m), 753 (m), 735 (m), 653 (m), 645 (m), 582 (s), 561 (vs), 561 (vs), 512 (vs), 491 (vs), 412 (s); **DTA** (5 °C min^−1^): 338 °C (T_exo_); **elemental analysis** calcd. [%] for C_2_H_12_N_11_Na_3_O_8_ (387.16): C 6.20, H 3.12, N 39.80; found: C 6.19, H 2.83, N 39.02; **BAM drophammer**: >40 J; **BAM friction tester**: >360 N; **ESD**: 1060 mJ.

**Tripotassium bis(1‐oxidotetrazol‐5‐yl)triazenide trihydrate** (**11**): Compound **11** was obtained as yellow needles in moderate yield (248 mg, 0.65 mmol, 53%).

^**13**^ **C NMR** (D_2_O, 25 °C): δ=153.2 ppm; **IR** (ATR, cm^−1^): $\tilde{\nu }$ =3489 (m), 3478 (m), 3262 (m), 3230 (m), 3084 (m), 1688 (w), 1654 (w), 1521 (m), 1505 (s), 1444 (m), 1419 (m), 1320 (vs), 1254 (s), 1238 (s), 1224 (s), 1213 (s), 1159 (m), 1134 (m), 1023 (w), 999 (w), 952 (w), 808 (s), 747 (m), 734 (s), 716 (m), 678 (m), 620 (s), 561 (s), 480 (m), 458 (s); **DTA** (5 °C min^−1^): 292 °C (T_exo_); **elemental analysis** calcd [%] for C_2_H_6_K_3_N_11_O_5_ (381.44): C 6.30, H 1.59, N 40.39; found: C 6.32, H 1.46, N 40.43; **BAM drophammer**: >40 J; **BAM friction tester**: >360 N; **ESD**: >1500 mJ.

**Trirubidium bis(1‐oxidotetrazol‐5‐yl)triazenide trihydrate** (**12**): Compound **12** was obtained as yellow‐brown needles in moderate yield (392 mg, 0.75 mmol, 61%).

^**13**^ **C NMR** (D_2_O, 25 °C): δ=153.2 ppm; **IR** (ATR, cm^−1^): $\tilde{\nu }$ =3486 (m), 3158 (s), 2159 (w), 1668 (w), 1562 (vw), 1516 (m), 1498 (s), 1428 (m), 1409 (m), 1309 (vs), 1249 (s), 1227 (vs), 1208 (s), 1163 (m), 1146 (s), 1027 (m), 1002 (w), 952 (w), 811 (s), 744 (s), 733 (s), 675 (m), 667 (m), 624 (s), 595 (s), 572 (s), 518 (s), 501 (s), 492 (s), 435 (m); **DTA** (5 °C min^−1^): 292 °C (T_exo_); **elemental analysis** calcd [%] for C_2_H_6_N_11_O_5_Rb_3_ (520.55): C 4.61, H 1.16, N 29.60; found: C 4.75, H 1.04, N 29.53; **BAM drophammer**: >40 J; **BAM friction tester**: 288 N; **ESD**:≥750 mJ.

**Triguanidinium bis(1‐oxidotetrazol‐5‐yl)triazenide** (**13**): Compound **13** was obtained as yellow plates in good yield (328 mg, 0.84 mmol, 68%).

^**13**^ **C NMR** (D_2_O, 25 °C): δ=157.9, 153.0 ppm; **IR** (ATR, cm^−1^): $\tilde{\nu }$ =3046 (vw), 3349 (vw), 3404 (w), 2815 (w), 1644 (w), 1519 (vw), 1490 (w), 1434 (vw), 1412 (m), 1340 (vw), 1299 (w), 1242 (w), 1229 (w), 1213 (w), 1158 (w), 1140 (w), 1121 (w), 999 (vw), 954 (vw), 880 (vw), 812 (w), 771 (w), 738 (w), 721 (w), 680 (w), 654 (w), 589 (w), 554 (w), 526 (w), 504 (w), 491 (w), 430 (w), 405 (w); **DTA** (5 °C min^−1^): 222 °C (T_exo_); **elemental analysis** calcd [%] for C_5_H_18_N_20_O_2_ (390.24): C 15.39, H 4.65, N 71.77; found: C 15.33, H 4.65, N 70.31; **BAM drophammer**: >40 J; **BAM friction tester**: >360 N; **ESD**: >1500 mJ.

**Tri(hydroxylammonium) bis(1‐oxidotetrazol‐5‐yl)triazenide** (**14**): Compound **14** was obtained as a pale yellow solid in moderate yield (228 mg, 0.73 mmol, 59%).

**IR** (ATR, cm^−1^): $\tilde{\nu }$ =3597 (m), 3511 (w), 3157 (w), 3059 (m), 3048 (m), 2858 (m), 2724 (s), 2445 (s), 1606 (s), 1558 (m), 1519 (m), 1497 (m), 1472 (s), 1460 (s), 1440 (s), 1415 (s), 1338 (w), 1286 (m), 1263 (s), 1245 (s), 1234 (s), 1199 (vs), 1146 (s), 1126 (s), 1029 (m), 1016 (m), 1004 (m), 991 (m), 933 (s), 886 (m), 810 (s), 742 (m), 727 (m), 710 (m), 692 (m), 668 (m), 645 (s), 576 (m), 483 (m), 455 (m), 440 (s), 426 (m), 403 (s); **DTA** (5 °C min^−1^): 148 °C (T_exo_); **elemental analysis** calcd [%] for C_2_H_12_N_14_O_5_ (312.21): C 7.69, H 3.87, N 62.81; found: C 8.05, H 3.64, N 61.28; **BAM drophammer**: 4 J; **BAM friction tester**: 128 N; **ESD**:≥750 mJ.

**Ammonium copper(II) bis(1‐oxidotetrazol‐5‐yl)triazenide** (**15**): Aqueous sodium nitrite (85.3 mg, 1.24 mmol, 5 mL) was added to a solution of 5‐amino‐1‐hydroxytetrazole (250 mg, 2.47 mmol) in hydrochloric acid (2 m, 5 mL) and cooled to 0–5 °C. After stirring for 30 minutes, the pH of the solution was adjusted to pH≥10 by adding aqueous sodium hydroxide solution (32%), and stirred at 35 °C for another 30 minutes. Copper sulfate pentahydrate (926.4 mg, 3.71 mmol) dissolved in water (5 mL) was added to the reaction solution, and after stirring for 5 minutes, the precipitate was filtered off and washed with water. Recrystallization from aqueous ammonia resulted in compound **15** as green crystalline plates in moderate yield (205 mg, 0.52 mmol, 42%).

**IR** (ATR, cm^−1^): $\tilde{\nu }$ =3323 (m), 3259 (m), 3176 (m), 1609 (m), 1536 (m), 1466 (m), 1433 (w), 1374 (vs), 1319 (s), 1298 (s), 1250 (vs), 1237 (vs), 1219 (vs), 1122 (m), 1052 (m), 1005 (m), 976 (m), 833 (m), 750 (s), 715 (s), 679 (s), 659 (s), 608 (s), 574 (s), 536 (s), 499 (s), 476 (s), 457 (s), 445 (s), 433 (s); DTA (5 °C min^−1^): 212 °C (T_exo_); **elemental analysis** calcd [%] for C_2_H_10_CuN_12_O_5_ (345.73): C 6.95, H 2.92, N 48.62; found: C 6.68, H 2.98, N 48.63; **BAM drophammer**: 11 J; **BAM friction tester**: 288 N; **ESD**: 380 mJ.

## Conflict of interest

The authors declare no conflict of interest.

## Supporting information

As a service to our authors and readers, this journal provides supporting information supplied by the authors. Such materials are peer reviewed and may be re‐organized for online delivery, but are not copy‐edited or typeset. Technical support issues arising from supporting information (other than missing files) should be addressed to the authors.

Supporting Information
