# Supplementary material for: Selective Synthesis and Characterization of the Highly Energetic Materials 1‐Hydroxy‐5H‐tetrazole (CHN4O), its Anion 1‐Oxido‐5H‐tetrazolate (CN4O−) and Bis(1‐hydroxytetrazol‐5‐yl)triazene
Source: Chem Asian J. 2021 Sep 2;16(19):3001–12. doi: 10.1002/asia.202100714 (PMC8518496; doi:10.1002/asia.202100714)
Supplement: Supplementary file 1 — Supporting Information [file ASIA-16-3001-s001.pdf]

CHEMISTRY
AN ASIAN JOURNAL

Supporting Information

Selective Synthesis and Characterization of the Highly Energetic Materials 1-Hydroxy-5*H*-tetrazole (CHN_4O), its Anion 1-Oxido-5*H*-tetrazolate (CN_4O^-) and Bis(1-hydroxytetrazol-5-yl)triazene

Thomas M. Klapötke,* Moritz Kofen, Laszlo Schmidt, Jörg Stierstorfer, and Maximilian H. H. Wurzenberger© 2021 The Authors. Chemistry - An Asian Journal published by Wiley-VCH GmbH. This is an open access article under the terms of the Creative Commons Attribution License, which permits use, distribution and reproduction in any medium, provided the original work is properly cited.

Table of Contents

1. Compounds Overview

2. Single Crystal X-Ray Diffraction

3 Crystal structures of **9**, **10**, **12** and **13**

4. Computations & Hirshfeld Surfaces

5. NMR spectroscopy of **1**–**3**, **5**–**13**

6. IR Spectroscopy of **1**–**15**

7. General Methods

8. References

1. Overview of compounds

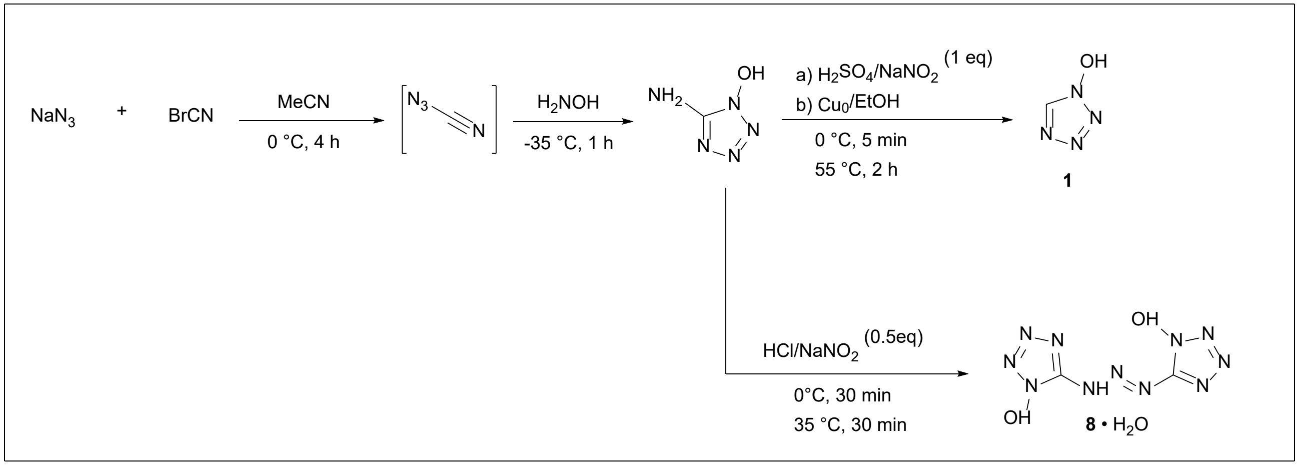

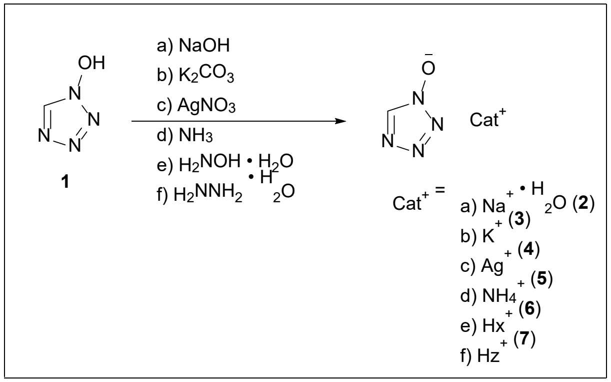

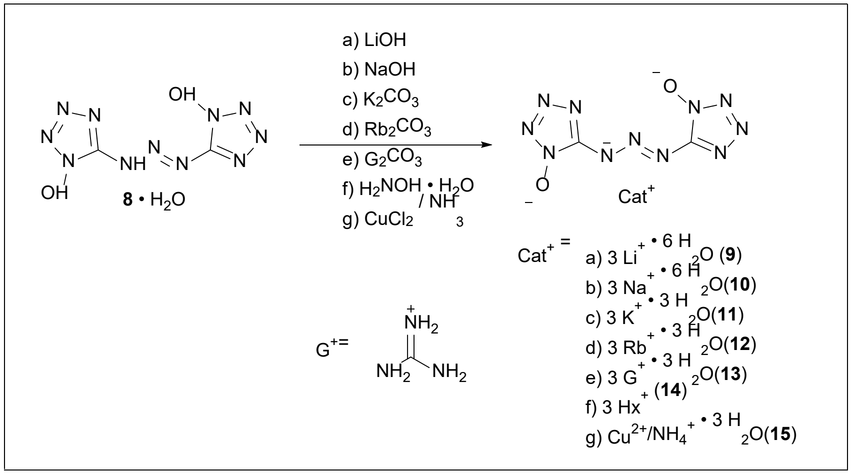

2. Single Crystal X-Ray Diffraction

For all crystalline compounds, an Oxford Xcalibur3 diffractometer with a CCD area detector or Bruker D8 Venture TXS diffractometer equipped with a multilayer monochromator, a Photon 2 detector and a rotating-anode generator were employed for data collection using Mo-K_{α} radiation (λ = 0.7107 Å). On the Oxford device, data collection and reduction were carried out using the CrysAlisPRO software.[S1] On the Bruker diffractometer, the data were collected with the Bruker Instrument Service v3.0.21, the data reduction was performed using the SAINT V8.18C software (Bruker AXS Inc., 2011). The structures were solved by direct methods (SIR-92,[S2] SIR-97,[S3,S4] SHELXS-97[S5,S6] or SHELXT[S7]) and refined by full-matrix least-squares on *F*2 (SHELXL[S5,S6]) and finally checked using the PLATON software[S8] integrated in the WinGX[S7,S9] or Olex2[S8] software suite. The non-hydrogen atoms were refined anisotropically and the hydrogen atoms were located and freely refined. The absorptions were corrected by a SCALE3 ABSPACK or SADABS Bruker APEX3 multi-scan method.[S11,S12] All DIAMOND2 plots are shown with thermal ellipsoids at the 50% probability level and hydrogen atoms are shown as small spheres of arbitrary radius.

Table S1. Crystallographic data and structure refinement details for compounds **1** and **2a/b.**

	*HTO (**1**)*	*NaTO_P1 (**2a**)*	*NaTO_P21 (**2b**)*
Formula	C H2 N4 O	C H3 N4 Na O2	C H3 N4 Na O2
FW [g mol^{-1}]	86.07	126.06	126.06
Crystal system	Monoclinic	Triclinic	Orthorhombic
Space group	$P2_1/n$ (No. 14)	$P1$ (No. 1)	$P2_12_12_1$ (No. 19)
Color / Habit	White needle	Colorless block	Colorless rod
Size [mm]	0.08 x 0.21 x 0.21	0.04 x 0.04 x 0.09	0.02 x 0.02 x 0.10
a [Å]	5.1610(4)	3.5162(3)	3.5145(3)
b [Å]	7.6125(4)	4.9474(5)	8.8624(7)
c [Å]	8.7911(5)	6.8684(6)	14.6572(12)
α [°]	90	76.042(3)	90
β [°]	98.147(6)	78.148(3)	90
γ [°]	90	80.657(3)	90
V [Å^3]	341.90(4)	112.680(18)	456.53(6)
Z	4	1	4
$\rho_{calc.}$ [g cm^{-3}]	1.672	1.858	1.834
μ [mm^{-1}]	0.145	0.242	0.239
F(000)	176	64	256
$\lambda_{MoK\alpha}$ [Å]	0.71073	0.71073	0.71073
T [K]	123	108	112
θ Min-Max [°]	3.6, 26.4	3.1, 30.5	2.8, 26.4
Dataset	-6: 6 ; -9: 9 ; -10: 10	-5: 5 ; -7: 7 ; -9: 9	-4: 4 ; -11: 11 ; -18: 18
Reflections collected	2466	3134	6490
Independent refl.	696	1325	924
R_{int}	0.023	0.032	0.030
Observed reflections	585	1298	890
Parameters	63	82	82
R_1 (obs)[a]	0.0320	0.0239	0.0200
wR_2 (all data)[b]	0.0818	0.0625	0.0465
S [c]	1.06	1.07	1.15
Resd. dens [e Å^{-3}]	-0.16, 0.57	-0.19, 0.20	-0.19, 0.15
Device type	Oxford Xcalibur3	Bruker D8 Venture TXS	Bruker D8 Venture TXS
Solution	SIR-92	SIR-92	SIR-92
Refinement	SHELXL-2018	SHELXL-2018	SHELXL-2018
Absorption correction	multi-scan	multi-scan	multi-scan

[a]$R_1 = \Sigma||F_0|-|F_c||/\Sigma|F_0|$; [b]$wR_2 = [\Sigma[w(F_0^2-F_c^2)^2]/\Sigma[w(F_0)^2]]^{1/2}$; $w = [\sigma c^2(F_0^2)+(xP)^2+yP]^{-1}$ and $P=(F_0^2+2F_c^2)/3$; [c]$S = \{\Sigma[w(F_o^2-F_c^2)^2]/(n-p)\}^{1/2}$ (n = number of reflections; p = total number of parameters).

Table S2. Crystallographic data and structure refinement details for compounds **3**, **4**, and **5**.

	*KTO (**3**)*	*AgTO (**4**)*	*NH4TO (**5**)*
Formula	C H K N4 O	C H Ag N4 O	C H N4 O, H4 N
FW [g mol^{-1}]	124.16	192.93	103.10
Crystal system	Orthorhombic	Orthorhombic	Orthorhombic
Space group	$Pna2_1$ (No. 33)	$Pna2_1$ (No. 33)	$Pna2_1$ (No. 33)
Color / Habit	Colorless block	Colorless rod	Colorless block
Size [mm]	0.21 x 0.22 x 0.42	0.02 x 0.03 x 0.13	0.45 x 0.52 x 0.82
a [Å]	7.2219(4)	10.2602(5)	7.8330(7)
b [Å]	4.7357(2)	10.2774(5)	4.5703(4)
c [Å]	12.5270(6)	3.4349(2)	13.2268(14)
α [°]	90	90	90
β [°]	90	90	90
γ [°]	90	90	90
V [Å^3]	428.43(4)	362.20(3)	473.51(8)
Z	4	4	4
$\rho_{calc.}$ [g cm^{-3}]	1.925	3.538	1.446
μ [mm^{-1}]	1.095	5.393	0.123
F(000)	248	360	216
$\lambda_{MoK\alpha}$ [Å]	0.71073	0.71073	0.71073
T [K]	121	173	99
θ Min-Max [°]	4.6, 26.4	4.0, 29.6	3.1, 32.5
Dataset	-8: 9 ; -5: 5 ; -15: 15	-14: 14 ; -14: 14 ; -4: 4	-11: 11 ; -6: 6 ; -19: 15
Reflections collected	2778	8144	4905
Independent refl.	863	1004	1419
R_{int}	0.024	0.056	0.022
Observed reflections	843	972	1283
Parameters	69	65	81
R_1 (obs)[a]	0.0183	0.0203	0.0321
wR_2 (all data)[b]	0.0458	0.0478	0.0840
S [c]	1.12	1.05	1.08
Resd. dens [e Å^{-3}]	-0.19, 0.21	-0.60, 0.57	-0.13, 0.19
Device type	Oxford Xcalibur3	Oxford Xcalibur3	Oxford Xcalibur3
Solution	SIR-92	SHELXT	SHELXT
Refinement	SHELXL-2018	SHELXL-2018	SHELXL-2018
Absorption correction	multi-scan	multi-scan	multi-scan

[a]$R_1 = \Sigma||F_0|-|F_c||/\Sigma|F_0|$; [b]$wR_2 = [\Sigma[w(F_0^2-F_c^2)^2]/\Sigma[w(F_0)^2]]^{1/2}$; $w = [\sigma c^2(F_0^2)+(xP)^2+yP]^{-1}$ and $P=(F_0^2+2F_c^2)/3$; [c]$S = \{\Sigma[w(F_o^2-F_c^2)^2]/(n-p)\}^{1/2}$ (n = number of reflections; p = total number of parameters).

Table S3. Crystallographic data and structure refinement details for compounds **6**, **7**, and **8**.

	*HxTO (**6**)*	*HzTO (**7**)*	*H3(TO)2T (**8**)*
Formula	C H N4 O, H4 N O	C H N4 O, H5 N2	C2 H3 N11 O2, H2 O
FW [g mol^{-1}]	119.10	118.12	231.17
Crystal system	Triclinic	Monoclinic	Monoclinic
Space group	*P*1 (No. 1)	*Pc* (No. 7)	$P2_1/n$ (No. 14)
Color / Habit	Colorless platelet	Colorless rod	Orange block
Size [mm]	0.03 x 0.08 x 0.10	0.06 x 0.17 x 0.57	0.09 x 0.20 x 0.37
a [Å]	3.6986(5)	4.8164(6)	12.0542(5)
b [Å]	4.8897(8)	7.0591(10)	5.7942(2)
c [Å]	6.5638(11)	7.0853(11)	12.1983(4)
α [°]	88.745(6)	90	90
β [°]	86.322(6)	98.485(15)	103.718(4)
γ [°]	79.164(6)	90	90
V [Å^3]	116.34(3)	238.26(6)	827.68(5)
Z	1	2	4
$\rho_{calc.}$ [g cm^{-3}]	1.700	1.646	1.855
μ [mm^{-1}]	0.153	0.139	0.164
F(000)	62	124	472
$\lambda_{MoK\alpha}$ [Å]	0.71073	0.71073	0.71073
T [K]	173	102	107
θ Min-Max [°]	4.2, 27.5	2.9, 32.3	3.4, 26.4
Dataset	-4: 4 ; -6: 6 ; -8: 7	-7: 6 ; -10: 10 ; -10: 6	-15: 15 ; -7: 6 ; -13: 15
Reflections collected	1707	2003	5264
Independent refl.	871	1077	1688
R_{int}	0.035	0.037	0.027
Observed reflections	848	851	1406
Parameters	93	98	165
R_1 (obs)[a]	0.0277	0.0461	0.0340
wR_2 (all data)[b]	0.0703	0.0982	0.0874
S [c]	1.10	1.05	1.06
Resd. dens [e Å^{-3}]	-0.16, 0.20	-0.28, 0.28	-0.17, 0.26
Device type	Bruker D8 Venture TXS	Oxford Xcalibur3	Oxford Xcalibur3
Solution	SHELXT	SHELXT	SIR-92
Refinement	SHELXL-2018	SHELXL-2018	SHELXL-2018
Absorption correction	multi-scan	multi-scan	multi-scan

[a] $R_1 = \Sigma||F_0|-|F_c||/\Sigma|F_0|$; [b] $wR_2 = [\Sigma[w(F_0^2-F_c^2)^2]/\Sigma[w(F_0)^2]]^{1/2}$; $w = [\sigma c^2(F_0^2)+(xP)^2+yP]^{-1}$ and $P=(F_0^2+2F_c^2)/3$; [c] $S = \{\Sigma[w(F_o^2-F_c^2)^2]/(n-p)\}^{1/2}$ (n = number of reflections; p = total number of parameters).

Table S4. Crystallographic data and structure refinement details for compounds **9**, **10**, and **11**.

	*Li3(TO)2T (**9**)*	*Na3(TO)2T (**10**)*	*K3(TO)2T (**11**)*
Formula	C2 H12 Li3 N11 O8	C2 H12 N11 Na3 O8	C2 H6 K3 N11 O5
FW [g mol^{-1}]	339.05	387.20	381.48
Crystal system	Triclinic	Triclinic	Orthorhombic
Space group	*P*-1 (No. 2)	*P*-1 (No. 2)	$P2_12_12_1$ (No. 19)
Color / Habit	Yellow block	Yellow platelet	Yellow needle
Size [mm]	0.14 x 0.29 x 0.42	0.07 x 0.16 x 0.22	0.10 x 0.13 x 0.55
a [Å]	6.9812(9)	7.1050(12)	6.6032(3)
b [Å]	8.7124(9)	8.1609(10)	11.3834(4)
c [Å]	11.2511(13)	13.507(2)	16.6430(7)
α [°]	81.165(9)	95.747(11)	90
β [°]	84.575(10)	93.543(13)	90
γ [°]	76.353(10)	109.254(14)	90
V [Å^3]	655.90(14)	731.9(2)	1251.00(9)
Z	2	2	4
$\rho_{calc.}$ [g cm^{-3}]	1.717	1.757	2.026
μ [mm^{-1}]	0.156	0.233	1.137
F(000)	348	396	768
$\lambda_{MoK\alpha}$ [Å]	0.71073	0.71073	0.71073
T [K]	124	125	122
θ Min-Max [°]	3.2, 26.4	3.3, 26.4	3.3, 26.4
Dataset	-8: 8 ; -10: 10 ; -14: 9	-5: 8 ; -10: 9 ; -14: 16	-8: 8 ; -12: 14 ; -19: 20
Reflections collected	4572	4006	9475
Independent refl.	2677	2954	2557
R_{int}	0.053	0.031	0.043
Observed reflections	1609	2100	2390
Parameters	259	265	191
R_1 (obs)[a]	0.0582	0.0538	0.0256
wR_2 (all data)[b]	0.1226	0.1128	0.0539
S [c]	0.99	1.04	1.04
Resd. dens [e Å^{-3}]	-0.33, 0.47	-0.34, 0.35	-0.22, 0.27
Device type	Oxford Xcalibur3	Oxford Xcalibur3	Oxford Xcalibur3
Solution	SIR-92	SIR-92	SIR-92
Refinement	SHELXL-2018	SHELXL-2018	SHELXL-2018
Absorption correction	multi-scan	multi-scan	multi-scan

[a]$R_1 = \Sigma||F_0|-|F_c||/\Sigma|F_0|$; [b]$wR_2 = [\Sigma[w(F_0^2-F_c^2)^2]/\Sigma[w(F_0)^2]]^{1/2}$; $w = [\sigma c^2(F_0^2)+(xP)^2+yP]^{-1}$ and $P=(F_0^2+2F_c^2)/3$; [c]$S = \{\Sigma[w(F_o^2-F_c^2)^2]/(n-p)\}^{1/2}$ (n = number of reflections; p = total number of parameters).

Table S5. Crystallographic data and structure refinement details for compounds **12**, **13**, and **15**.

	*Rb3(TO)2T (**12**)*	*Gua3(TO)2T (**13**)*	*Cu(NH$_4$)(TO)2T (**15**)*
Formula	C2 H6 N11 O5 Rb3	C2 N11 O2, 3(C H6 N3), 3(H2 O)	C2 H4 Cu N11 O4, H2 O, H4 N
FW [g mol^{-1}]	520.59	444.44	345.76
Crystal system	Triclinic	Monoclinic	Triclinic
Space group	*P*-1 (No. 2)	$P2_1/c$ (No. 14)	*P*-1 (No. 2)
Color / Habit	Colorless rod	Yellow block	Green plate
Size [mm]	0.02 x 0.02 x 0.10	0.05 x 0.06 x 0.07	0.06 x 0.08 x 0.35
a [Å]	6.6520(2)	6.9605(4)	7.0091(7)
b [Å]	8.5339(3)	14.0622(8)	7.1350(7)
c [Å]	13.0590(5)	19.5249(12)	12.4292(11)
α [°]	98.618(1)	90	104.065(8)
β [°]	99.173(1)	98.069(2)	105.838(8)
γ [°]	112.219(1)	90	94.746(8)
V [Å^3]	659.35(4)	1892.18(19)	572.61(10)
Z	2	4	2
$\rho_{calc.}$ [g cm^{-3}]	2.622	1.560	2.005
μ [mm^{-1}]	11.134	0.133	1.958
F(000)	492	936	350
$\lambda_{MoK\alpha}$ [Å]	0.71073	0.71073	0.71073
T [K]	106	102	133
θ Min-Max [°]	2.6, 26.4	2.9, 26.4	3.5, 27.5
Dataset	-8: 8 ; -10: 10 ; -16: 16	-8: 8 ; -17: 17 ; -24: 24	-9: 9 ; -9: 8 ; -15: 16
Reflections collected	9632	29861	4778
Independent refl.	2686	3866	2618
R_{int}	0.028	0.058	0.065
Observed reflections	2489	3353	1786
Parameters	214	367	221
R_1 (obs)[a]	0.0178	0.0337	0.0605
wR_2 (all data)[b]	0.0408	0.0861	0.1169
S [c]	1.09	1.10	1.01
Resd. dens [e Å^{-3}]	-0.35, 0.46	-0.28, 0.21	-0.80, 0.66
Device type	Bruker D8 Venture TXS	Bruker D8 Venture TXS	Oxford Xcalibur3
Solution	SIR-92	SIR-92	SIR-92
Refinement	SHELXL-2018	SHELXL-2018	SHELXL-2018
Absorption correction	multi-scan	multi-scan	multi-scan

[a]$R_1 = \Sigma||F_0|-|F_c||/\Sigma|F_0|$; [b]$wR_2 = [\Sigma[w(F_0^2-F_c^2)^2]/\Sigma[w(F_0)^2]]^{1/2}$; $w = [\sigma c^2(F_0^2)+(xP)^2+yP]^{-1}$ and $P=(F_0^2+2F_c^2)/3$; [c]$S = \{\Sigma[w(F_o^2-F_c^2)^2]/(n-p)\}^{1/2}$ (n = number of reflections; p = total number of parameters).

3. Crystal structures of 9, 10, 12 and 13

Compound **9** crystallizes in the triclinic space group *P*−1 as a hexahydrate (Figure S1) in the form of yellow plates. The unit cell contains two formula units and **9** has a calculated density of 1.717 g cm^{-3} at 124 K. The anion is nearly planar with a torsion angle between the two 1-oxidotetrazole moieties of 0.7°.

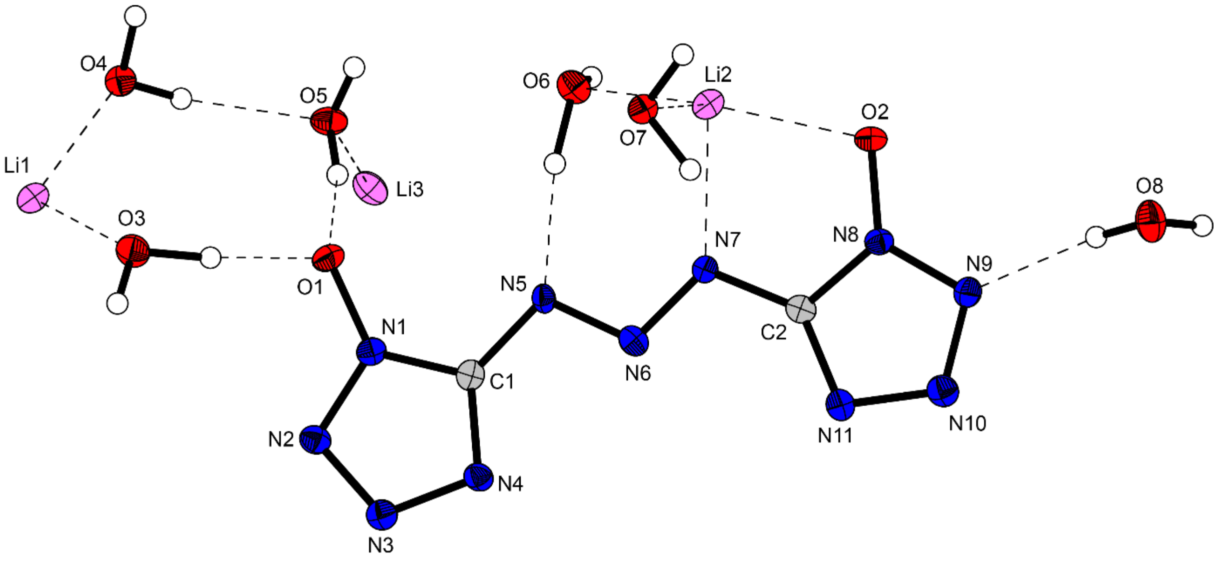

Figure S1. Crystal structure of compound **9**; Selected interatomic distances [Å]: O1–N1 1.337(4), N1–N2 1.354(4), N1–C1 1.341(5), N2–N3 1.319(5), N3–N4 1.357(4), N4–C1 1.328(4), N5–C1 1.378(4), N5–N6 1.310(4), N6–N7 1.312(4), N7–C2 1.366(4), O3–Li1 1.975(6), O4–Li1 2.111(6), O2–Li2 2.164(6), O6–Li2 2.078(6), O7–Li2 2.071(6), O5–Li3 1.958(7), O7–Li3 1.965(6); Angles [°] O1–N1–N2 122.1(3), O1–N1–C1 128.5(3), N2–N1–C1 109.3(3), N1–N2–N3 105.1(3), N2–N3–N4 111.4(3), N3–N4–C1 105.7(3), N6–N5–C1 109.3(2), N5–N6–N7 110.4(3), N6–N7–C2 112.9(2), N7–C2–N11 135.4(3).

Compound **10** crystallizes in the triclinic space group *P*−1 as a hexahydrate (Figure S2) in the form of yellow platelets. The unit cell contains two formula units and **10** has a calculated density of 1.757 g cm^{-3} at 125 K. The anion is slightly twisted with a torsion angle between the two tetrazole rings of 3° (N4–C1–C2–N11).

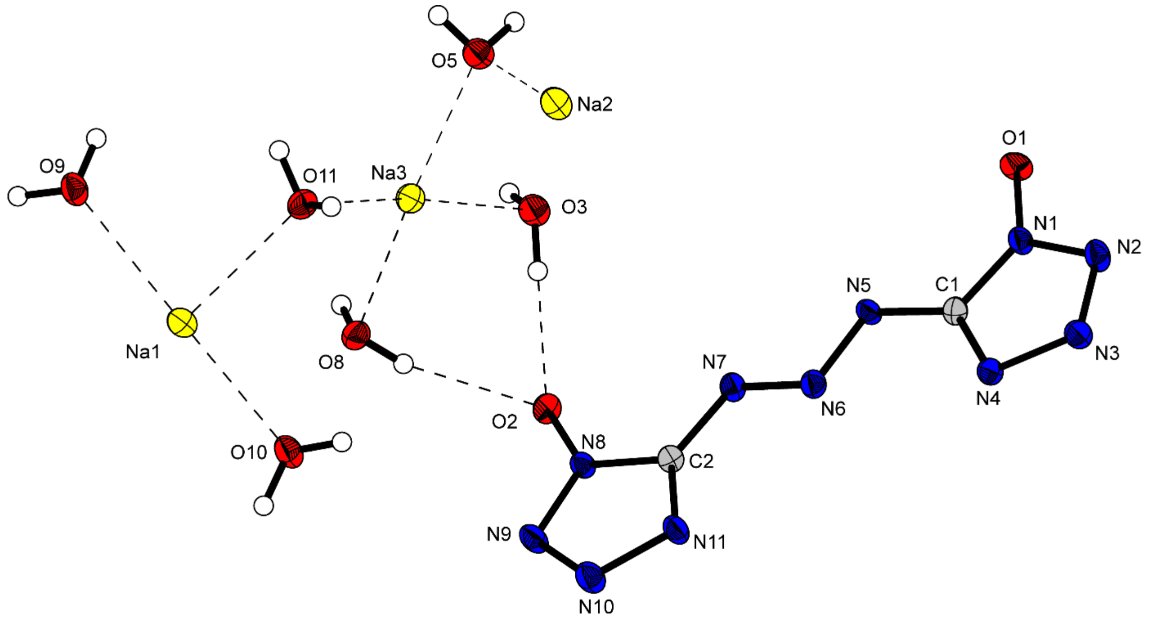

Figure S2. Crystal structure of compound **10**; Selected interatomic distances [Å]: O1–N1 1.323(3), N1–N2 1.350(3), N2–N3 1.316(3), N3–N4 1.371(4), N4–C1 1.337(4), C1–N1 1.347(4), C1–N5 1.382(4), N5–N6 1.325(3), N6–N7 1.304(3), N7–C2 1.377(4), Na1–O9 2.450(3), Na1–O10 2.404(3), Na1–O11 2.366(3), Na2–O5 2.267(3), Na3–O3 2.420(3), Na3–O5 2.407(3), Na3–O8 2.392(3), Na3–O11 2.335(3); Angles [°] O1–N1–N2 122.1(2), O1–N1–C1 128.9(2), N2–N1–C1 109.0(2), N1–N2–N3 106.2(2), N2–N3–N4 110.7(2), N3–N4–C1 105.5(2), N4–C1–N5 130.9(3), N1–C1–N4 108.5(3), N1–C1–N5 120.6(3), N6–N5–C1 110.3(2), N5–N6–N7 110.9(2), N6–N7–C2 113.4(2), N8–C2–N11 107.3(2), N7–C2–N8 118.0(3), N7–C2–N11 134.6(3).

Compound **12** crystallizes as colorless rods in the triclinic space group *P*−1, and contains two formula units in the unit cell. Like compound **11** it is a trihydrate and has a calculated density of 2.622 g cm^{-3} at 106 K (Figure S3). The anion is nearly planar with a small torsion angle between the two 1-oxidotetrazole moieties of 1.8° (O1–N1–N8–O2).

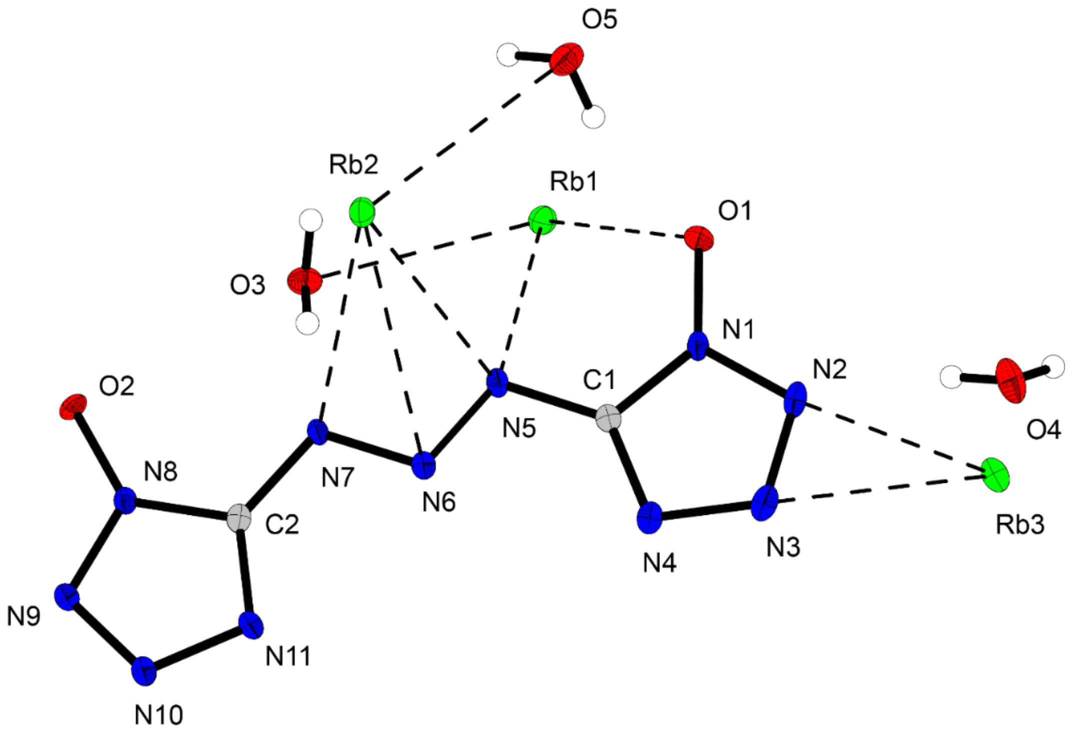

Figure S3. Crystal structure of compound **12**; Selected interatomic distances [Å]: O1–N1 1.333(2), N1–N2 1.348(3), N2–N3 1.313(3), N4–N4 1.350(3), N4–C1 1.341(3), N1–C1 1.350(3), C1–N5 1.381(3), N5–N6 1.309(3), N6–N7 1.316(3), N7–C2 1.380(3), Rb1–O1 2.9309(16), Rb1–O3 2.8886(18), Rb1–N5 3.3111(19), Rb2–N5 3.0573(19), Rb2–N6 3.1407(19), Rb2–N7 3.1339(19), Rb2–O5 3.1863(19), Rb3–N2 3.1668(19), Rb3–N3 3.595(2), Rb3–O4 2.912(2); Angles [°] O1–N1–N2 121.76(18), O1–N1–C1 129.51(19), N2–N1–C1 108.72(19), N3–N1–106.24(18), N2–N3–N4 111.16(18), C1–N4–N3 105.91(19), N6–N5–C1 110.50(19), N5–N6–N7 110.82(18), N6–N7–C2 111.66(19), N4–C1–N1 107.97(19), N4–C1–N5 131.9(2), N1–C1–N5 120.1(2), N11–C2–N7 131.8(2), N8–C2–N7 120.2(2).

Compound **13** (Figure 4) crystallizes in the monoclinic space group $P2_1/c$ as a trihydrate, with a density of 1.560 g cm^{-3} at 102 K. As was observe for compound **10**, the structure of the anion in **13** is twisted with a torsion angle of 21.8° between the 1-oxidotetrazole moieties. In contrast to **10**, in compound **13** this is caused by hydrogen bonding involving the guanidinium cations, bridging between anions in different layers, resulting in the protrusion of O2 out of the plane.

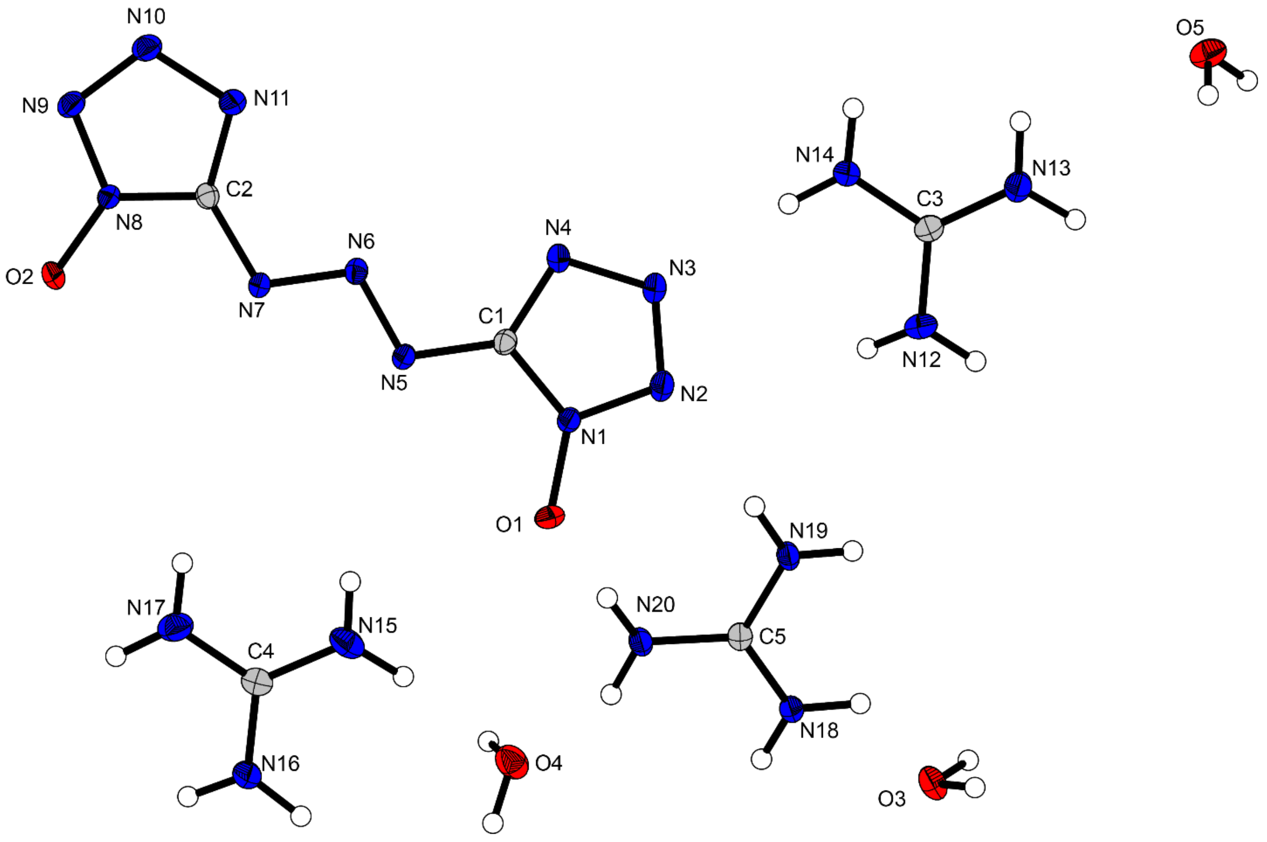

Figure S4. Crystal structure of compound **13**; Selected interatomic distances [Å]: O1–N1 1.3340(15), O2–N8 1.3365(15), N1–N2 1.3433(16), N1–C1 1.3455(17), N2–N3 1.3123(16), N3–N4 1.3545(16), N4–C1 1.3429(18), N5–N6 1.3058(16), N5–C1 1.3771(17), N6–N7 1.3098(15), N7–C2 1.3807(17), N8–N9 1.3372(16), N8–C2 1.3407(16), N9–N10 1.3105(16), N10–N11 1.3565(16), N11–C2 1.3340(18), N12–C3 1.3319(19), N13–C3 1.3203(19), N14–C3 1.3269(19), N15–C4 1.327(2), N16–C4 1.3154(19), N17–C4 1.329(2), N18–C5 1.3186(17), N19–C5 1.3377(18), N20–C5 1.3301(18); Angles [°]: O1–N1–N2 122.06(11), O1–N1–C1 128.87(11), N2–N1–C1 109.07(11), N1–N2–N3 106.39(10), N2–N3–N4 110.79(11), N3–N4–C1 105.94(11), N6–N5–C1 110.82(11), N5–N6–N7 110.96(11), N6–N7–C2 112.34(11), O2–N8–N9 122.93(10), O2–N8–C2 127.33(11), N9–N8–C2 109.74(11), N8–N9–N10 105.69(10), N9–N10–N11 111.11(11), N10–N11–C2 105.69(11), N1–C1–N4 107.81(11), N1–C1–N5 120.89(12), N4–C1–N5 131.28(12), N7–C2–N8 118.63(12), N7–C2–N11 133.56(12), N8–C2–N11 107.77(11).

4. Computations

4.1 Computations

All calculations were carried out using the Gaussian G09 program package.[S13] The enthalpies (H) and free energies (G) were calculated using the complete basis set (CBS) method of Petersson and coworkers in order to obtain very accurate energies. The CBS models use the known asymptotic convergence of pair natural orbital expressions to extrapolate from calculations using a finite basis set to the estimated complete basis set limit. CBS-4 begins with a HF/3-21G(d) geometry optimization; the zero point energy is computed at the same level. It then uses a large basis set

SCF calculation as a base energy, and a MP2/6-31+G calculation with a CBS extrapolation to correct the energy through second order. A MP4(SDQ)/6-31+(d,p) calculation is used to approximate higher order contributions. In this study we applied the modified CBS-4M method (M referring to the use of minimal population localization) which is a re-parametrized version of the original CBS-4 method and also includes some additional empirical corrections. The enthalpies of the gas-phase species M were computed according to the atomization energy method (E1) (Table S6 & 7).[S13–18]

$$\Delta_f H^\circ_{(g,\ M,\ 298)} = H_{(Molecule,\ 298)} - \sum H^\circ_{(Atoms,\ 298)} + \sum \Delta_f H^\circ_{(Atoms,\ 298)} \qquad (E1)$$

Table S6. Literature values for atomic $\Delta H^\circ{}_f{}^{298}$ / kcal mol^{-1}

	$-H^{298}$ [a.u.]	NIST[S19]
H	0.50091	52.1
C	37.786156	171.3
N	54.522462	113.0
O	74.991202	59.6

The gas-phase heat of formations were converted to the solid/liquid state ones (i) for neutrals: by subtracting the vaporization/sublimation enthalpies (calculated using the Trouton rule)[S20,21] (ii) for ionics: using the Jenkins' equations for XY and X_2Y salts[S22,S23] based on the molecular volumes Vm (taken from X-Ray strucutres) in order to calculate the lattice energies (ΔE_L) and enthalpies (ΔH_L). At last, the molar standart enthalpies of formation ($\Delta_f H_M$) were used to calculate the molar solid state energie of formation (ΔU_m) according to equation (E2), Δn being the change of moles of gaseous components. The calculation results are summarized in Table S7.

$$\Delta U_m = \Delta H_m - \Delta n\, R\, T \qquad \text{(E2)}$$

Table S7. CBS-4M results, Gas phase enthalpies of formation, calculated sublimation/vaporization enthalpies and solid-state heat of formation.

Compound	$-H^{298}$ / a.u.	$\Delta_f H°(g)$ / kJ mol^{-1}	ΔE_L, ΔH_L (**2**-**7**, **9**-**15**); $\Delta H°_{sub}$ (**1**, **8**) / kJ mol^{-1}	V_m / nm^3	Δn
**1**	–332.996269	333.4	25.9335		3.5
**1** anion	–332.478900	158.4			
**8**	–829.380882	775.6	12.264676		8
**8** anion	–827.534674	1268.8			
**Li^+**	–7.434217	159.3			
**Na^+**	–161.848174	107.5			
**K^+**	–599.035967	487.7			
**NH_4^+**	–56.796608	635.3			
**NH_4O^+**	–131.863249	686.4			
**$N_2H_5^+$**	–112.030523	773.4			
**Cu^{2+}**	–1639.2574	337.5			
**Guanidinium cation**	–205.453192	409.8			

**2a**		265.8	630.9, 632.1	0.088	3
**2b**		265.8	628.0, 6293	0.090	3
**3**		645.8	597.8, 599.0	0.107	3
**5**		793.7	581.6, 586.5	0.118	5.5
**6**		844.8	581.0, 586.0	0.119	6
**7**		931.8	580.6, 585.5	0.119	6.5
**9**		1428.1	3252.6, 3251.4	0.181	6.5
**10**		1376.3	3072.8, 3071.5	0.219	6.5
**11**		1357.8	3012.3, 3011.0	0.239	6.5
**13**		713.4	2819.5, 2829.4	0.452	20
**14**		796.0	2862.6, 2868.8	0.425	15.5
**15**		532.5	3415.2, 3425.1	0.213	13.5
TNT	–	–	–	−59.346	

5. NMR spectroscopy of **1–3** and **5–13**

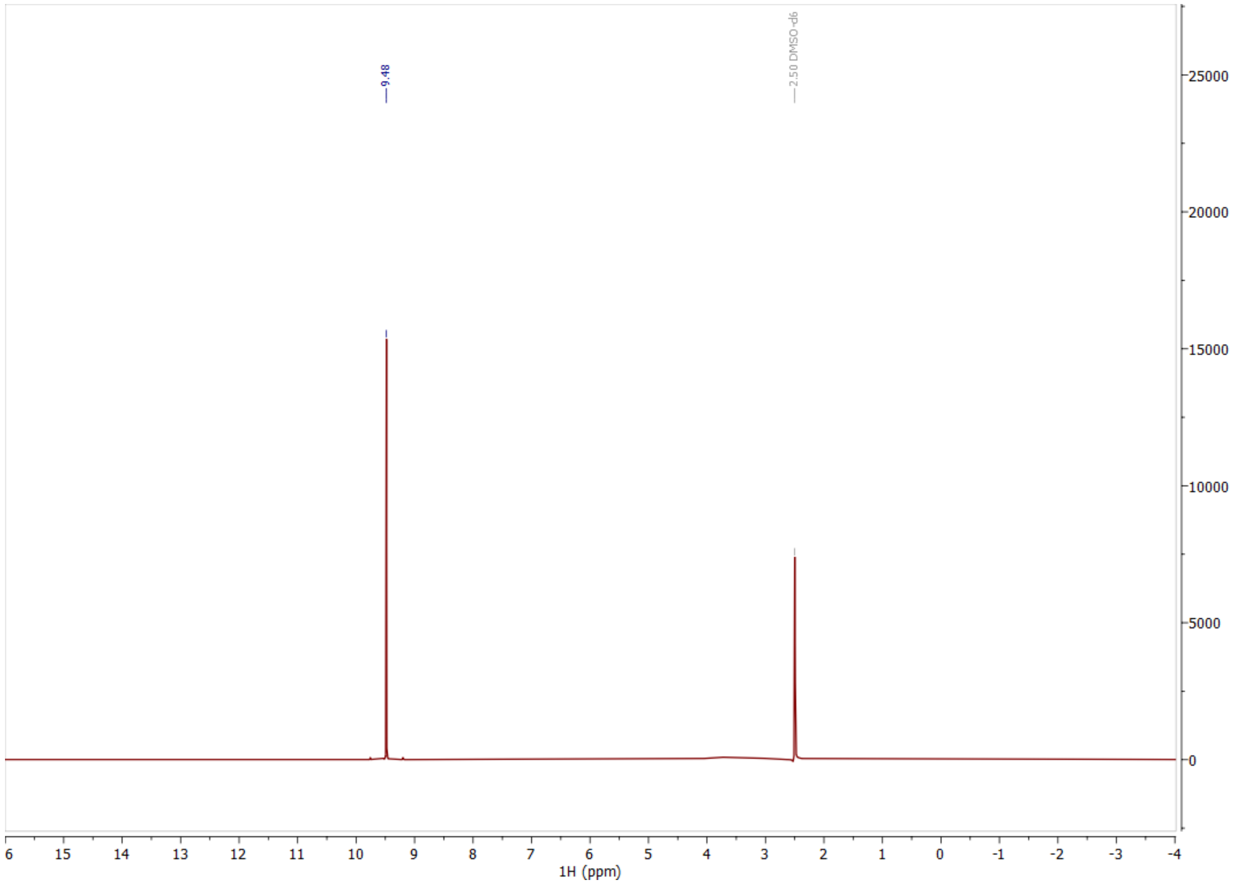

Figure S5. ^{1}H NMR spectrum of 1-hydroxytetrazole (**1**) in DMSO-d_6.

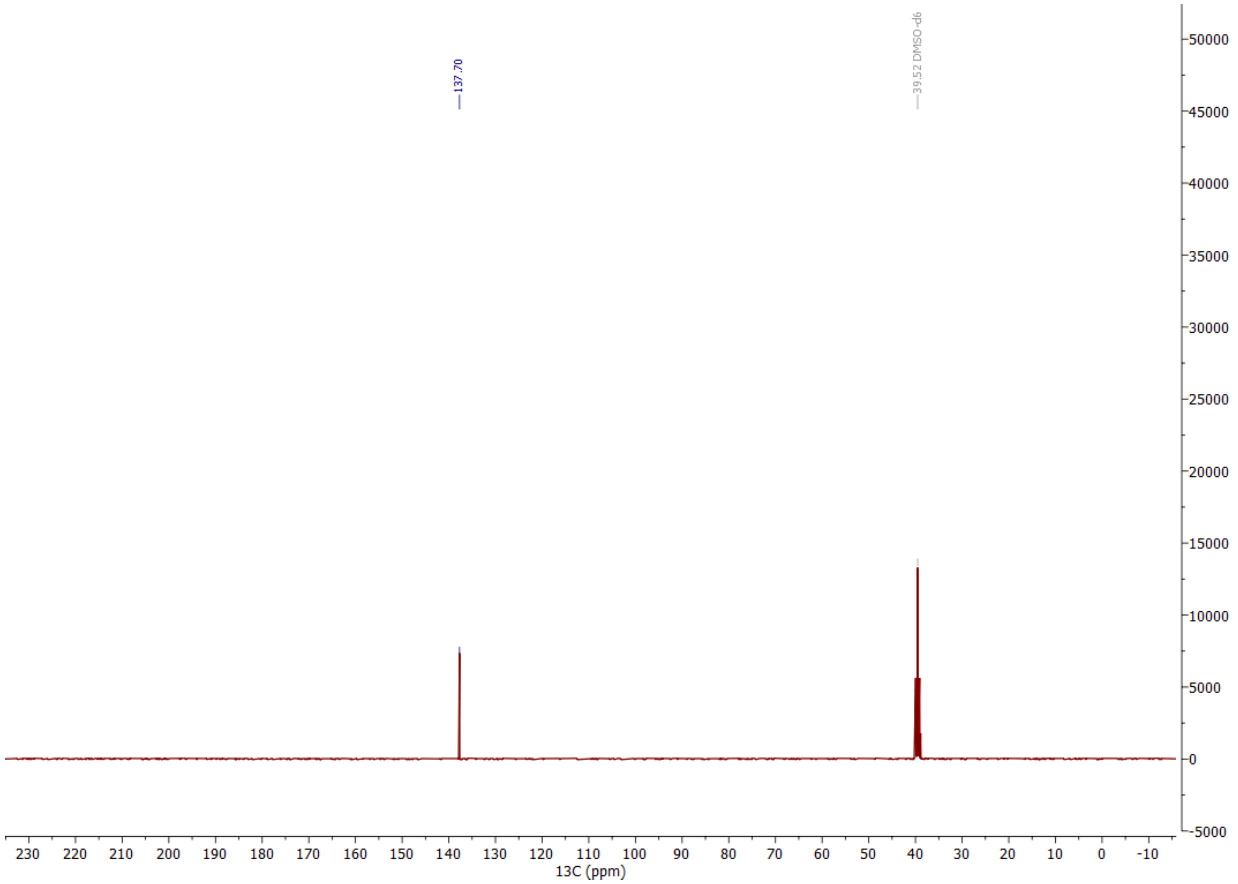

Figure S6. $^{13}C\{^{1}H\}$ NMR spectrum of 1-hydroxytetrazole (**1**) in DMSO-d_6.

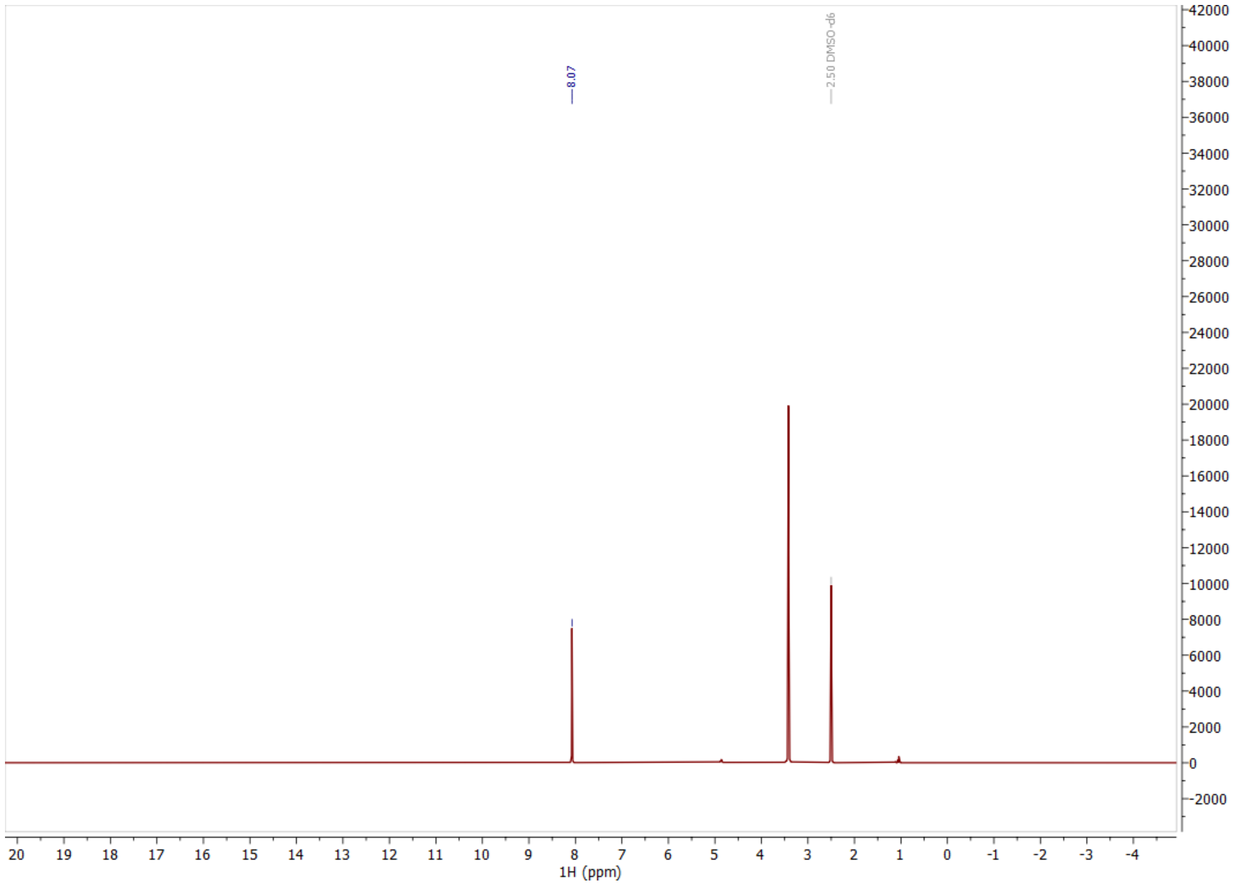

Figure S7. ^{1}H NMR spectrum of sodium 1-oxido-5*H*-tetrazolate (**2**) in DMSO-d_6.

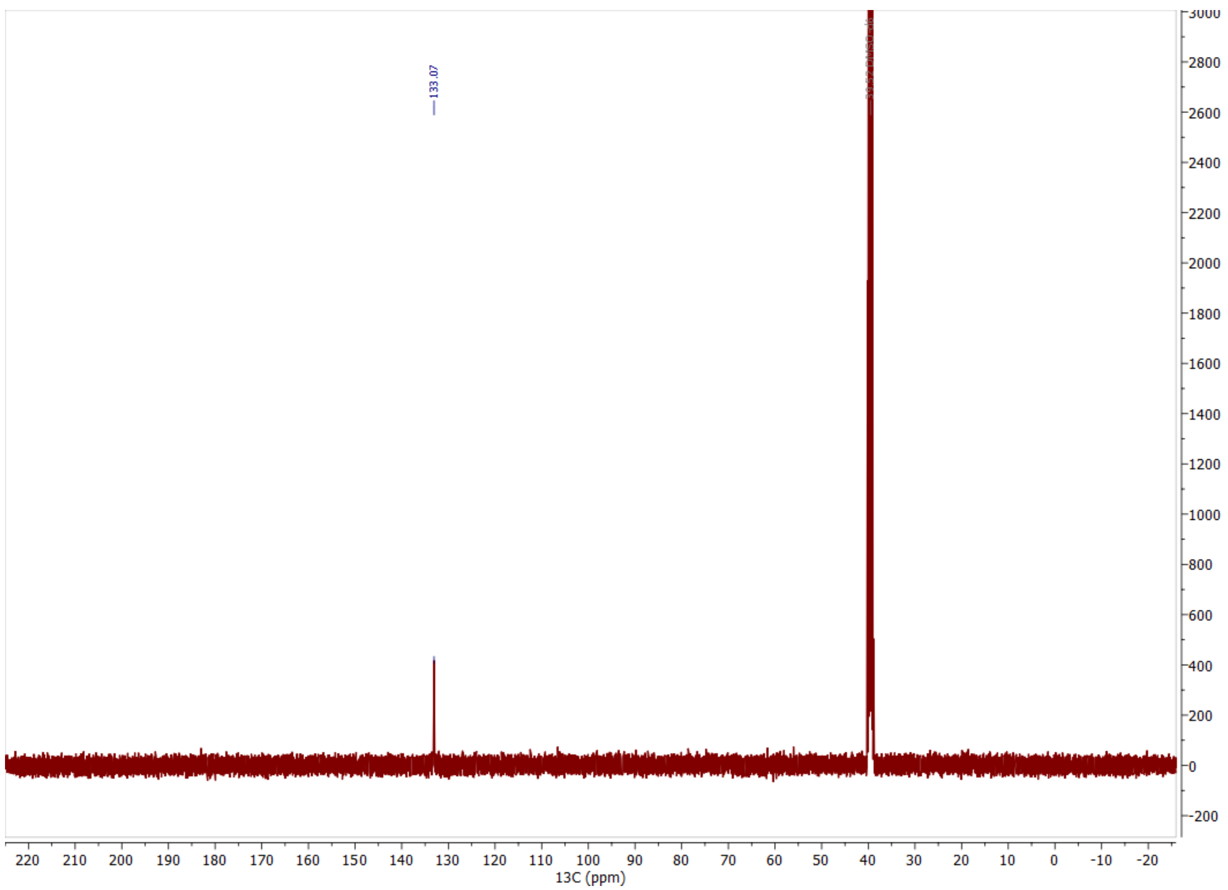

Figure S8. $^{13}C\{^{1}H\}$ NMR spectrum of sodium 1-oxido-5*H*-tetrazolate (**2**) in DMSO-d_6

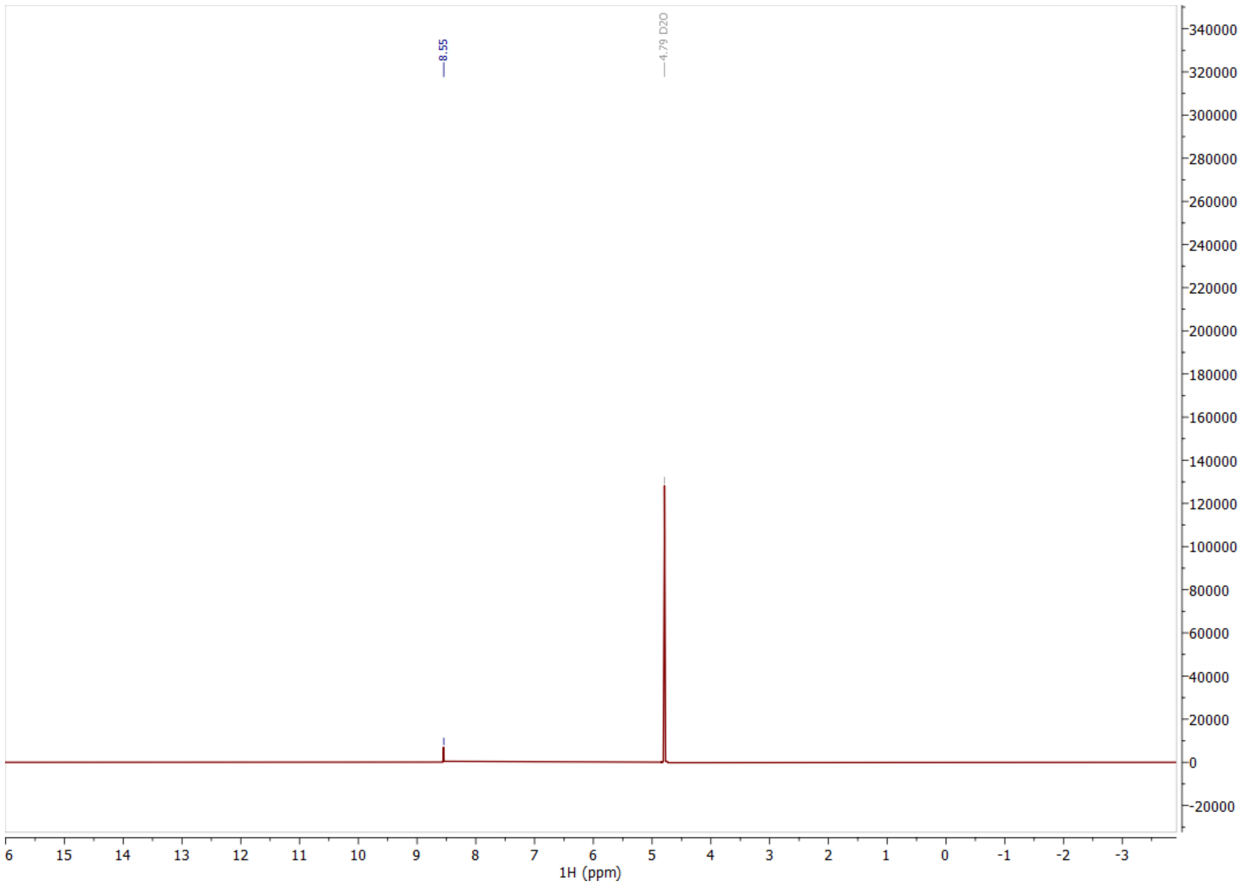

Figure S9. ^{1}H NMR spectrum of potassium 1-oxido-5*H*-tetrazolate (**3**) in D_2O.

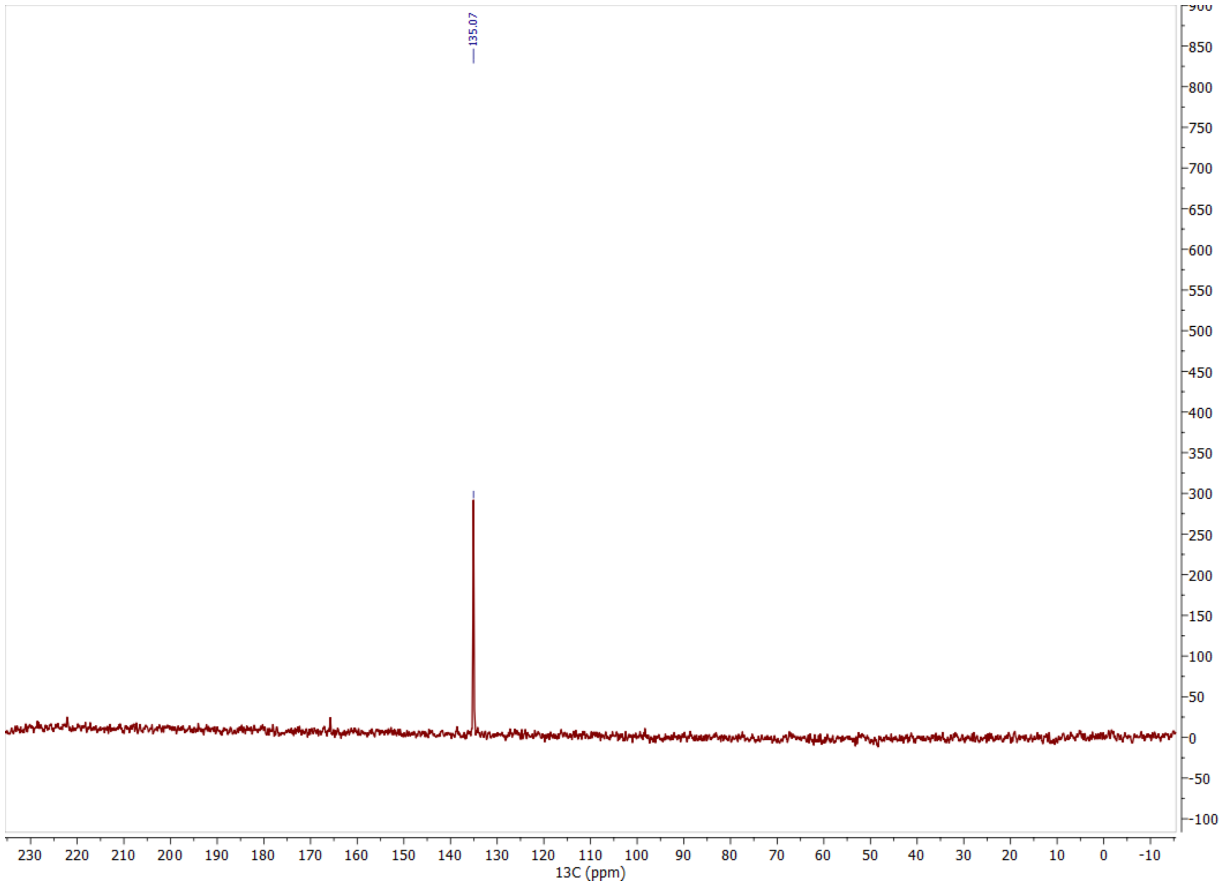

Figure S10. $^{13}C\{^{1}H\}$ NMR spectrum of potassium 1-oxido-5*H*-tetrazolate (**3**) in D_2O.

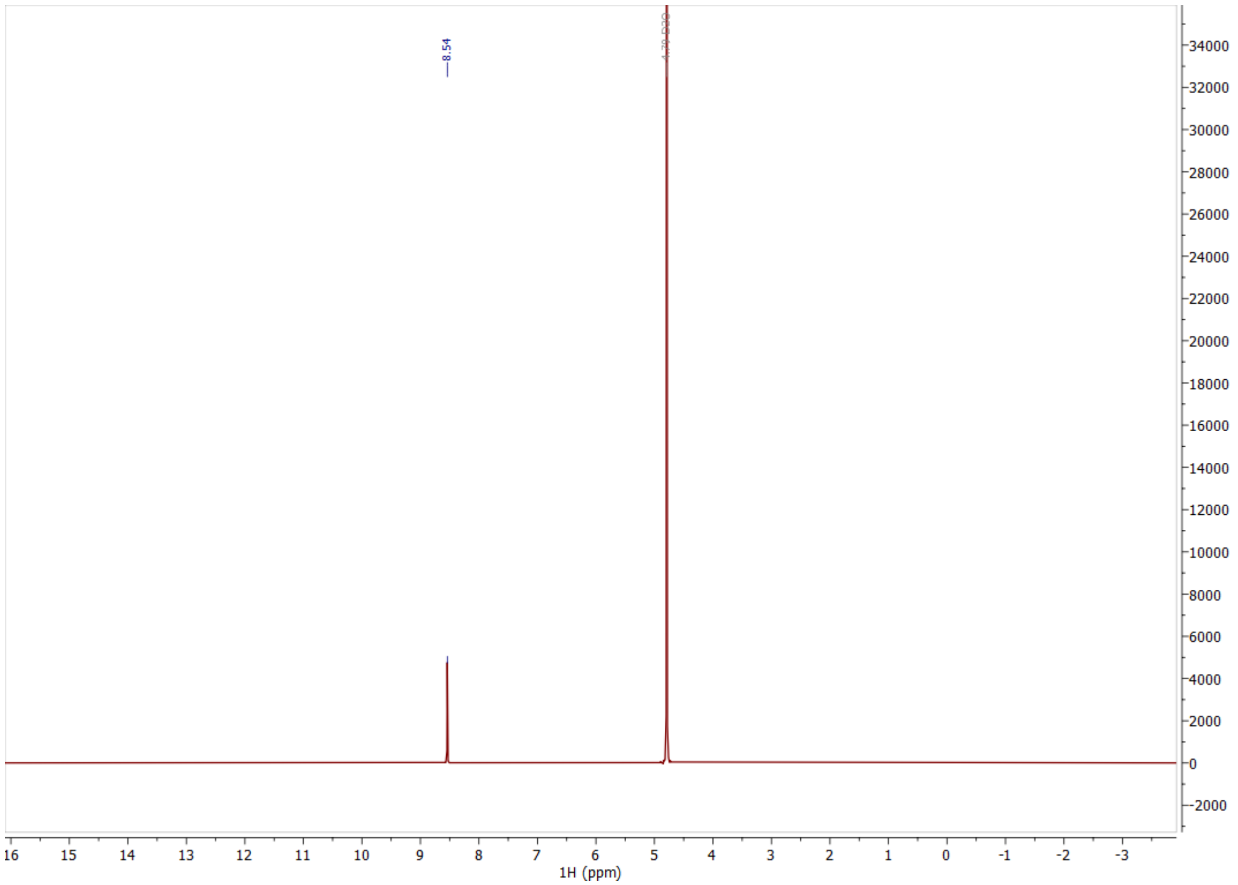

Figure S11. ^1H NMR spectrum of ammonium 1-oxido-5*H*-tetrazolate (**5**) in D_2O.

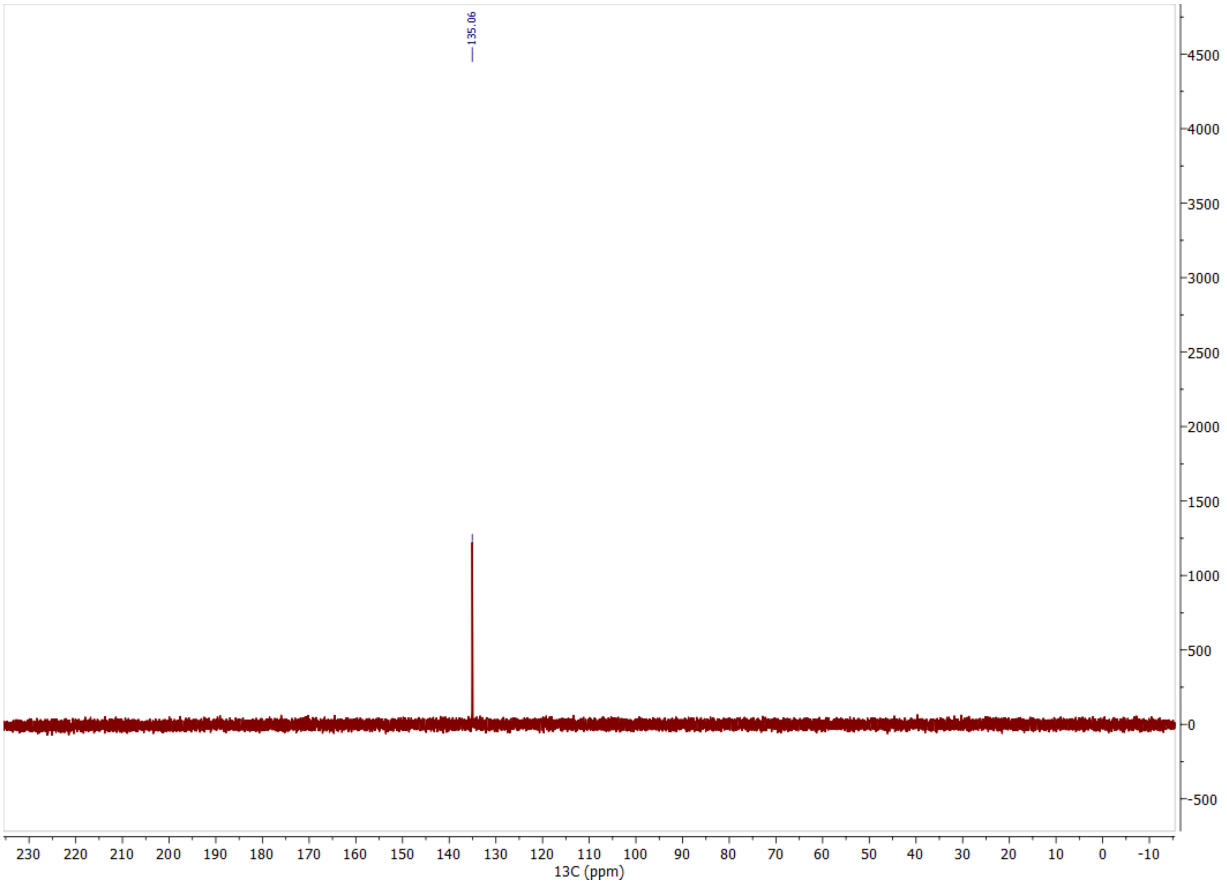

Figure S12. $^{13}C\{^1H\}$ NMR spectrum of ammonium 1-oxido-5*H*-tetrazolate (**5**) in D_2O.

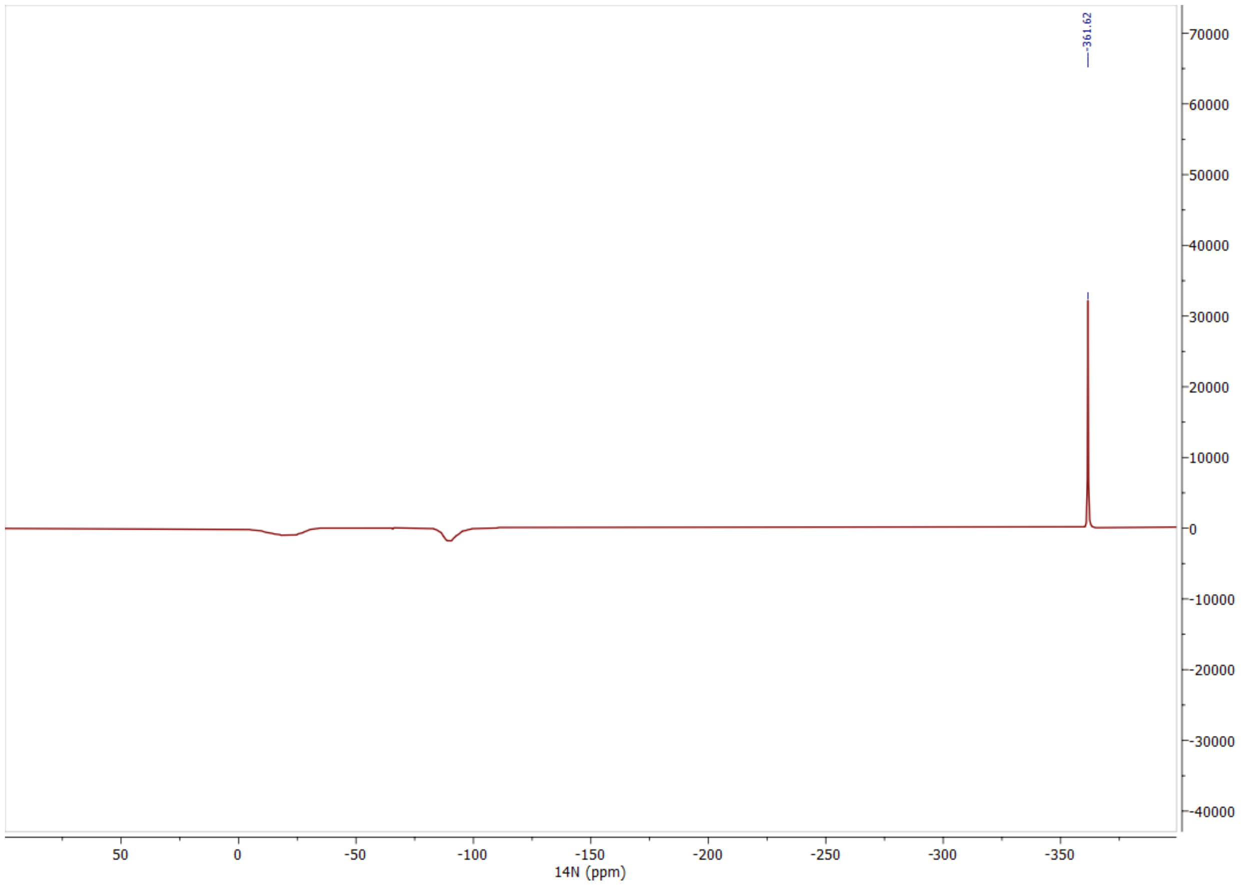

Figure S13. ^{14}N NMR spectrum of ammonium 1-oxido-5*H*-tetrazolate (**5**) in D_2O.

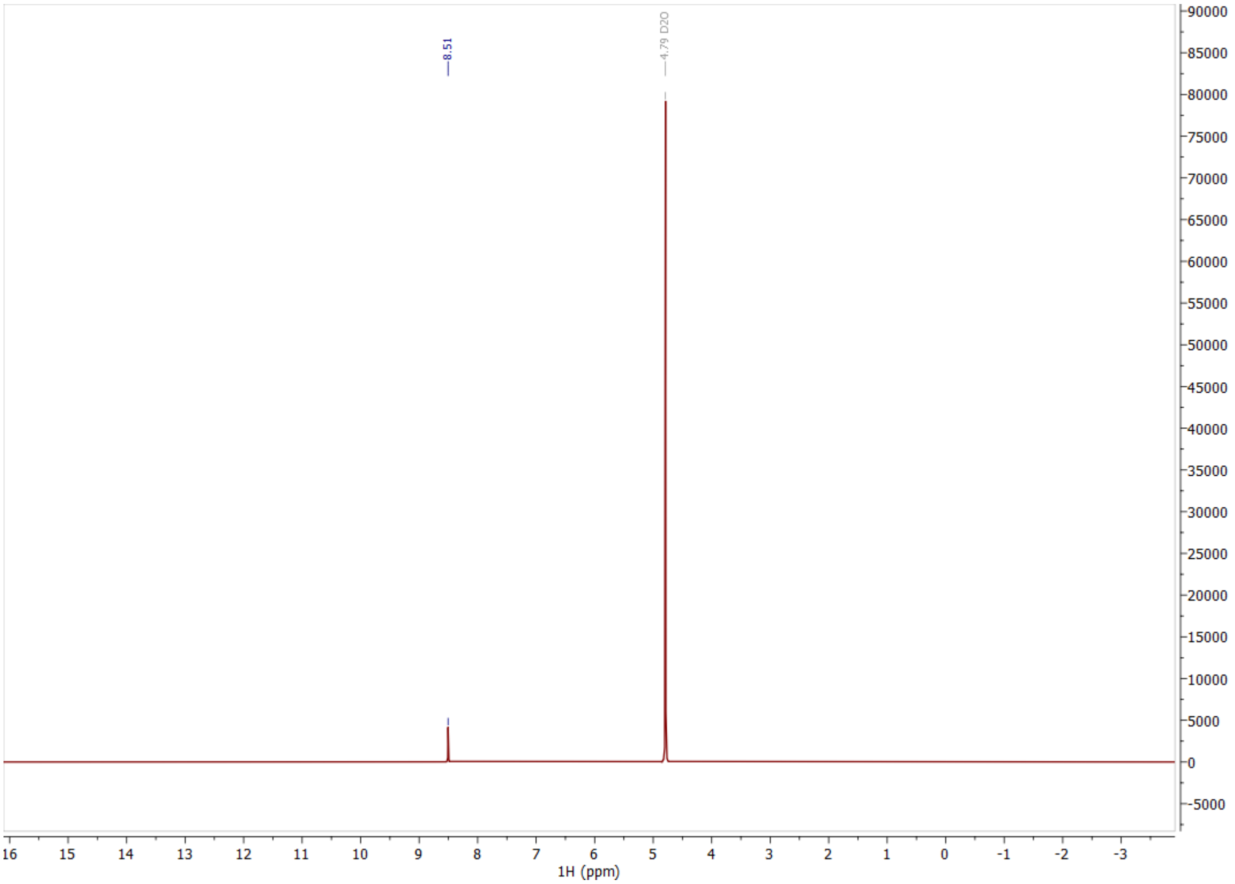

Figure S14. ^{1}H NMR spectrum of hydroxylammonium 1-oxido-5*H*-tetrazolate (**6**) in D_2O.

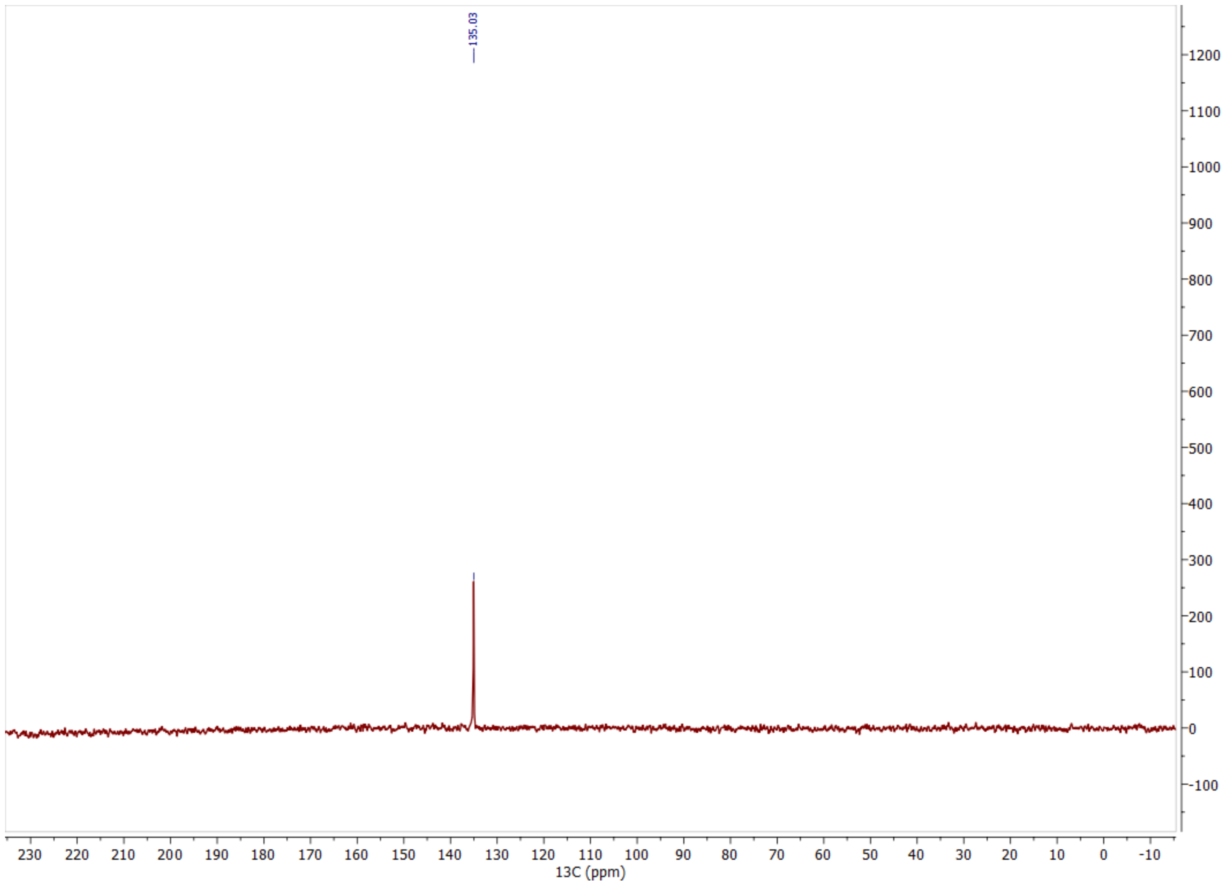

Figure S15. $^{13}C\{^{1}H\}$ NMR spectrum of hydroxylammonium 1-oxido-5*H*-tetrazolate (**6**) in D_2O.

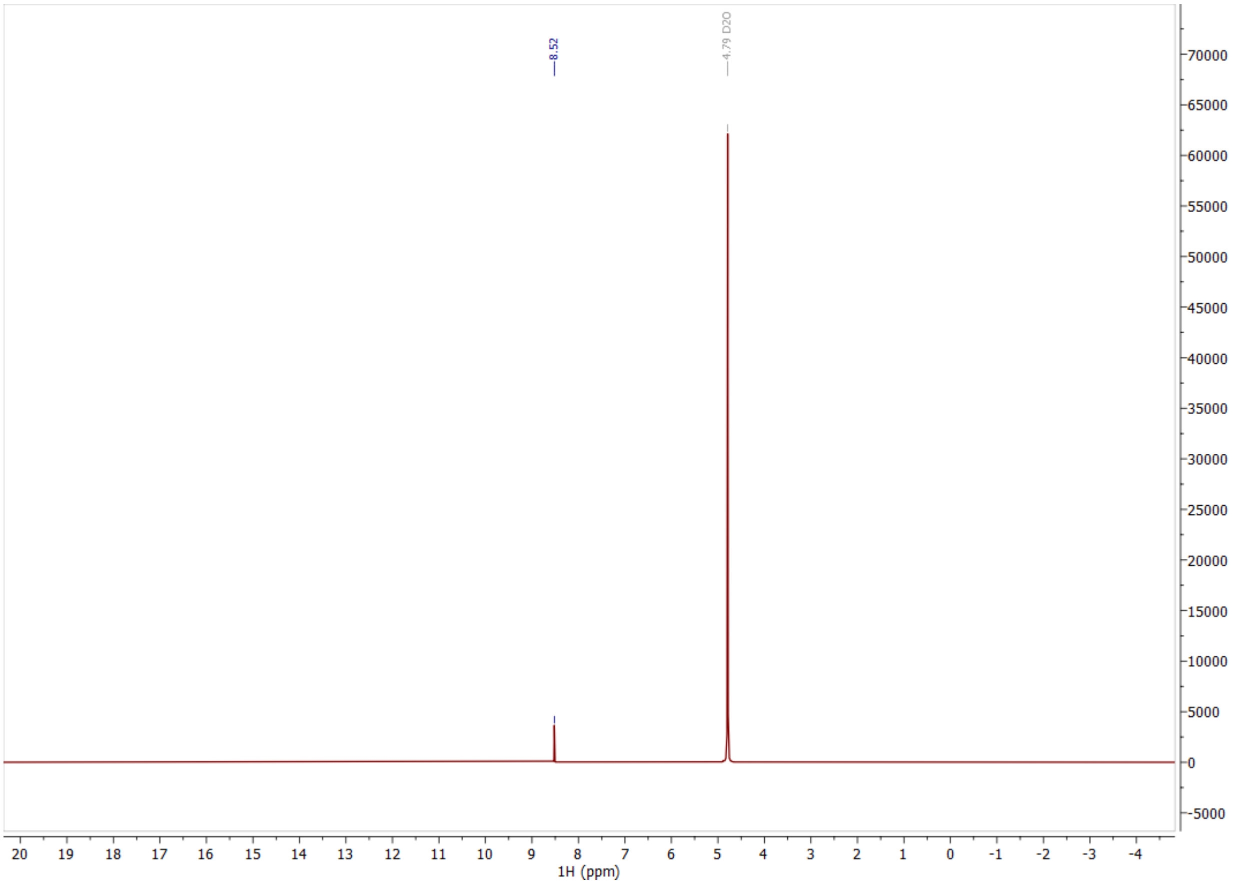

Figure S16. ^{1}H NMR spectrum of hydrazinium 1-oxido-5*H*-tetrazolate (**7**) in D_2O.

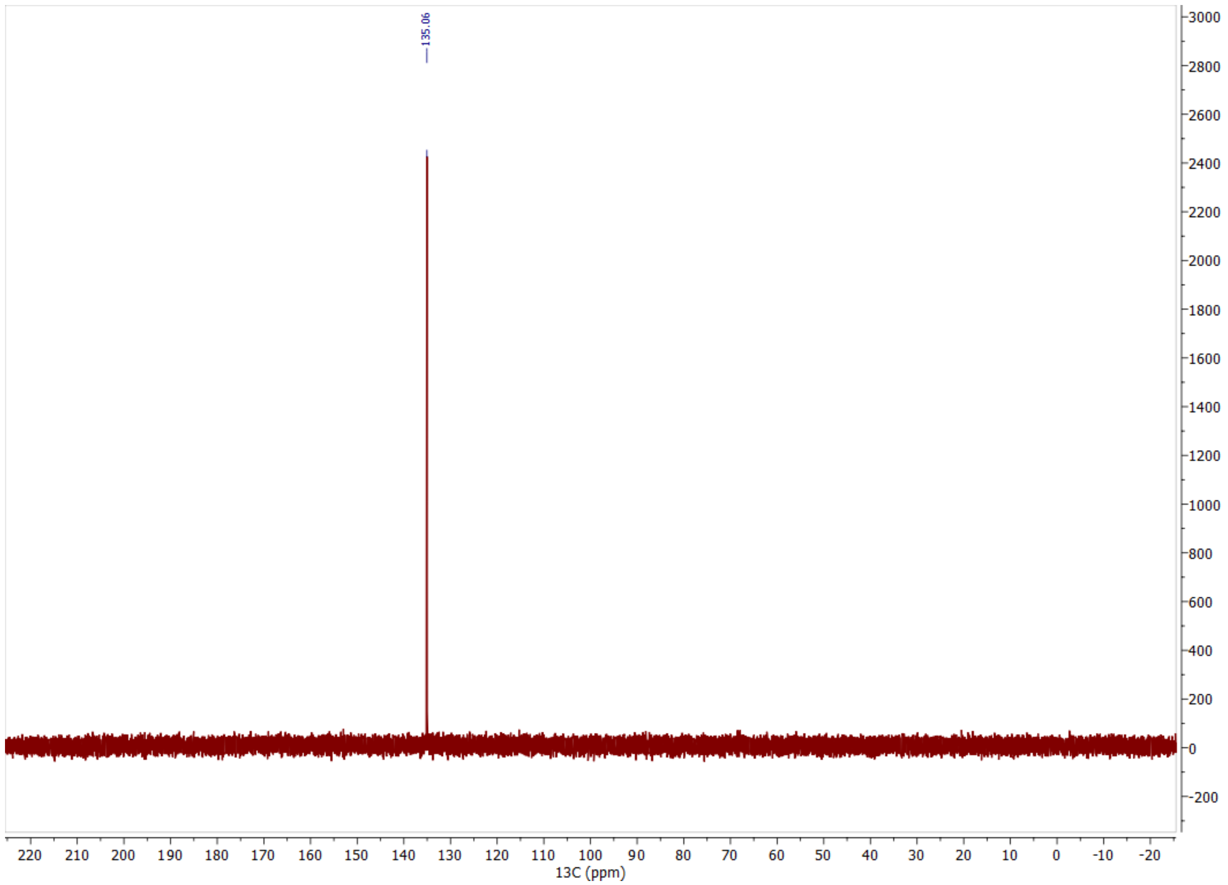

Figure S17. $^{13}C\{^{1}H\}$ NMR spectrum of hydrazinium 1-oxido-5*H*-tetrazolate (**7**) in D_2O.

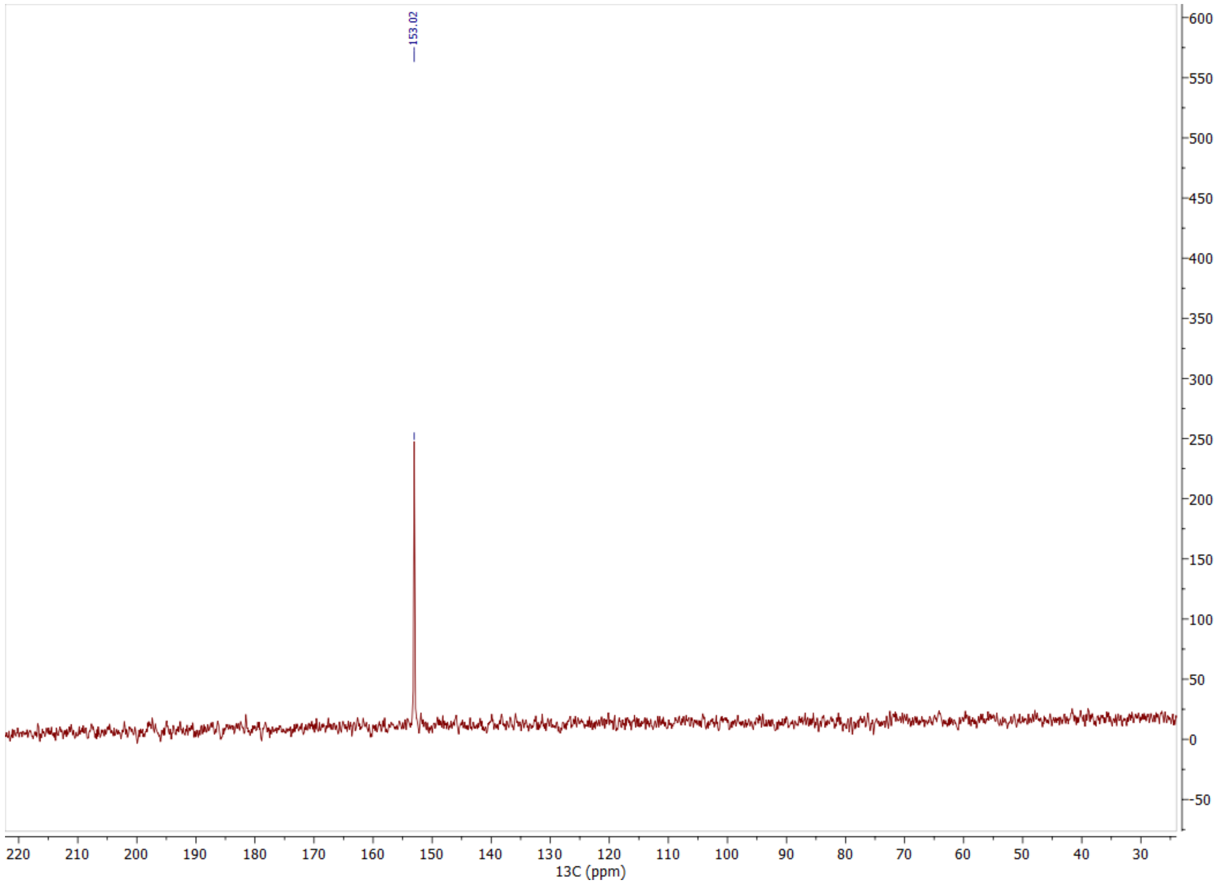

Figure S18. $^{13}C\{^{1}H\}$ NMR spectrum of Trilithium bis(1-oxidotetrazol-5-yl)triazenide (**9**) in D_2O.

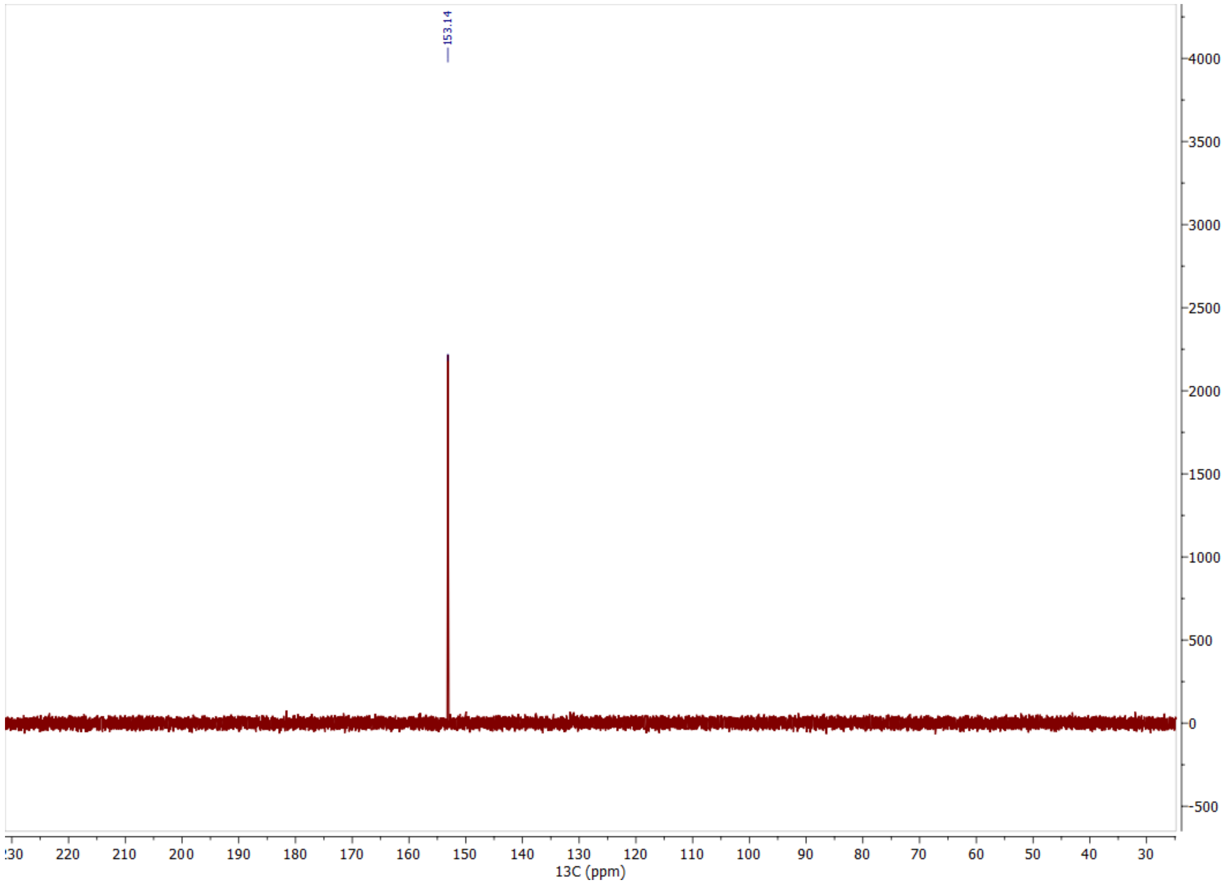

Figure S19. $^{13}C\{^{1}H\}$ NMR spectrum of Trisodium bis(1-oxidotetrazol-5-yl)triazenide (**10**) in D_2O.

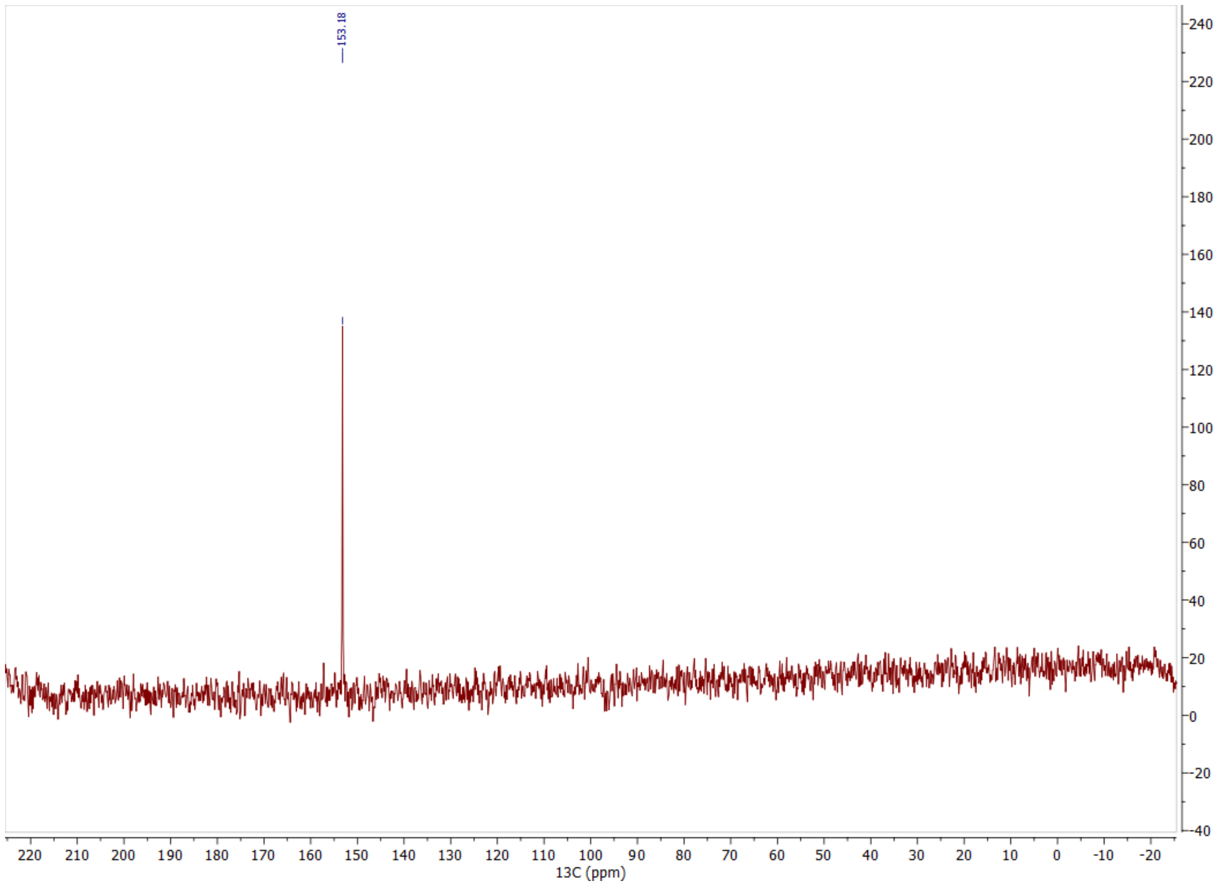

Figure S20. $^{13}C\{^{1}H\}$ NMR spectrum of Tripotassium bis(1-oxidotetrazol-5-yl)triazenide (**11**) in D_2O.

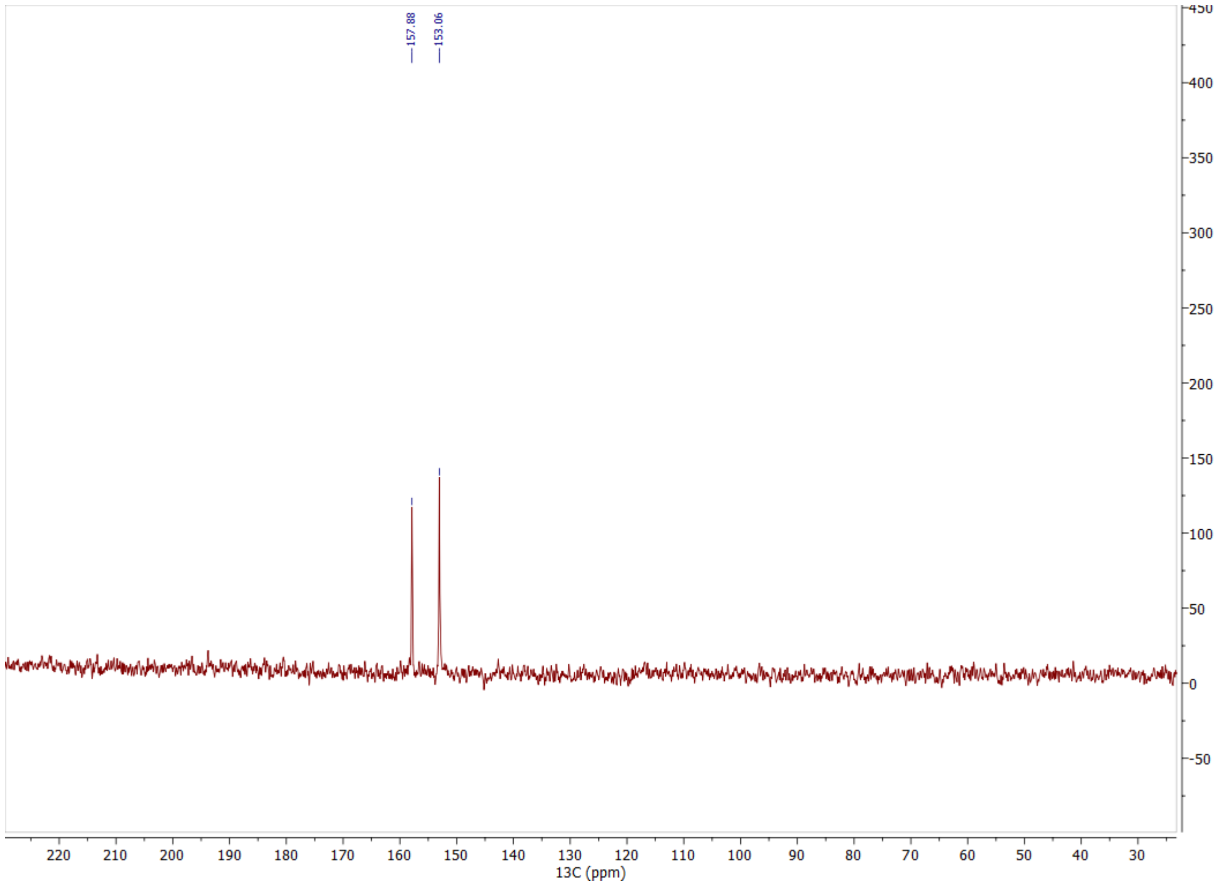

Figure S21. $^{13}C\{^{1}H\}$ NMR spectrum of Triguanidinium bis(1-oxidotetrazol-5-yl)triazenide (**13**) in D_2O.

6. IR Spectroscopy of 1 – 15.

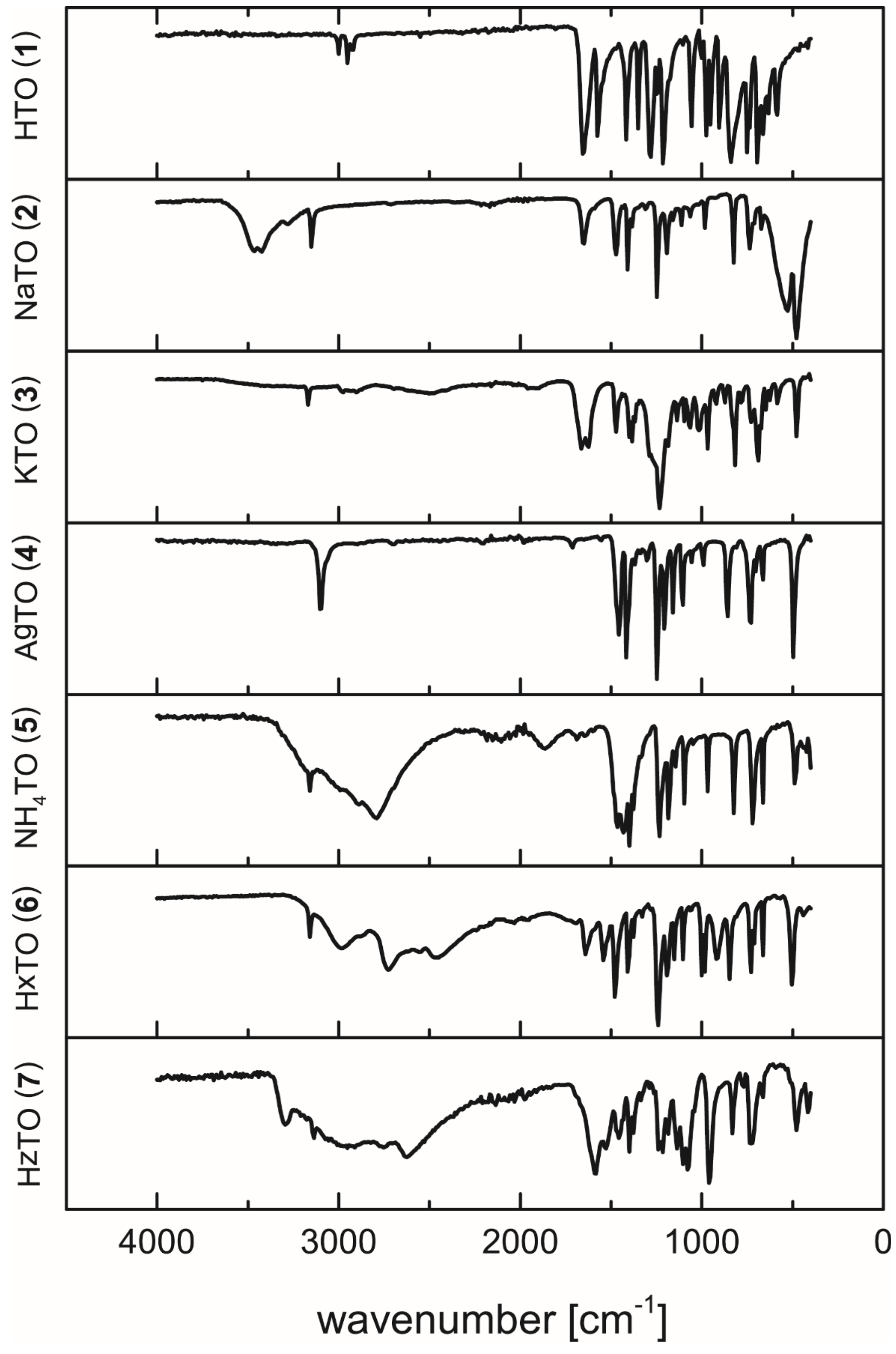

Figure S22. IR spectra of 1-hydroxytetrazole (**1**) and its salts **2**–**7**.

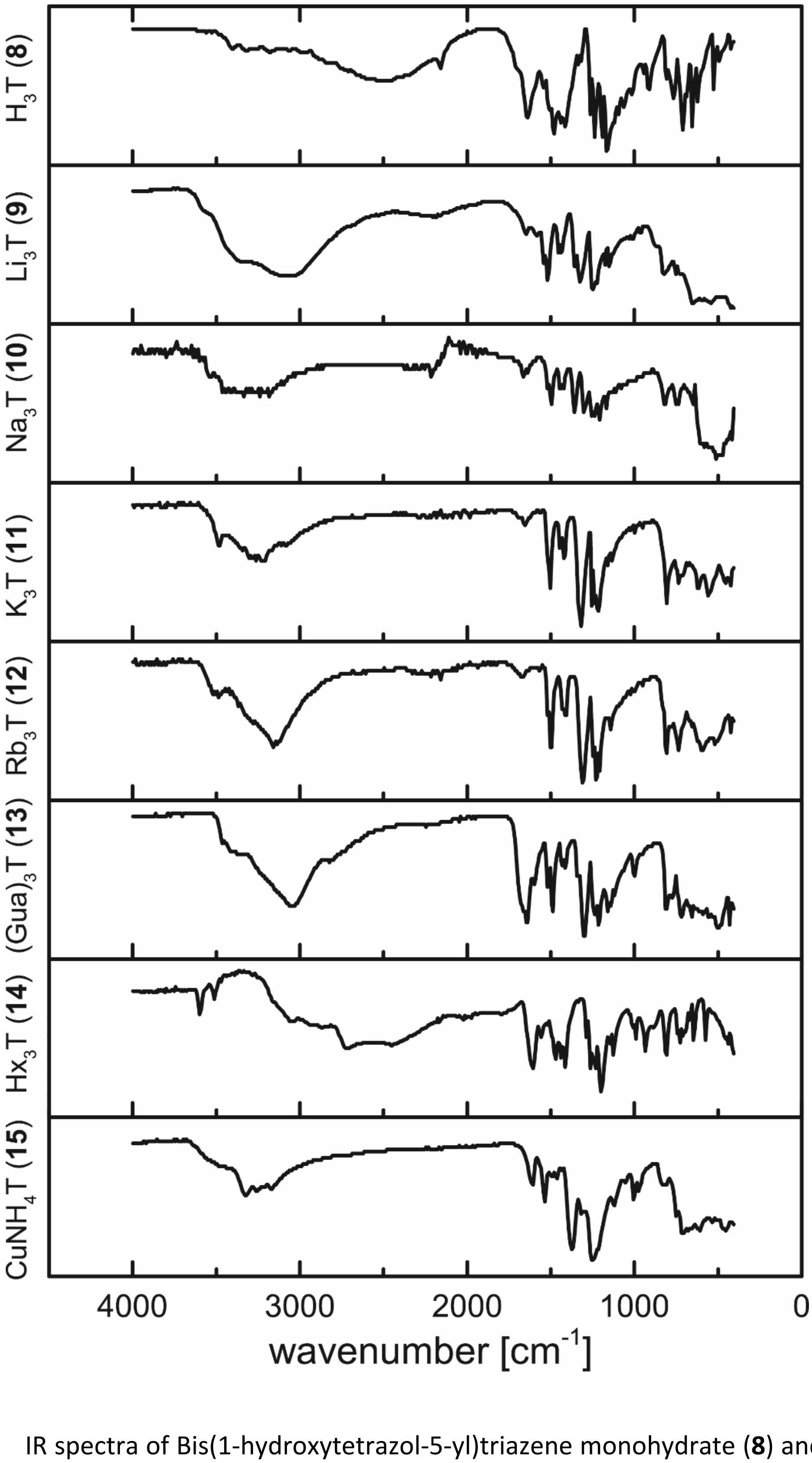

Figure S23. IR spectra of Bis(1-hydroxytetrazol-5-yl)triazene monohydrate (**8**) and its salts **9** – **15**. All salts except **14** are hexahydrates (**9**, **10**) or trihydrates (**11**-**13**, **15**)

7. General Methods

All chemicals and solvents were employed as received (Sigma-Aldrich, Fluka, Acros, ABCR). ^{1}H, $^{13}C\{^{1}H\}$, ^{14}N, spectra were recorded at ambient temperature using a JEOL Bruker 27400, Eclipse 270, JEOL EX 400 or a JEOL Eclipse 400 instrument. The chemical shifts quoted in ppm in the text refer to typical standards such as tetramethylsilane (^{1}H, ^{13}C) nitromethane (^{14}N,^{15}N) in DMSO-d_6, D_20 or acetone-d_6 as the solvent. Endothermic and exothermic events of the described compounds, which indicate melting, loss of crystal water or decomposition, are given as the extrapolated onset temperatures. The samples were measured in a range of 25–400 °C at a heating rate of 5 °C min^{-1} through differential thermal analysis (DTA) with an OZM Research DTA 552-Ex instrument and in some cases additionally by thermal gravimetric analysis (TGA) with a PerkinElmer TGA4000. Infrared spectra were measured with pure samples on a Perkin-Elmer BXII FT-IR system with a Smith DuraSampler IR II diamond ATR at a resolution of 4 cm^{-1} and 12 scans each. Spectra were analysed with the Perkin Elmer Spectrum 10 STD software suite.[S24] Determination of the carbon, hydrogen, and nitrogen contents was carried out by combustion analysis using an Elementar Vario El (nitrogen values determined are often lower than the calculated ones' due to their explosive behavior). Impact sensitivity tests were carried out according to STANAG 4489[S25] with a modified instruction[S26] using a BAM (Bundesanstalt für Materialforschung) drophammer.[S27] Friction sensitivity tests were carried out according to STANAG 4487[S30] with a modified instruction[S31] using the BAM friction tester.[S27,28] The classification of the tested compounds results from the "UN Recommendations on the Transport of Dangerous Goods".[S32,33] Additionally, all compounds were tested upon the sensitivity toward electrical discharge using the OZM Electric Spark XSpark10 device.[S28] Energetic properties have been calculated with the EXPLO5 6.05.04 computer code[S34] using the, to RT converted, X-ray density and calculated solid state heats of formation. These were computed by the atomization method as described in recently published papers. Electronic enthalpies were calculated with the Gaussian09 software[S13] suite using the CBS-4M method.

8. References

S1 CrysAlisPRO (Version 171.33.41), Oxford Diffraction Ltd., 2009.

S2 A. Altomare, G. Cascarano, C. Giacovazzo, and A. Guagliardi, *J. Appl. Crystallogr.*, 1992, **26**, 343.

S3 A. Altomare, G. Cascarano, C. Giacovazzo, A. Guagliardi, A. G. G. Moliterni, M. C. Burla, G. Polidori, M. Camalli and R. Spagna, SIR97, 2003.

S4 A. Altomare, M. C. Burla, M. Camalli, G. L. Cascarano, C. Giacovazzo, A. Guagliardi, A. G. G. Moliterni, G. Polidori and R. Spagna, *J. Appl. Crystallogr.*, 1999, **32**, 115.

S5 G. M. Sheldrick, SHELXL-97, University of Göttingen, Germany, 1997.

S6 G. M. Sheldrick, *Acta Crystallogr. Sect. A*, 2008, **64**, 112.

S7 G. M. Sheldrick, *Acta Cryst. A*, 2015, **71**, 3–8.

S8 A. L. Spek, PLATON, Utrecht University, The Netherlands, 1999.

S9 L.J. Farrugia, *J. Appl. Cryst.*, 2012, **45**, 849.

S10 O. V. Dolomanov, L. J. Bourhis, R. J. Gildea, J. A. K. Howard and H. Puschmann, *J. Appl. Cryst*., 2009, **42**, 339–341.

S11 Empirical absorption correction using spherical harmonics, implemented in SCALE3 ABSPACK scaling algorithm (CrysAlisPro Oxford Diffraction Ltd., Version 171.33.41, 2009).

S12 APEX3, Bruker AXS Inc., Madison, Wisconsin, USA.

S13 M. J. Frisch, G. W. Trucks, H. B. Schlegel, G. E. Scuseria, M. A. Robb, J. R. Cheeseman, G. Scalmani, V.Barone, B. Mennucci, G. A. Petersson, H. Nakatsuji, M. Caricato, X. Li, H.P. Hratchian, A. F. Izmaylov, J. Bloino, G. Zheng, J. L. Sonnenberg, M. Hada, M. Ehara, K. Toyota, R. Fukuda, J. Hasegawa, M. Ishida, T. Nakajima, Y. Honda, O. Kitao, H. Nakai, T. Vreven, J. A. Montgomery, Jr., J. E. Peralta, F. Ogliaro, M. Bearpark, J. J. Heyd, E. Brothers, K. N. Kudin, V. N. Staroverov, R. Kobayashi, J. Normand, K. Raghavachari, A. Rendell, J. C. Burant, S. S. Iyengar, J. Tomasi, M. Cossi, N. Rega, J. M. Millam, M.Klene, J. E. Knox, J. B. Cross, V. Bakken, C. Adamo, J. Jaramillo, R. Gomperts, R. E. Stratmann, O. Yazyev, A. J. Austin, R. Cammi, C. Pomelli, J. W. Ochterski, R. L. Martin, K. Morokuma, V. G. Zakrzewski, G. A. Voth, P. Salvador, J. J. Dannenberg, S. Dapprich, A. D. Daniels, O. Farkas, J.B. Foresman, J. V. Ortiz, J. Cioslowski and D. J. Fox, Gaussian 09 A.02, Gaussian, Inc., Wallingford, CT, USA, 2009.

S14 J. W. Ochterski, G. A. Petersson and J. A. Montgomery Jr., *J. Chem. Phys.*, 1996, **104**, 2598–2619.

S15 J. A. Montgomery Jr., M. J. Frisch, J. W. Ochterski and G. A. Petersson, *J. Chem. Phys.*, 2000, **112**, 6532–6542.

S16 L. A. Curtiss, K. Raghavachari, P. C. Redfern and J. A. Pople, *J. Chem. Phys.*, 1997, **106**, 1063–1079.

S17 E. F. C. Byrd and B. M. Rice, *J. Phys. Chem. A*, 2006, **110**, 1005–1013.

S18 B. M. Rice, S. V. Pai and J. Hare, *Comb. Flame*, 1999, **118**, 445–458.

S19 P. J. Lindstrom and W. G. Mallard, NIST Standard Reference Database Number 69, http://webbook.nist.gov/chemistry/, (accessed March 2021).

S20 M. S. Westwell, M. S. Searle, D. J. Wales and D. H. Williams, *J. Am. Chem. Soc*. 1995, **117**, 5013–5015.

S21 F. Trouton, *Philos. Mag.* 1884, **18**, 54–57.

S22 H. D. B. Jenkins, H. K. Roobottom, J.Passmore, L. Glasser, Inorg. Chem.1999, 38(16), 3609–3620.

S23 H. D. B. Jenkins, D. Tudela, L. Glasser, Inorg. Chem.2002, 41(9), 2364–2367

S24 PerkinElmer Inc., Spectrum™ 10 STD, Waltham, USA, **2010**.

S25 NATO standardization agreement (STANAG) on explosives, impact sensitivity tests, no. 4489, 1st ed., Sept. 17, 1999.

S26 WIWEB-Standardarbeitsanweisung 4-5.1.02, Ermittlung der Explosionsgefährlichkeit, hier der Schlagempfindlichkeit mit dem Fallhammer, Nov. 8, 2002.

S27 BAM, http://www.bam.de, (accessed March 2021).

S28 OZM, http://www.ozm.cz, (accessed March 2021).

S29 Military Standard 1751A (MIL-STD-1751A): safety and performance tests for qualification of explosives (high explosives, propellants and pyrotechnics), method 1016, Dec. 11, 2001.

S30 NATO standardization agreement (STANAG) on explosive, friction sensitivity tests, no. 4487, 1st ed., Aug. 22, 2002.

S31 WIWEB-Standardarbeitsanweisung 4-5.1.03, Ermittlung der Explosionsgefährlichkeit oder der Reibeempfindlichkeit mit dem Reibeapparat, Nov. 8, 2002.

S32 UN Model Regulation: Recommendations on the Transport of Dangerous Goods – Manual of Tests and Criteria, section 13.4.2.3.3, 2015.

S33 Impact: insensitive > 40 J, less sensitive ≥ 35 J, sensitive ≥ 4 J, very sensitive ≤ 3 J; Friction: insensitive > 360 N, less sensitive = 360 N, sensitive < 360 N and > 80 N, very sensitive

≤ 80 N, extremely sensitive ≤ 10 N. According to the UN Recommendations on the Transport of Dangerous Goods, 5th ed., 2009.

S34 M. Sućeska, EXPLO5 Version 6.05 User's Guide. Zagreb, Croatia: OZM; 2018.
